# Cellulose-Supported Heterogeneous Gold-Catalyzed Cycloisomerization Reactions of Alkynoic Acids and Allenynamides

**DOI:** 10.1021/acscatal.3c02722

**Published:** 2023-07-25

**Authors:** Luca Deiana, Elham Badali, Abdolrahim A. Rafi, Cheuk-Wai Tai, Jan-E Bäckvall, Armando Córdova

**Affiliations:** † Department of Natural Sciences, Mid Sweden University, Holmgatan 10, SE-85179 Sundsvall, Sweden; ‡ Department of Materials and Environmental Chemistry, Arrhenius Laboratory, Stockholm University, SE-10691 Stockholm, Sweden; § Department of Organic Chemistry, Arrhenius Laboratory, Stockholm University, SE-10691 Stockholm, Sweden

**Keywords:** cellulose-supported nanogold catalysis, C−C bond formation, heterogeneous catalysis, cycloisomerization, heterocycles, Alder-ene reaction

## Abstract

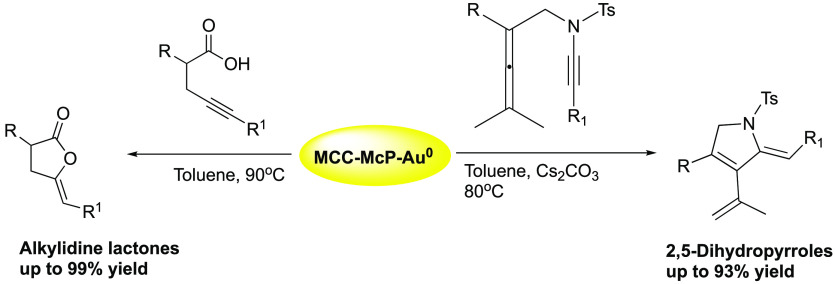

Herein, we describe efficient nanogold-catalyzed cycloisomerization reactions of alkynoic acids and allenynamides to enol lactones and dihydropyrroles, respectively (the latter via an Alder-ene reaction). The gold nanoparticles were immobilized on thiol-functionalized microcrystalline cellulose and characterized by electron microscopy (HAADF-STEM) and by XPS. The thiol-stabilized gold nanoparticles (Au^0^) were obtained in the size range 1.5–6 nm at the cellulose surface. The robust and sustainable cellulose-supported gold nanocatalyst can be recycled for multiple cycles without losing activity.

The development of robust and selective heterogeneous catalytic processes is fundamental for the future of our planet since the possibility of recycling the catalysts after the reaction completion opens up the possibility for highly sustainable processes avoiding the presence of metal pollutant in the reaction wastes.^1^ Common support materials for metal catalysts are carbon, alumina, and silica as well as polymeric materials.^2^ Natural polymers have received great attention from the scientific community due to their abundance, biocompatibility, biodegradability, and nontoxicity.^3^ Cellulose is the most abundant macromolecule in the world. The use of polysaccharides as heterogeneous supports for metal catalysts offers many advantages, compared to other heterogeneous supports, such as increased absorption capacity and the presence of many hydroxyl groups that offers the possibility to anchor various functional groups.^4^

Lactones of enols are structural motifs of many drugs and natural products exhibiting high biological activity.^5^ They are also useful building blocks and versatile intermediates in the synthesis of complex molecules.^6^ Catalytic intramolecular cycloisomerization of alkynoic acids provides an easy atom-economical way for the synthesis of functionalized enol lactones. Different transition-metal complexes (Pd,^7^ Au,^8^ Ag,^9^ Pt^10^) have been shown to be able to catalyze the intramolecular cyclization reaction with high degrees of regio- and chemoselectivity.

Alder-ene reactions are powerful for the synthesis of functionalized heterocycles, such as pyrroles. Various homogeneous catalytic approaches have been reported. Thus, Pt,^11^ Au,^12^ and Ag^13^ were used to promote the cyclization of 1,6-allenynes for stereoselective synthesis of trienes. Bäckvall and co-workers reported numerous studies on the alkyne-assisted palladium-catalyzed oxidative carbocyclization of allenynes, where the nucleophilic attack on palladium by the allene and the subsequent alkyne insertion led to the construction of a variety of 5-membered-ring compounds.^14^ Recently, our groups tried to expand the synthetic protocol of allenyne carbocyclization to first-row transition-metal catalysts. In this context, we reported a novel cellulose-supported heterogeneous nanocopper-catalyzed carbocyclization of allenynamides to pyrroles proceeding via an Alder-ene pathway.^15a^

Gold catalysts have shown a great capability to activate π carbon–carbon bonds, especially related to alkynes and allenes, toward the addition of nucleophiles.^16^ Gold nanoparticles, due their heat and air stability, have attracted the interest of many researchers from different scientific fields.^17−19^ One of the pioneering studies on the synthesis of gold nanoparticles was done by Turkevich et al. in 1951, in which HAuCl_4_ was reduced by citrate.^18a^ In 1994, Brust and Schiffrin reported a fundamental protocol in two steps for the synthesis of thiol-stabilized gold nanoparticles. First, a solution of HAuCl_4_ was mixed with thiol ligands, leading to the reduction of the Au^III^ salt to Au^I^ thiolates. Next, the Au^I^ thiolates were further reduced by NaBH_4_ to generate gold nanoparticles.^18b^ In 2015, Bäckvall and co-workers reported a novel electrochemical method for the preparation of gold nanoparticles supported on thiol-functionalized MCF.^19^ The Au^III^ species were first reduced by a surface-confined redox reaction with the thiol ligands to form MCF-supported Au^I^ thiolates. The MCF-SH-Au^I^ intermediate was isolated and further reduced with NaBH_4_ to give MCF-supported gold nanoparticles.^19^ It was demonstrated that the nanoparticle size is directly related to the catalytic activity.

The immobilization of nanoparticles, on heterogeneous solid supports, offers an easy way to avoid the agglomeration during the reaction process stabilizing the catalytic activity.^20^ Various solid materials such as cellulose,^21^ carbon nanotubes,^22^ zeolites,^23^ mesocellular foam,^14e,19^ and metal oxides^24^ have been studied as heterogeneous supports for gold nanoparticles. In our groups, we have worked extensively on the preparation of novel heterogeneous catalyst systems for stereoselective synthesis^14e,25^ and on the construction of functional cellulose-based materials.^14f,15a,15f,15h,15i^ Based on our previous work, we decided to investigate cellulose–gold heterogeneous catalysts for application in selective organic synthesis. Herein, we report on a cellulose-supported gold nanocatalyst for promoting cycloisomerization of alkynoic acids and allenynamides to enol lactones and dihydropyrroles, respectively, in high yields. The sustainable catalyst was recyclable and was used for 9 cycles without losing efficiency or selectivity. A remarkable observation is that the Au(0) nanoparticles on cellulose catalyze reactions that usually require Au(I) as a catalyst.

We began our study by fabricating cellulose-supported catalysts. Thus, the gold nanoparticles were immobilized on microcrystalline cellulose (MCC)^26^ functionalized with mercaptopropyl (McP) silane or aminopropyl (AmP) silane groups. A schematic overview of the synthesis of the MCC-Au^0^ catalysts is provided in Scheme 1. First MCC-McP and MCC-AmP were prepared by organocatalytic silylation catalyzed by tartaric acid.^15f,15g^ Next, the functionalized MCC was dispersed in an aqueous solution where HAuCl_4_ had been previously added to furnish the MCC-Au precatalysts, respectively. In the case of mixing the thiol-functionalized cellulose (MCC-McP) with HAuCl_4_ (0.1 M HCl), *in situ* reduction of the Au by sulfur occurred, and MCC-McP-Au^0^/Au^I^ was formed with Au(0) being the dominant oxidation state as shown by X-ray photoelectron spectroscopy (XPS) (Figure 1c.) At the same time, the thiol groups at the MCC-McP surface were oxidized to S^VI^ (−SO_2_–, −SO_3_H) as determined by XPS (see the Supporting Information). In this context, oxidation of thiol groups linked to gold nanoparticles to sulfones can significantly improve their catalytic activity for enyne cyclizations.^12c^ Subsequent reduction of the MCC-Au precatalysts with NaBH_4_ gave access to the Au nanoparticle catalyst MCC-McP-Au^0^ (Figure 1d). The NaBH_4_ reduction of the MCC-AmP-Au precatalyst gave the corresponding MCC-AmP-Au^0^. The Au^0^ nanoparticles were characterized by scanning transmission electron microscopy (STEM), and the high-angle annular dark-field (HAADF) STEM images showed well-dispersed nanoparticles supported on MCC with a size range distribution of 1.5–6 nm (Figure 1a,b and Figure S1). The loadings of Au on MCC-McP-Au^0^ and on MCC-AmP-Au^0^ were 4.5% (w/w) and 16.3% (w/w), respectively, as determined by inductively coupled plasma optical emission spectroscopy (ICP-OES).

**Scheme 1:**
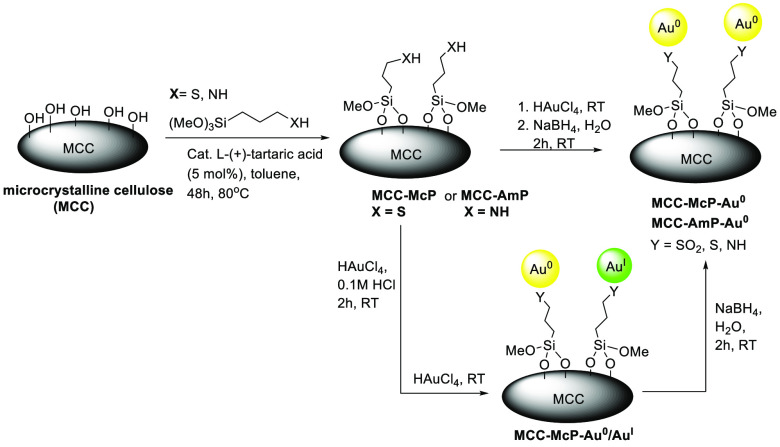
General Procedure for the Synthesis of MCC-Supported Gold Nanoparticle Catalysts

**Figure 1:**
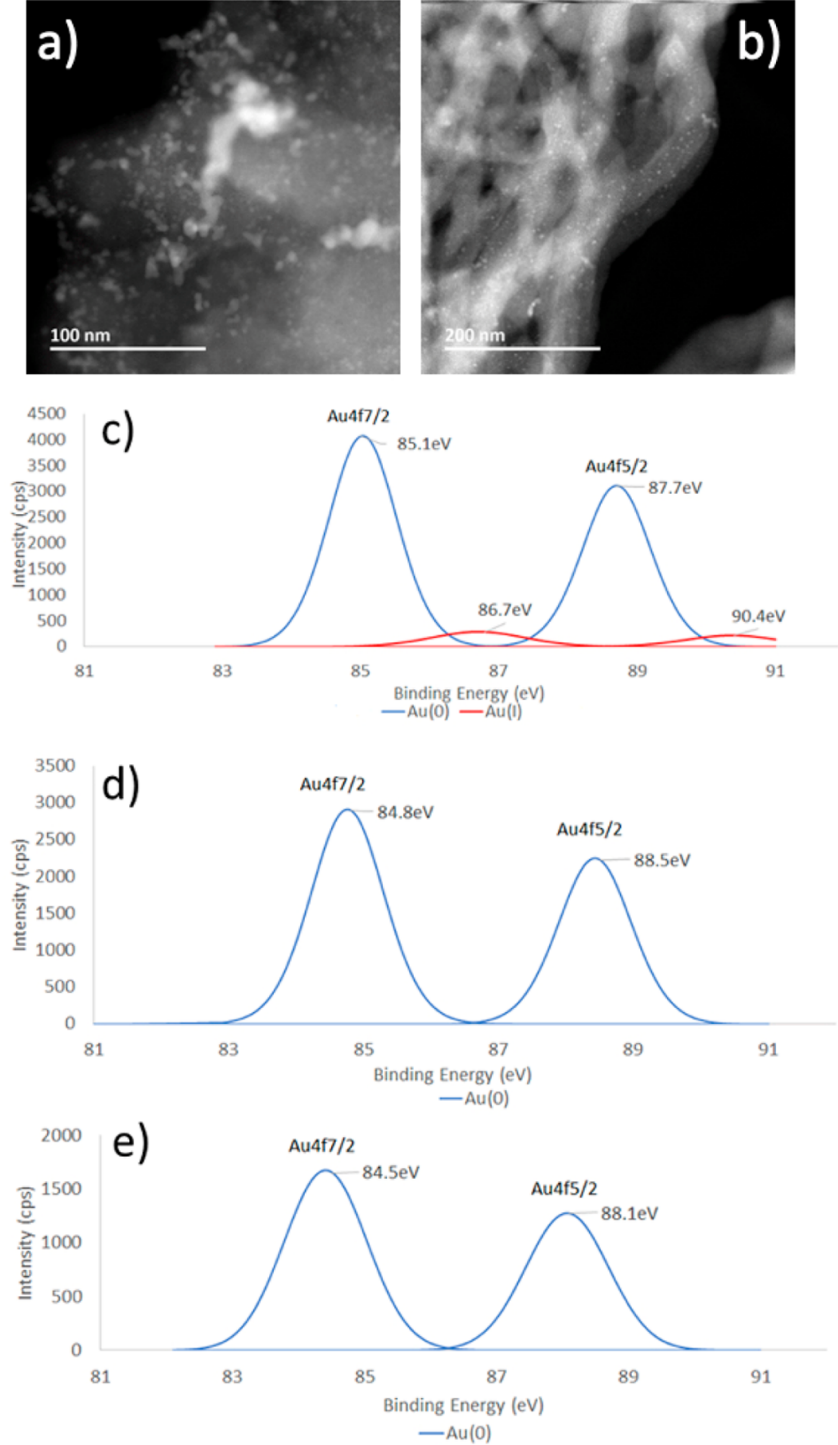
(a, b) HAADF-STEM images of well-dispersed Au nanoparticles on MCC-McP-Au^0^ at 100 and 200 nm scales, respectively. XPS spectra at the Au 4f (4f_5/2_ and 4f_7/2_) peaks of (c) MCC-McP-Au^0^/Au^I^, (d) MCC-McP-Au^0^, and (e) MCC-McP-Au^0^ after one reaction cycle.

With the cellulose-supported Au nanoparticle catalysts in hand, we began our investigations of catalytic intramolecular cyclization of alkynoic acids and allenenynamides. We started testing the efficiency of MCC-McP-Au^0^ nanocatalysts for the cycloisomerization of 4-pentynoic acid **1a**.

Different solvents were screened (Table 1), and running the reaction in toluene or acetonitrile at 90 °C, for 23 h, gave the corresponding product **2a** in quantitative yield. We also performed a control reaction with MCC-sulfonic acid propyl silane, which had been prepared by oxidation of MCC-McP with H_2_O_2_, using the exact same reaction conditions. The sulfonic acid modified MCC was not able to catalyze the formation of **2a** from **1a** (entry 6). We also tried to use MCC-McP-Au^0^/Au^I^, which is predominantly Au^0^ (Figure 1c). Surprisingly, under the standard conditions for lactonization of **1a** this catalyst gave only trace amounts of lactone **2a** (entry 7). With these results in hand, we performed the reaction on a variety of substrates using MCC-McP-Au^0^ (Scheme 2).^27^ α-substituted alkynoic acids with different functional groups gave the corresponding cyclic products **2b**–**f** in high yields. Difficult substrates such as hexynoic acid cyclized in 24 h, leading to the six-membered ring lactone **2d** in 99% yield. Amino acid derivative **1g** was also converted to the corresponding optically active α-amino lactone **2g** without loss of enantiopurity. Substrate **1h** terminally substituted with the alkyne acids gave the corresponding product **2h** in 80% yield with complete *Z* selectivity. Substrate **1i** with a rigid aromatic scaffold under these conditions cyclized in 18 h to give the corresponding benzofuran **2i** in 75% yield.

**Table 1:**
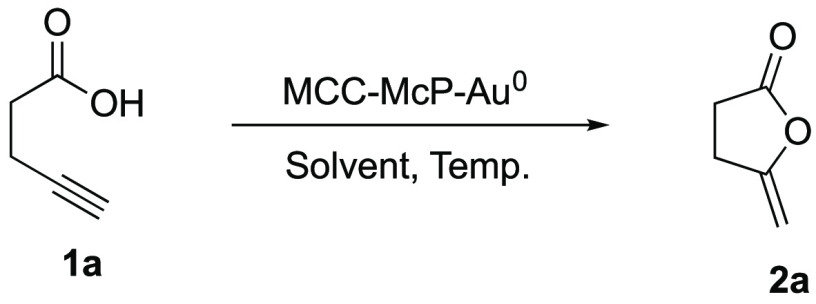
Condition Screening for the MCC-McP-Au^0^-Catalyzed Cycloisomerization of Alkynoic Acids^a^

entry	solvent	temp (°C)	time (h)	conversion (%)^b^
1	CH_2_Cl_2_	RT	96	33
2	CH_2_Cl_2_	40	24	20
3	toluene	90	23	99
4	dioxane	90	23	81
5	CH_3_CN	90	23	99
6^c^	toluene	90	23	0
7^d^	toluene	90	23	2

a Reaction conditions unless specified otherwise: **1a** (0.4 mmol), MCC-McP-Au^0^ (5 mg, 0.32 mol % Au^0^), solvent (1 mL).

b Conversion to **2a** as determined by ^1^H NMR using 1,4-dinitrobenzene as internal standard.

c Reaction performed with MCC-sulfonic acid propyl silane (MCC-Si-CH_2_CH_2_CH_2_SO_2_H).

d Reaction performed using MCC-McP-Au^0^/Au^I^ (cf. Figure 1c) as catalyst.

**Scheme 2:**
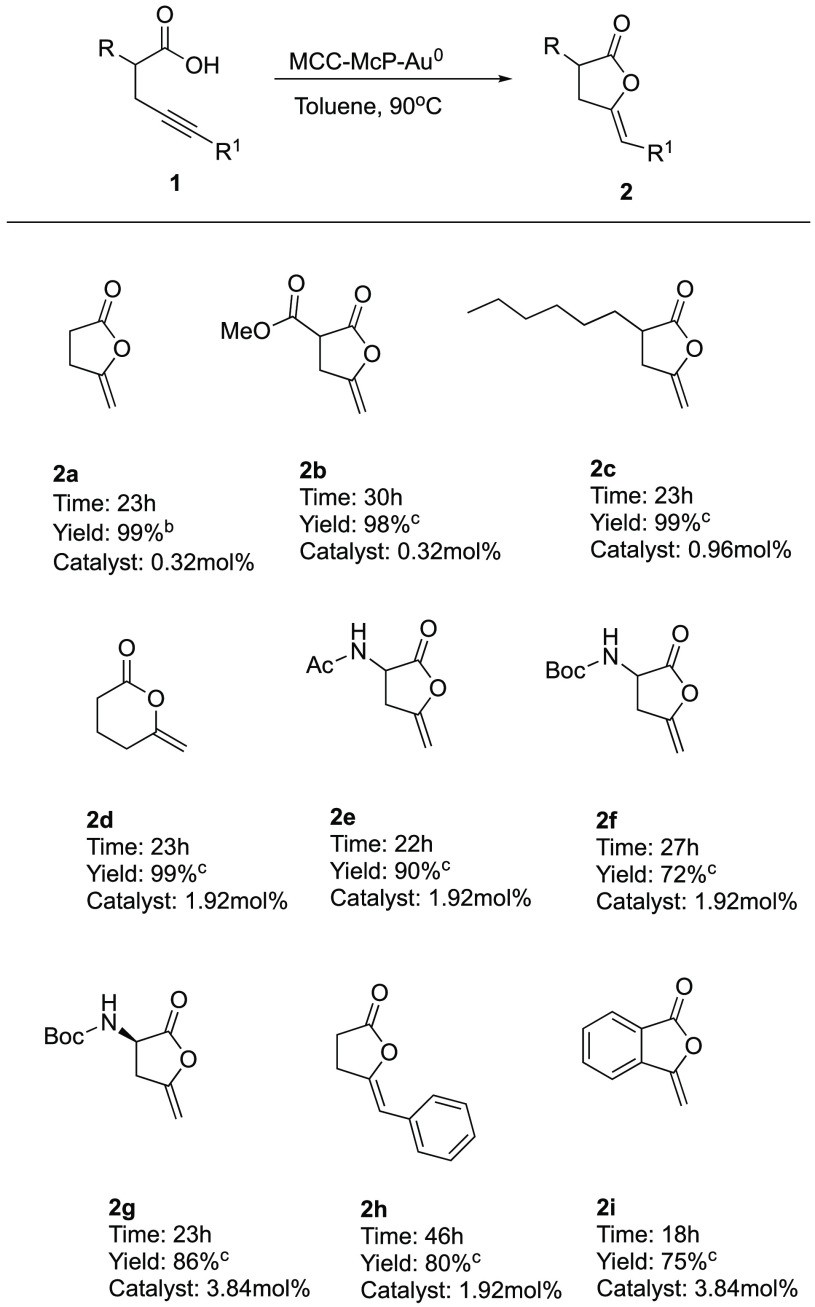
MCC-McP-Au^0^-Catalyzed Cycloisomerization of Different Alkynoic Acids Reaction conditions: **1a** (0.4 mmol), MCC-McP-Au^0^, toluene (1 mL), 90 °C. Determined by ^1^H NMR using 1,4-dinitrobenzene as internal standard. Isolated yield after flash chromatography.

We also carried out a few selected reactions with MCC-AmP-Au^0^ as a catalyst (Scheme 3). As can be seen from Scheme 3, results very similar to those in Scheme 2 were obtained. Also, here substitution at the terminal position of the alkyne with an aromatic compound (*p*-CF_3_-C_6_H_4_, **1j**) afforded the *Z* isomer **2j** in 73% yield.

**Scheme 3:**
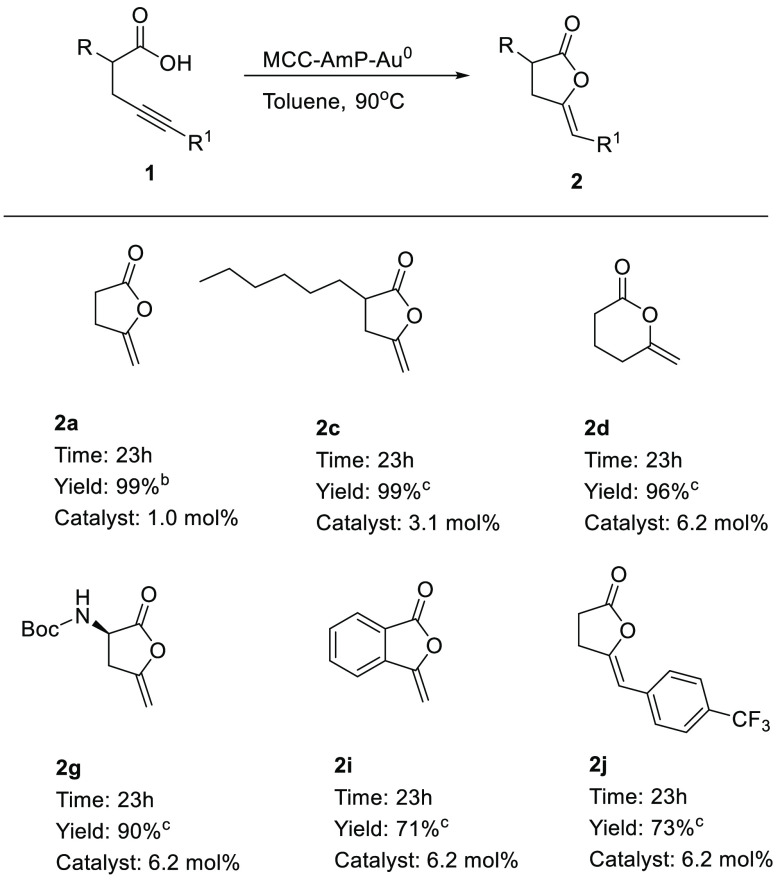
MCC-AmP-Au^0^-Catalyzed Cycloisomerization of Different Alkynoic Acids Reaction conditions: **1a** (0.4 mmol), MCC-AmP-Au^0^, toluene (1 mL), 90 °C. Determined by ^1^H NMR using 1,4-dinitrobenzene as internal standard. Isolated yield after flash chromatography.

Since lifetime and recycling of heterogeneous catalysts have key roles in practical applications, we evaluated the efficiency of the heterogeneous nanogold catalyst by conducting recycling experiments on the cycloisomerization of 4-pentynoic acid **1a** (Table 2).^28^ As shown, the catalyst was recycled 9 times without any evidence of loss in activity, giving the corresponding lactone **2a** in 99% NMR yield, within 23 h, for each cycle. We also performed XPS analysis of the recycled catalyst, which determined that Au(0) was the oxidation state of the gold surface of MCC-McP-Au^0^ (Figure 1e).

**Table 2:**
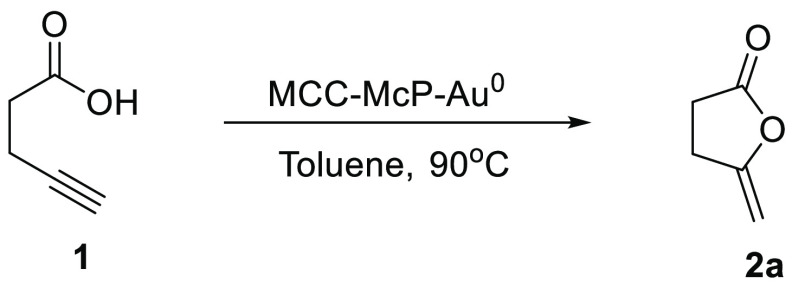
Recycling of MCC-McP-Au^0^ Catalyst^a^

cycle	time (h)	conversion (%)^b^
1	23	99
2	23	99
3	23	99
4	23	99
5	23	99
6	23	99
7	23	99
8	23	99
9	23	99

a Reaction conditions: **1** (0.4 mmol), MCC-McP-Au^0^ (20 mg, 1.28 mol %), toluene (1 mL), 90 °C.

b Determined by ^1^H NMR using 1,4-dinitrobenzene as internal standard.

Another fundamental aspect that makes heterogeneous catalysis so attractive for industrial use and for the development of green protocols is the possibility to avoid the accumulation of toxic industrial waste containing hazardous metal complexes. Thus, to determine the presence of free homogeneous Au species in solution during the reaction, hot filtration experiments were performed on the cycloisomerization of **1a** to **2a** (Scheme 2). MCC-McP-Au^0^ was filtered off after 20% conversion, and after that no more product **2a** was detected on attempted continued reaction. In a parallel control experiment, MCC-McP-Au^0^ was filtered off after full conversion. In both cases, inductively coupled plasma optical emission spectroscopy (ICP-OES) showed no presence of free Au species in the remaining solution (<0.5 ppm), confirming that there was no leaching of Au from MCC-McP-Au^0^.^29^

Also, in the MCC-AmP-Au^0^- catalyzed cycloisomerization of **1a** to **2a** (Scheme 3) the catalyst was filtered off after full conversion. In this case, ICP-OES analysis of the crude reaction mixture revealed a leaching of 7.5% of the total amount of Au used in this experiment. These results support the use of MCC-McP-Au^0^ as the preferred heterogeneous nanocatalyst for cycloisomerization transformations.

In cycloisomerization of alkynoic acids, e.g. **1** to **2**, catalyzed by homogeneous gold complexes, an Au(I) complex is the catalyst and it is proposed that an Au(I)-alkyne complex is attacked by the carboxylate group to give the lactone.^8c,8e^ Hydrolysis of the gold–carbon bond would give the product. The stereochemistry of the product when a disubstituted alkyne is used is in line with this mechanism. It is remarkable that the heterogeneous Au(0) catalyst MCC-McP-Au^0^, with no detectable amounts of Au(I) according to XPS, worked well as the catalyst. It is likely that a small number of Au(I) atoms are formed together with Au(0) in the Au(0) particles. The failure of MCC-McP-Au^0^/Au^I^ to catalyze the lactonization of **1a** to **2a** (entry 7, Table 1), may be due to the lack of nanoparticle formation in the latter material. This was supported by both HAADF-STEM images and Au L-edge EDS maps of the MCC-McP-Au^0^/Au^I^ (see Figure S2 in the Supporting Information). In contrast to MCC-McP-Au^0^, MCC-McP-Au^0^/Au^I^ shows evenly distributed Au(I) complexes on the MCC support, and no distinct nanoparticles were observed.

Motivated by the high yields detailed above and excellent recyclability of the MCC-McP-Au^0^ catalyst (Scheme 2 and Table 2), we decided to investigate the use of this catalyst for the conversion of allenynamide **3** to dihydropyrrole **4** (Scheme 4). We recently reported that nanocopper on MCC catalyzes this reaction via an Alder-ene cyclization mechanism.^15a^ Substrates bearing an aryl group at the R^1^ position of substrates **3** cyclized within 4 h and gave the corresponding aromatic allenynamides **4a**,**b** in 93% and 87% yields, respectively. Substrates **3c**,**d** with an aliphatic group at the R^1^ position react more slowly (23 h), giving the corresponding products **4c**,**d** in 71% and 85% yields, respectively.

**Scheme 4:**
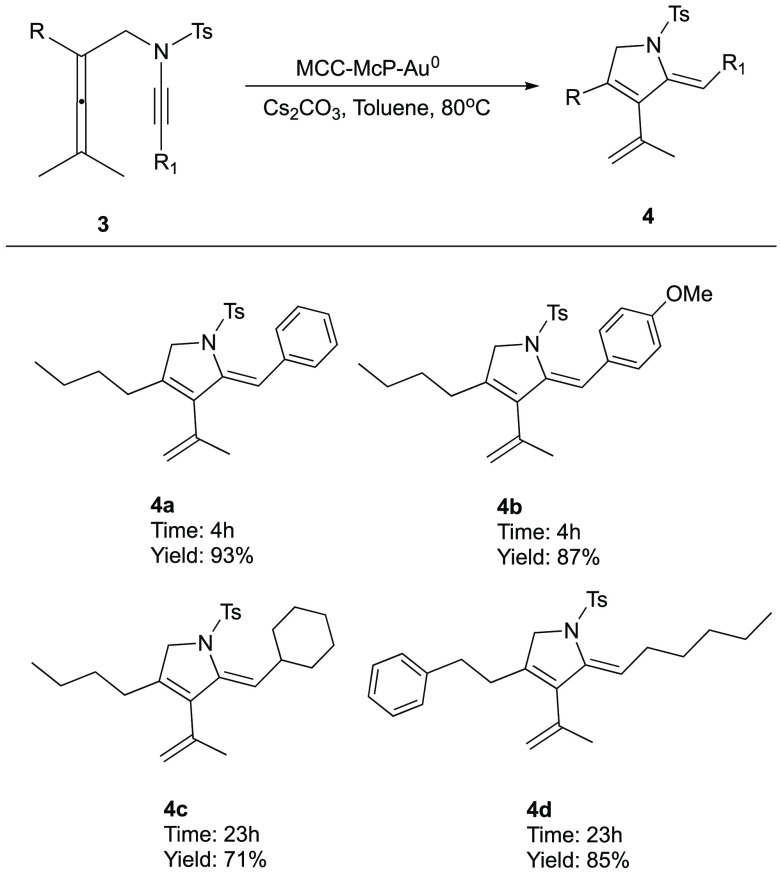
MCC-McP-Au^0^-Catalyzed Carbocyclization of Allenynamides Reaction conditions: **3** (0.15 mmol, 1 equiv), MCC-McP-Au^0^ (54 mg, 9 mol %), Cs_2_CO_3_ (0.195 mmol, 1.3 equiv), toluene (1 mL), 80 °C.

In conclusion, we have reported an efficient intramolecular cyclization of alkynoic acids to enol lactones and a stereoselective Alder-ene reaction of allenynamides to dihydropyrroles catalyzed by microcrystalline-cellulose-supported Au nanoparticles. The MCC-Au^0^ catalyst was shown to be highly selective for both reactions, leading to the corresponding cyclic products in high yields and high degrees of selectivity. The MCC-McP-Au^0^ catalyst displayed excellent recyclability without any loss of activity after 9 cycles, and no Au leaching in solution was detected. It is remarkable that the Au(0) catalyst MCC-McP-Au^0^ with no detectable amounts of Au(I) according to XPS catalyzes reactions that usually require Au(I) as catalyst. Further studies on the mechanism of these cellulose-based nanogold-catalyzed reactions are currently underway in our laboratories.

## Supporting Information Available

Detailed experimental procedures, STEM images, XPS spectra, FT-IR spectra, compound characterization data, and NMR spectra (PDF)
