# Supplementary material for: Cellulose-Supported Heterogeneous Gold-Catalyzed Cycloisomerization Reactions of Alkynoic Acids and Allenynamides
Source: ACS Catal. 2023 Jul 25;13(15):10418–24. doi: 10.1021/acscatal.3c02722 (PMC10407851; doi:10.1021/acscatal.3c02722)
Supplement: Supplementary file 1 — cs3c02722_si_001.pdf [file cs3c02722_si_001.pdf]

Supporting Information

Cellulose Supported Heterogeneous Gold-Catalyzed Cycloiso-merization Reactions of Alkynoic Acids and Allenynamides

Luca Deiana,[a] Elham Badali,[a] Abdolrahim A. Rafi,[a] Cheuk-Wai Tai,[b] Jan-E. Bäckvall,*[ac] and Armando Córdova*[b]

[a] Department of Natural Sciences, Mid Sweden University, Holmgatan 10, SE-85179, Sundsvall, Sweden

[b] Department of Materials and Environmental Chemistry, Arrhenius Laboratory, Stockholm University, SE-10691 Stockholm, Sweden

[c] Department of Organic Chemistry, Arrhenius Laboratory, Stockholm University, SE-10691 Stockholm, Sweden

E-mail: jeb@organ.su.se; armando.cordova@miun.se

Table of contents

General methods..……………………………………………………………………S2

Preparation of starting materials..………………………..……………………….S2

Preparation of catalysts…………………………………..……………………….S2

Experimental procedures……………………………………………………….….S4

Copies of spectra………………………………………………………………...…S10

Electron Microscopy data………………………………………….……………S49

FT-IR spectra of materials and catalysts……………………………………….……..S50

XPS spectra of materials and catalysts………………………………………………..S53

References……………………………………………………………………….....S81

General methods:

Chemicals and solvents were either purchased from commercial suppliers or purified by standards techniques. Avicel® PH-101 (~50 µm particle size) and $HAuCl_4*3H_2O$ were purchased from Aldrich and used as received. Alkynoic acids **1a**, **1c**, **1d**, and **1h** were obtained from commercial sources. Dry toluene was column-dried directly before use by a VAC: Solvent Purifier system. ^{1}H NMR spectra were recorded on a Bruker Avance 500 (500 MHz) spectrometer. Chemical shifts are reported in ppm from tetramethylsilane with the solvent resonance resulting from incomplete deuterium incorporation as the internal standard (CDCl3: δ 7.26 ppm). Data are reported as follows: chemical shift, multiplicity (s = singlet, d = doublet, q = quartet, br = broad, m = multiplet), and coupling constants (Hz), integration. ^{13}C NMR spectra were recorded on a Bruker Avance 500 (125.8 MHz) spectrometer with complete proton decoupling. Chemical shifts are reported in ppm from tetramethylsilane with the solvent resonance as the internal standard (CDCl3: δ 77.26 ppm). High-resolution mass spectrometry was performed on an Agilent Technologies 6520- Q-TOF ESI-MS (positive mode) at the Mid-Sweden University Mass Spectrometry Facility. Optical rotations were measured on a Perkin-Elmer 341 Polarimeter. Unless otherwise noted, all reactions were performed with distilled solvents in oven- dried (160°C) glassware. Elemental analyses on the Au content were carried out by Medac LTD Analytical and chemical consultancy services (United Kingdom) by ICP-OES. X-ray photoelectron spectroscopy was used to determine the structure and oxidation states of the Au nanoparticles. Infrared spectra were recorded by Thermo Scientific NICOLET 6700 FT-IR, Smart orbit, Diamond 30000-200 cm^{-1}. Scanning transmission electron microscopy (STEM) was carried out using a 200 kV JEOL 2100F microscope, which equips with a Schottky field-emission gun and ultrahigh-resolution pole-piece (Cs=0.5 mm). The samples in vials were shaken, in order to have small pieces. Then the samples were dispersed onto Cu TEM supporting grid with holey carbon films without using solvent. High-angle annular dark-field (HAADF) and bright-field (BF)-STEM images were acquired simultaneously using Gatan Microscopy Suite with the JEOL ADF and Gatan BF detectors, respectively. The camera length used was 8 cm.

Preparation of Alkynoic acids 1 and Allenynamides 3

Alkynoic acids **1b**[1] and **1e - 1g**,[2] and **1h - 1j**[3] were prepared according to literature procedures. Allenynamides **3a-3d** were prepared according to the procedures described in literature.[4]

MCC-McP synthetic procedure.

To a suspension of microcrystalline cellulose Avicel PH-101 (1 g, 6,17 mmol, 1 equiv) and L-(+)-tartaric acid (148 mg, 0,93 mmol, 5 mol% as compared to silane) in dry toluene (40 mL), was slowly added 3-(Trimethoxysilyl)-1-propanethiol (3.827 g, 18,52 mmol, 3 equiv). The reaction was stirred for 48 hours at 80 ^{o}C under nitrogen atmosphere. Next, the reaction mixture was cooled down to room temperature, transferred into an extraction thimble and washed more by Soxhlet extraction with acetone for 18 hours. MCC-McP was dried under vacuum to give a white powder (1.012 g).

MCC- McP-Au^0/Au^I synthetic procedure.

To a suspension of MCC-McP (183 mg) in HCl (5 mL, 0.1 M) was added, under vigorous stirring, $HAuCl_4$*$3H_2O$ (180 mg, 0.46 mmol). The reaction was stirred for 2 hours at room temperature. Next, the MCC-McP-Au^0/Au^I suspension was transferred to a centrifuge vial and washed with HCl (1 x 15 mL, 0.1 M), deionized H_2O (3 x 15 mL) and acetone (3 x 15 mL) by repeated resuspension and centrifugation. MCC-McP-Au^0/Au^I was dried under vacuum to give a light brown powder (305 mg).

MCC-McP-Au^0 synthetic procedure.

MCC-TPSi-Au^0/Au^I (200 mg) was slowly added to a vigorously stirred solution of $NaBH_4$ (40 mg, 1.05 mmol) in distilled H_2O (7 mL) at 0°C. After 2h of stirring at room temperature, the MCC-McP-Au^0 suspension was transferred to a centrifuge vial and washed with deionized H_2O (3 x 15 mL) and acetone (3 x 15 mL) by repeated resuspension and centrifugation. Next, the MCC-McP-Au^0 was dried under vacuum to give a dark brown powder (189 mg). The Au content determined by elemental analysis was 4.98 wt%.

MCC-AmP synthetic procedure.

To a suspension of Avicel PH-101 (500 mg, 3.08 mmol, 1 equiv) and L-(+)-tartaric acid (73 mg, 0.46 mmol, 5 mol%), in dry toluene (20 mL), was added 3-(Trimethoxysilyl)-propylamine (1.64 g, 9.2 mmol, 3 equiv). The reaction was stirred 48 hours at 80°C under nitrogen. Next, the solution was cooled at room temperature, transferred in an extraction thimble and washed by Soxhlet extraction with acetone for 18 hours. MCC-AmP was dried under vacuum to give a white powder (650 mg).

MCC-AmP- Au^0/Au^I synthetic procedure.

To a suspension of MCC-AmP (200 mg) in distilled H_2O (6 mL) was added, under vigorous stirring, a solution of $HAuCl_3$*$3H_2O$ (166 mg, 0.42 mmol) in distilled H_2O (4 mL, pH 9 adjusted with LiOH 0.5 M). The reaction was stirred for 24 hours at room temperature. Next, the MCC-AmP- Au^0/Au^I suspension was transferred to a centrifuge vial and washed with deionized H_2O (3 x 15 mL) and acetone (3 x 15 mL) by repeated resuspension and centrifugation. MCC-AmP-Au^0/Au^I was dried under vacuum to give a dark green powder (200 mg).

MCC-AmP-Au^0 synthetic procedure.

To a suspension of MCC-AmP- Au^0/Au^I (200 mg) in distilled H_2O (5 mL) was added dropwise, under vigorous stirring, a solution of $NaBH_4$ (47 mg, 1.24 mmol) in distilled H_2O (3 mL) and the reaction was continued to stir for 2 hours at room temperature. Next, the MCC-AmP-Au^0 suspension was transferred to a centrifuge vial and washed with deionized H_2O (3 x 15 mL) and acetone (3 x 15 mL) by repeated resuspension and centrifugation. MCC-AmP-Au^0 was dried under vacuum to give a black powder (155 mg). The Au content determined by elemental analysis was 16.34 wt%.

General procedure for the MCC-McP-Au^0-catalyzed cycloisomerization of alkynoic acids 2.

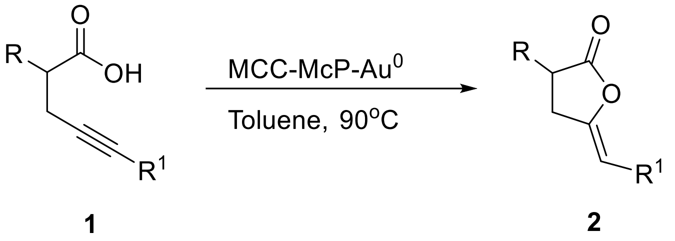

Cycloisomerization of 1a to 2a (R = R' = H). 5-methylenedihydrofuran-2(3H)-one 2a:[5] In a micro-wave vial were added MCC-McP-Au^0 (5 mg, 0.32 mol% Au) toluene (1 mL) and alkynoic acid **1a** (39 mg, 0.4 mmol). The vial was capped and flushed with nitrogen. The reaction was stirred at 90°C for 23 h. The crude reaction mixture was directly loaded on a silica-gel column and chromatographed (diethyl ether/ hexane 4:1 to 1:1) affording 38 mg (99%) of the corresponding product **2a** as a colorless oil.

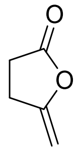

2a

5-methylenedihydrofuran-2(3H)-one 2a: 5 mg of MCC-McP-Au^0 (0.32 mol% Au) was used with a reaction time of 23 h. Yield: 38 mg, (99%) of a colorless oil; ^{1}H NMR (500 MHz, $CDCl_3$) δ 4.73 (dd, *J* = 4.3, 2.1 Hz, 1H), 4.31 (dd, *J* = 3.7, 1.8 Hz, 1H), 2.96 – 2.80 (m, 2H), 2.72 – 2.62 (m, 2H). ^{13}C NMR (126 MHz, $CDCl_3$) δ 175.0, 155.7, 88.9, 28.1, 25.2.

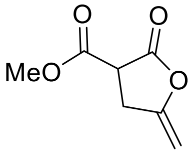

2b

Methyl 5-methylene-2-oxotetrahydrofuran-3-carboxylate 2b: 5 mg of MCC-McP-Au^0 (0.32 mol% Au) was used with a reaction time of 30 h. Yield: 60 mg, (98%) of a colorless oil; ^{1}H NMR (500 MHz, $CDCl_3$) δ 4.82 (dd, *J* = 4.5, 2.4 Hz, 1H), 4.41 (dt, *J* = 3.1, 1.7 Hz, 1H), 3.83 (s, 3H), 3.80 – 3.72 (m, 1H), 3.31 (ddt, *J* = 16.6, 7.5, 2.1 Hz, 1H), 3.10 (ddt, *J* = 16.6, 10.4, 1.6 Hz, 1H). ^{13}C NMR (126 MHz, $CDCl_3$) δ 169.6, 167.4, 153.2, 90.1, 53.5, 46.4, 29.5. **HRMS (ESI):** calculated for $C_7H_9O_4$ $[M+H]^+$157.0495; found 157.0496.

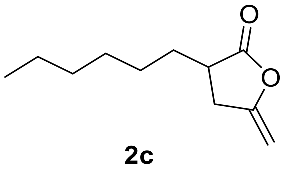

2c

3-hexyl-5-methylenedihydrofuran-2(3H)-one 2c: 15 mg of MCC-McP-Au^0 (0.96 mol% Au) was used with a reaction time of 23 h. Yield: 72 mg, (99%) as a colorless oil; ^{1}H NMR (500 MHz, $CDCl_3$) δ 4.71 (dd, *J* = 4.1, 2.2 Hz, 1H), 4.29 (dd, *J* = 3.8, 2.0 Hz, 1H), 2.99 (ddt, *J* = 16.1, 9.7, 1.5 Hz, 1H), 2.84 – 2.67 (m, 1H), 2.54 (ddt, *J* = 16.1, 7.8, 2.2 Hz, 1H), 1.97 – 1.78 (m, 1H), 1.60 – 1.44 (m, 1H), 1.44 – 1.24 (m, 8H), 0.87 (t, *J* = 6.8 Hz, 3H). ^{13}C NMR (126 MHz, $CDCl_3$) δ 177.4, 154.7, 88.8, 40.0, 31.7, 30.9, 29.0, 27.0, 22.7, 14.2. **HRMS (ESI):** calculated for $C_{11}H_{19}O_2$ $[M+H]^+$183.1380; found 183.1380.

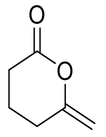

2d

5-methylenetetrahydro-2H-pyran-2-one 2d: 30 mg of MCC-McP-Au^0 (1.92 mol% Au) was used with a reaction time of 23 h. Yield: 44 mg, (99%) as a colorless oil; ^{1}H NMR (500 MHz, $CDCl_3$) δ 4.72 – 4.52 (m, 1H), 4.27 (dd, *J* = 2.6, 1.2 Hz, 1H), 2.61 (t, *J* = 6.8 Hz, 2H), 2.56 – 2.39 (m, 2H), 1.97 – 1.75 (m, 2H). ^{13}C NMR (126 MHz, $CDCl_3$) δ 168.2, 155.4, 93.8, 30.3, 26.8, 18.6. **HRMS (ESI):** calculated for $C_6H_9O_2$ $[M+H]^+$113.0597; found 113.0592.

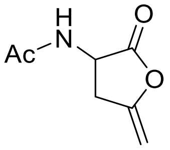

2e

N-(5-methylene-2-oxotetrahydrofuran-3-yl)acetamide 2e: 30 mg of MCC-McP-Au^0 (1.92 mol% Au) was used with a reaction time of 22 h. Yield: 56 mg, (90%) as a white solid; ^{1}H NMR (500 MHz, $CDCl_3$) δ 6.80 (d, *J* = 5.9 Hz, 1H), 4.78 (t, *J* = 2.7 Hz, 1H), 4.59 (td, *J* = 9.8, 7.1 Hz, 1H), 4.39 (t, *J* = 2.4 Hz, 1H), 3.34 – 3.13 (m, 1H), 2.95 – 2.78 (m, 1H), 2.03 (s, 3H). ^{13}C NMR (126 MHz, $CDCl_3$) δ 173.4, 170.9, 152.5, 90.5, 49.6, 32.9, 22.7. **HRMS (ESI):** calculated for $C_7H_{10}NO_3$ $[M+H]^+$156.0655; found 156.0651.

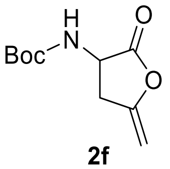

2f

Tert-butyl (5-methylene-2-oxotetrahydrofuran-3-yl) carbamate 2f: 30 mg of MCC-McP-Au^0 (1.92 mol% Au) was used with a reaction time of 27 h. Yield: 51 mg, (72%) as a white solid; ^{1}H NMR (500 MHz, $CDCl_3$) δ 5.21 (s, 1H), 4.80 (s, 1H), 4.62 – 4.26 (m, 2H), 3.26 (dd, *J* = 15.4, 9.7 Hz, 1H), 3.06 – 2.81 (m, 1H), 1.45 (s, 9H). ^{13}C NMR (126 MHz, $CDCl_3$) δ 173.3, 155.4, 152.3, 90.6, 81.1, 50.6, 33.6, 28.3. **HRMS (ESI):** calculated for $C_{10}H_{16}NO_4$ $[M+H]^+$214.1074; found 214.1071.

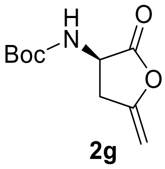

2g

Tert-butyl (R)-(5-methylene-2-oxotetrahydrofuran-3-yl) carbamate 2g: 60 mg of MCC-McP-Au^0 (3.84 mol% Au) was used with a reaction time of 23 h. Yield: 74 mg, (86%) as a white solid; ^{1}H NMR (500 MHz, $CDCl_3$) δ 5.21 (s, 1H), 4.80 (s, 1H), 4.62 – 4.26 (m, 2H), 3.26 (dd, *J* = 15.4, 9.7 Hz, 1H), 3.06 – 2.81 (m, 1H), 1.45 (s, 9H). ^{13}C NMR (126 MHz, $CDCl_3$) δ 173.3, 155.4, 152.3, 90.6, 81.1, 50.6, 33.6, 28.3. $[\alpha]_D^{25}$=+61.1 (c=1.0, $CHCl_3$). **HRMS (ESI):** calculated for $C_{10}H_{16}NO_4$ $[M+H]^+$214.1074; found 214.1069.

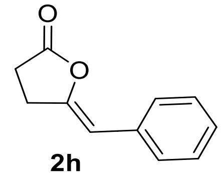

2h

(*Z*)-5-(2-phenylethylidene) dihydrofuran-2(3H)-one 2h: 30 mg of MCC-McP-Au^0 (1.92 mol% Au) was used with a reaction time of 46 h. Yield: 56 mg, (80%) as a white solid; ^{1}H NMR (500 MHz, $CDCl_3$) δ 7.58 (d, *J* = 7.6 Hz, 2H), 7.35 (t, *J* = 7.7 Hz, 2H), 7.23 (t, *J* = 7.3 Hz, 1H), 5.58 (s, 1H), 3.24 – 2.98 (m, 2H), 2.74 (dd, *J* = 9.5, 7.6 Hz, 2H). ^{13}C NMR (126 MHz, $CDCl_3$) δ 175.1, 148.2, 134.0, 128.6, 128.4, 126.9, 105.1, 27.1, 26.5. **HRMS (ESI):** calculated for $C_{11}H_{11}O_2$ $[M+H]^+$175.0754; found 175.0754.

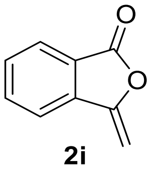

2i

(3-methyleneisobenzofuran-1(3H)-one 2i: 60 mg of MCC-McP-Au0 (3.84 mol% Au) was used with a reaction time of 18 h. Yield: 44 mg, (75%) as a white solid; ^{1}H NMR (500 MHz, $CDCl_3$) δ 7.90 (dd, *J* = 7.7, 0.8 Hz, 1H), 7.72 (dd, *J* = 4.1, 0.6 Hz, 2H), 7.66 – 7.51 (m, 1H), 5.31 – 5.18 (m, 2H). ^{13}C NMR (126 MHz, $CDCl_3$) δ 167.0, 151.9, 139.1, 134.6, 130.6, 125.40 (s), 125.21 (s), 120.7, 91. **HRMS (ESI):** calculated for $C_9H_7O_2$ $[M+H]^+$147.0441; found 147.0428.

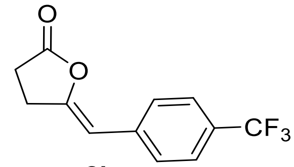

2j

(*Z*)-5-(2-(4-(trifluoromethyl) phenyl)ethylidene)dihydrofuran-2(3H)-one 2j: 30 mg of MCC-AmP-Au^0 (6.2 mol% Au) was used with a reaction time of 23 h. Yield: 42 mg, (73%) as a white solid; ^{1}H NMR (500 MHz, $CDCl_3$) δ 7.64 (d, *J* = 8.2 Hz, 2H), 7.56 (d, *J* = 8.3 Hz, 2H), 5.59 (s, 1H), 3.18 – 3.00 (m, 2H), 2.84 – 2.66 (m, 2H). ^{13}C NMR (126 MHz, $CDCl_3$) δ 174.6, 150.3, 128.5 (d, *J* = 8.7 Hz), 128.3, 125.43 (q, *J* = 3.8 Hz), 123.2, 103.8, 26.8, 26.5. ^{19}F NMR (471 MHz, $CDCl_3$) δ -62.4. **HRMS (ESI):** calculated for $C_{12}H_{10}F_3O_2$ $[M+H]^+$ 243.0627; found 243.0616.

Recycling procedure of MCC-McP-Au^0 catalyst for the cycloisomerization of alkynoc acid.

In a microwave vial, were sequentially added MCC-McP-Au^0 (20 mg, 1.28 mol% Au), toluene (1 mL) pent-4-ynoic acid **1a** (0.4 mmol, 1 equiv) and 1,4-dinitrobenzene as internal standard (16.8 mg, 0.1 mmol). The vial was capped and flushed with nitrogen. The reaction was stirred at 90°C and monitored by ^{1}H-NMR. Next, dry toluene was added to the vial and the reaction mixture was centrifuged 3 times collecting the supernatant after each cycle. After the last centrifuge cycle, 1 mL of toluene was left in the vial and fresh substrate was added. The cycle was repeated.

General procedure for the MCC-McP-Au^{0-}catalyzed cycloisomerization of allenynamides 3.

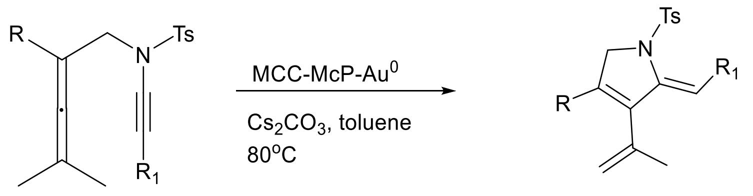

3 **4**

In a microwave vial, were added MCC-McP-Au^0 (54 mg, 9 mol% Au), Cs_2CO_3 (65 mg, 0.195 mmol, 1.3 equiv), toluene (1 mL) and allenynamide **3** (0.15 mmol, 1 equiv). The vial was capped and flushed with nitrogen. The reaction was stirred at 80°C. Next the crude reaction mixture was directly loaded on a silica-gel column and chromatography (diethylether/ hexane) afforded the corresponding product **4**.

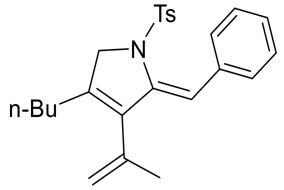

4a

(*Z*)-2-benzylidene-4-butyl-3-(prop-1-en-2-yl)-1-tosyl-2,5-dihydro-1H-pyrrole 4a: 57 mg, yield 93%; Colorless oil. **^{1}H NMR (500 MHz, $CDCl_3$)** δ 7.78 (d, *J* = 7.3 Hz, 2H), 7.67 (d, *J* = 8.3 Hz, 2H), 7.33 (t, *J* = 7.7 Hz, 2H), 7.23 (d, *J* = 8.0 Hz, 2H), 7.19 (t, *J* = 7.4 Hz, 1H), 6.01 (s, 1H), 5.18 (s, 1H), 4.65 (s, 1H), 4.25 (s, 2H), 2.39 (s, 3H), 1.95 (t, *J* = 7.3 Hz, 2H), 1.72 (s, 3H), 1.03 – 0.93 (m, 4H), 0.79

(t, J = 7.0 Hz, 3H) ppm; **^{13}C NMR (126 MHz, CDCl$_3$)** δ 144.0, 143.2, 139.4, 137.8, 137.2, 136.8, 133.1, 129.2, 128.2, 127.7, 126.6, 117.6, 115.0, 58.4, 30.2, 26.6, 22.7, 22.3, 21.5, 13.9; **HRMS (ESI):** calculated for $C_{25}H_{30}NO_2S$ [M+H]$^+$ 408.1992; found 408.1995.

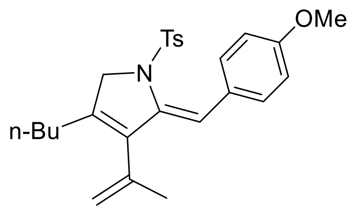

4b

(*Z*)-4-butyl-2-(4-methoxybenzylidene)-3-(prop-1-en-2-yl)-1-tosyl-2,5-dihydro-1H-pyrrole 4b: 57 mg, yield 87%; Colorless oil. **^{1}H NMR (500 MHz, CDCl$_3$)** δ 7.71 (d, J = 8.8 Hz, 2H), 7.66 (d, J = 8.2 Hz, 2H), 7.22 (d, J = 8.4 Hz, 2H), 6.86 (d, J = 8.7 Hz, 2H), 5.93 (s, 1H), 5.15 (d, J = 1.5 Hz, 1H), 4.61 (s, 1H), 4.22 (s, 2H), 3.81 (s, 3H), 2.38 (s, 3H), 1.91 (t, J = 7.0 Hz, 2H), 1.68 (s, 3H), 1.04 – 0.90 (m, 4H), 0.77 (t, J = 6.8 Hz, 3H) ppm; **^{13}C NMR (126 MHz, CDCl$_3$)** δ δ 158.4, 144.0, 141.6, 139.6, 137.5, 136.7, 133.3, 130.7, 129.4, 129.1, 128.3, 117.6, 115.1, 58.4, 55.3, 30.3, 26.6, 22.8, 22.4, 21.6, 14.0.ppm; **HRMS (ESI):** calculated for $C_{26}H_{32}NO_3S$ [M+H]$^+$ 438.2097; found 438.2112.

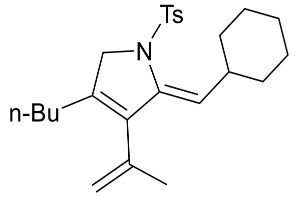

4c

(*Z*)-4-butyl-2-(cyclohexylmethylene)-3-(prop-1-en-2-yl)-1-tosyl-2,5-dihydro-1H-pyrrole 4c: 44 mg, yield 71%; Colorless oil. **^{1}H NMR (500 MHz, CDCl$_3$)** δ 7.64 (d, J = 8.2 Hz, 2H), 7.20 (d, J = 8.3 Hz, 2H), 5.05 (s, 1H), 4.87 (d, J = 10.5 Hz, 1H), 4.55 (d, J = 1.0 Hz, 1H), 4.11 (s, 2H), 2.98 (qt, J = 10.9, 3.4 Hz, 1H), 2.37 (s, 3H), 1.87 (t, J = 7.4 Hz, 4H), 1.72 – 1.65 (m, 3H), 1.57 (s, 3H), 1.39 (dt, J = 22.2, 9.6 Hz, 2H), 1.21 – 1.13 (m, 1H), 1.10 – 0.82 (m, 7H), 0.75 (t, J = 7.1 Hz, 3H) ppm; **^{13}C NMR (126 MHz, CDCl$_3$)** δ 143.7, 140.7, 138.5, 137.7, 135.1, 134.0, 129.2, 128.1, 123.9, 117.1, 58.4, 37.3, 33.6, 30.2, 26.5, 26.0, 22.6, 22.4, 21.6, 14.0; **HRMS (ESI):** calculated for $C_{25}H_{36}NO_2S$ [M+H]$^+$ 414.2461; found 414.2470.

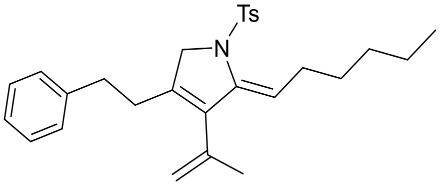

4d

(*Z*)-2-benzylidene-4-phenethyl-3-(prop-1-en-2-yl)-1-tosyl-2,5-dihydro-1H-pyrrole 4d: 57 mg, yield 85%; Colorless oil. **^{1}H NMR (500 MHz, $CDCl_3$)** Colorless oil. δ 7.67 (d, *J* = 8.2 Hz, 2H), 7.29 – 7.20 (m, 5H), 7.05 (d, *J* = 7.1 Hz, 2H), 5.14 – 5.05 (m, 2H), 4.53 (d, *J* = 1.0 Hz, 1H), 4.22 (s, 2H), 2.62 – 2.51 (m, 2H), 2.41 (s, 3H), 2.36 – 2.29 (m, 2H), 2.26 – 2.18 (m, 2H), 1.58 (d, *J* = 13.4 Hz, 3H), 1.50 – 1.42 (m, 2H), 1.39 – 1.32 (m, 4H), 0.96 – 0.90 (m, 3H) ppm; **^{13}C NMR (126 MHz, $CDCl_3$)** δ 143.8, 142.3, 141.2, 139.0, 137.5, 134.2, 133.9, 129.2, 128.6, 128.1, 126.3, 118.9, 117.3, 58.4, 34.5, 31.8, 29.9, 29.4, 28.9, 22.7, 22.5, 21.7, 14.3; **HRMS (ESI):** calculated for $C_{28}H_{36}NO_2S$ $[M+H]^+$ 450.2461; found 450.2464.

Kinetic Data

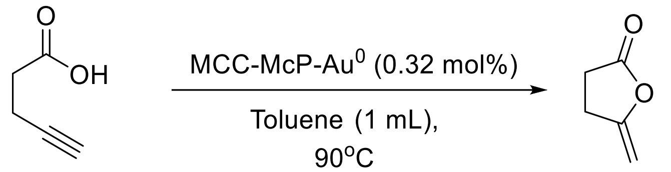

1a
(0.4 mmol)

2a

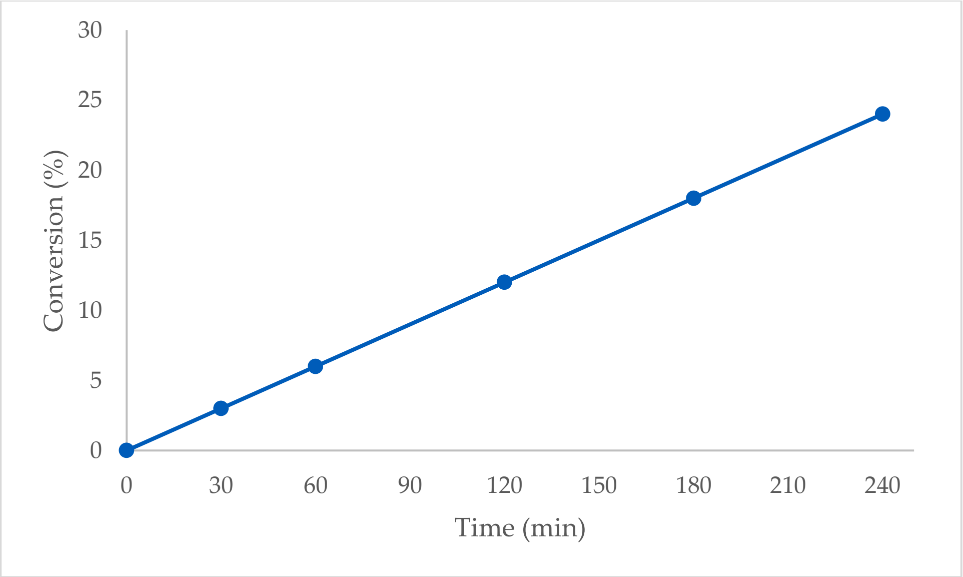

Time (min)	Conversion (%)
0	0
30	3.05
60	6.12
120	11.89
180	17.62
240	23.47

From the data in the diagram above it is clear that the reaction is zero order (at least up to 25%). Furthermore, the turnover frequency (TOF) calculated form that diagram is 19 h^{-1}. The turnover number (TOF) of the reaction is 19 (99% yield with 0.32 mol% of catalyst; see Scheme 2).

Copies of Spectra

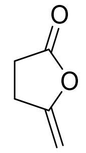

2a

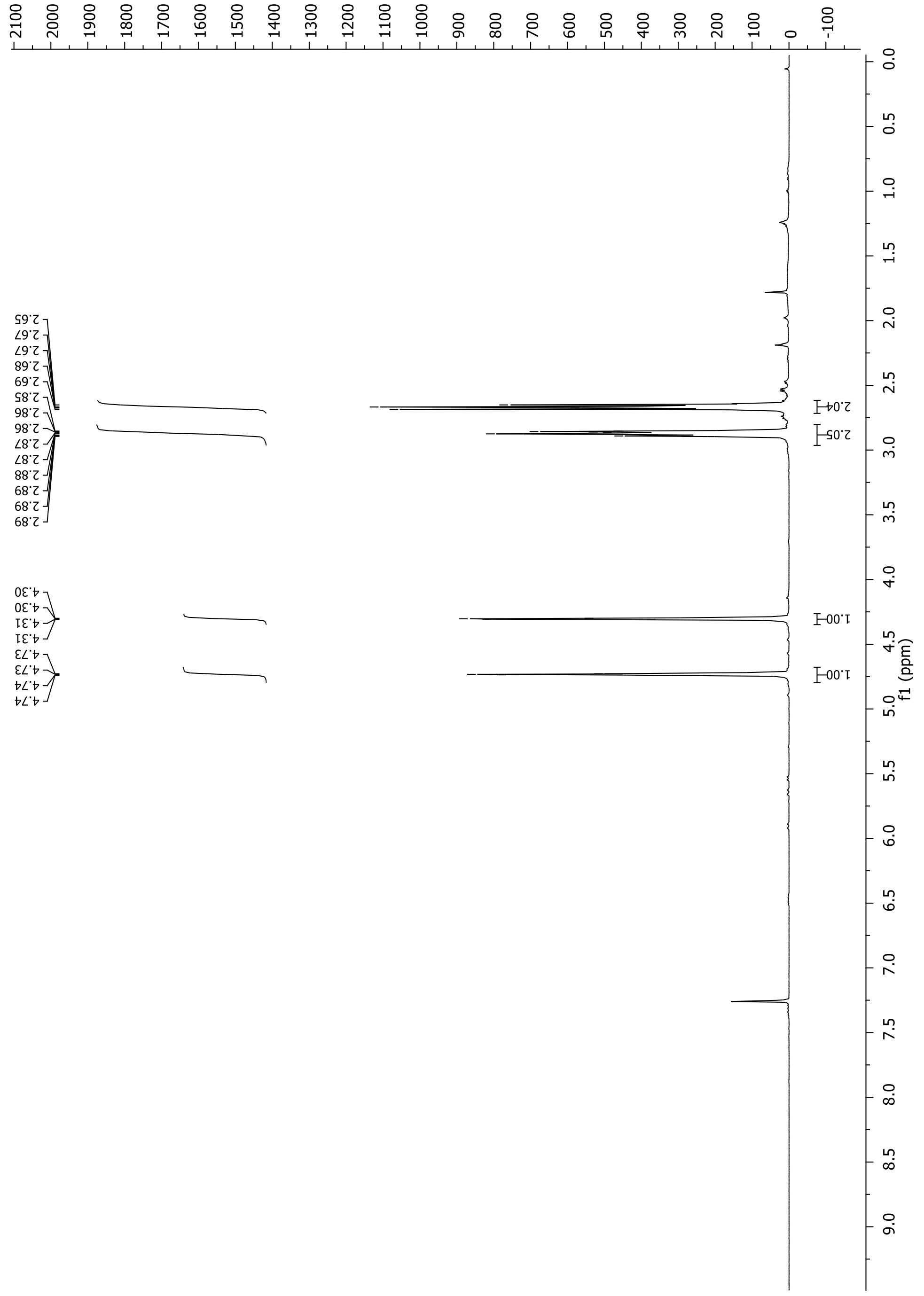

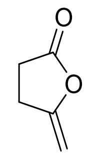

2a

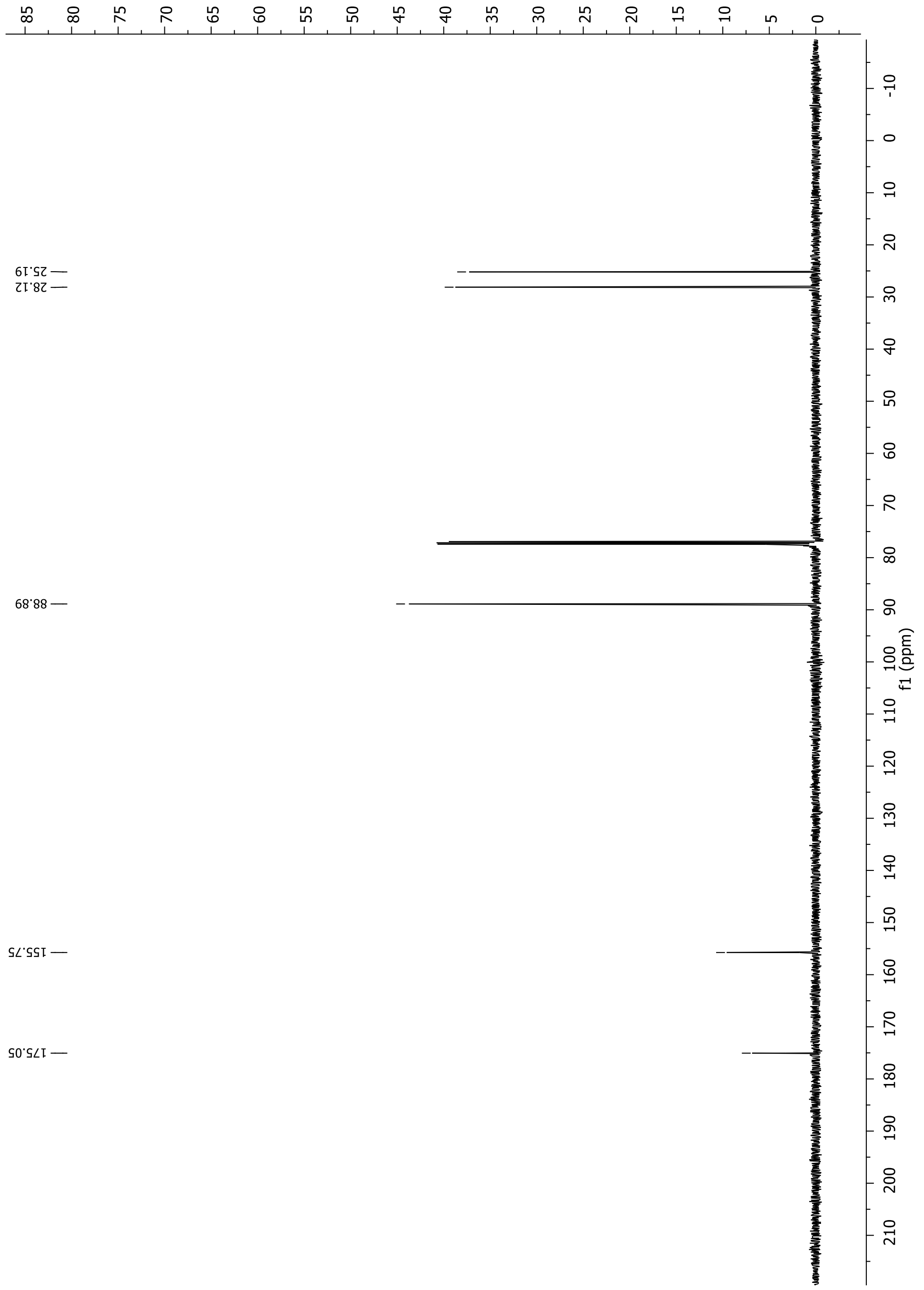

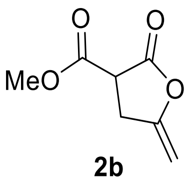

2b

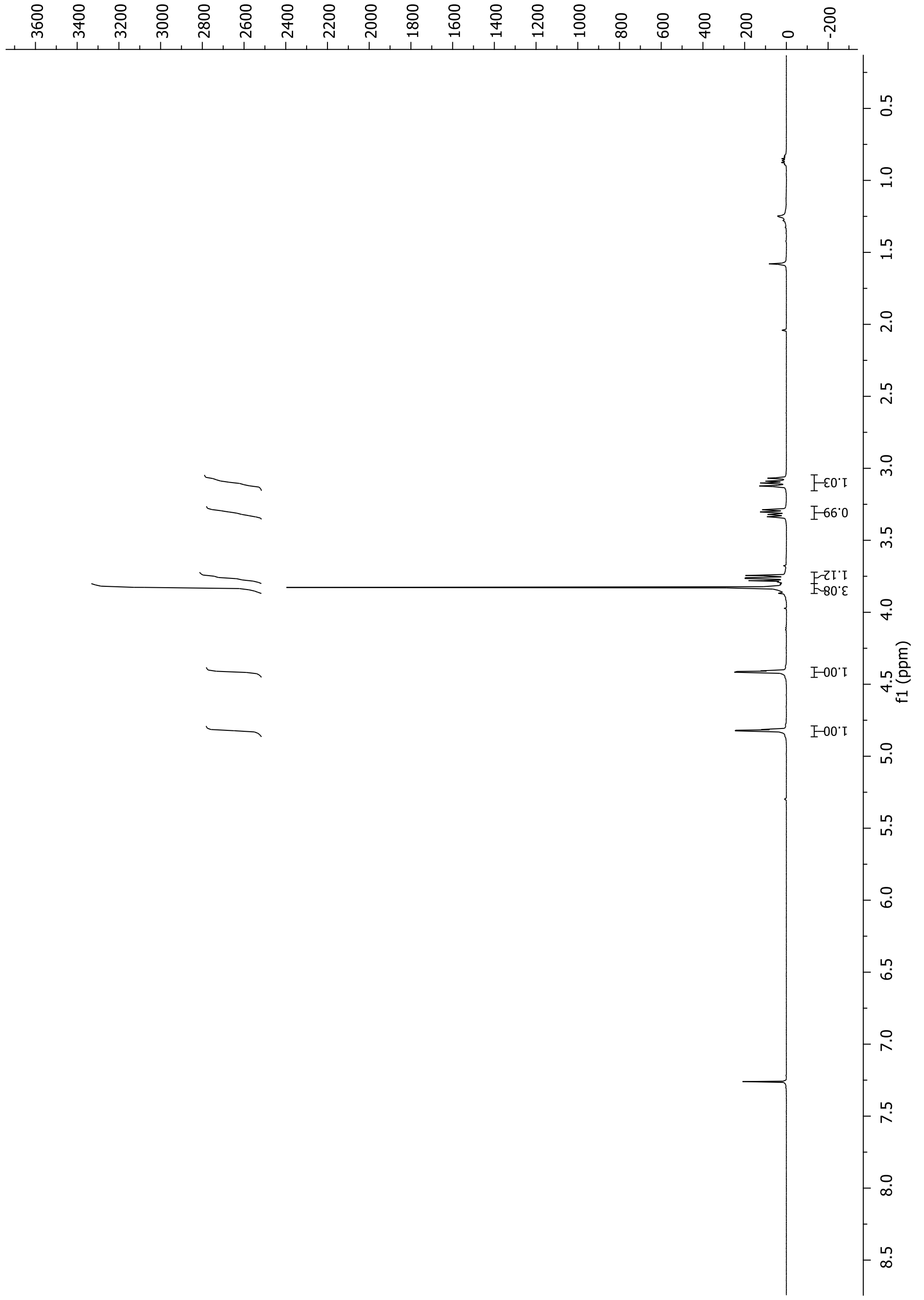

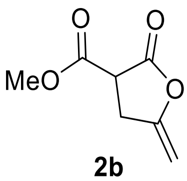

2b

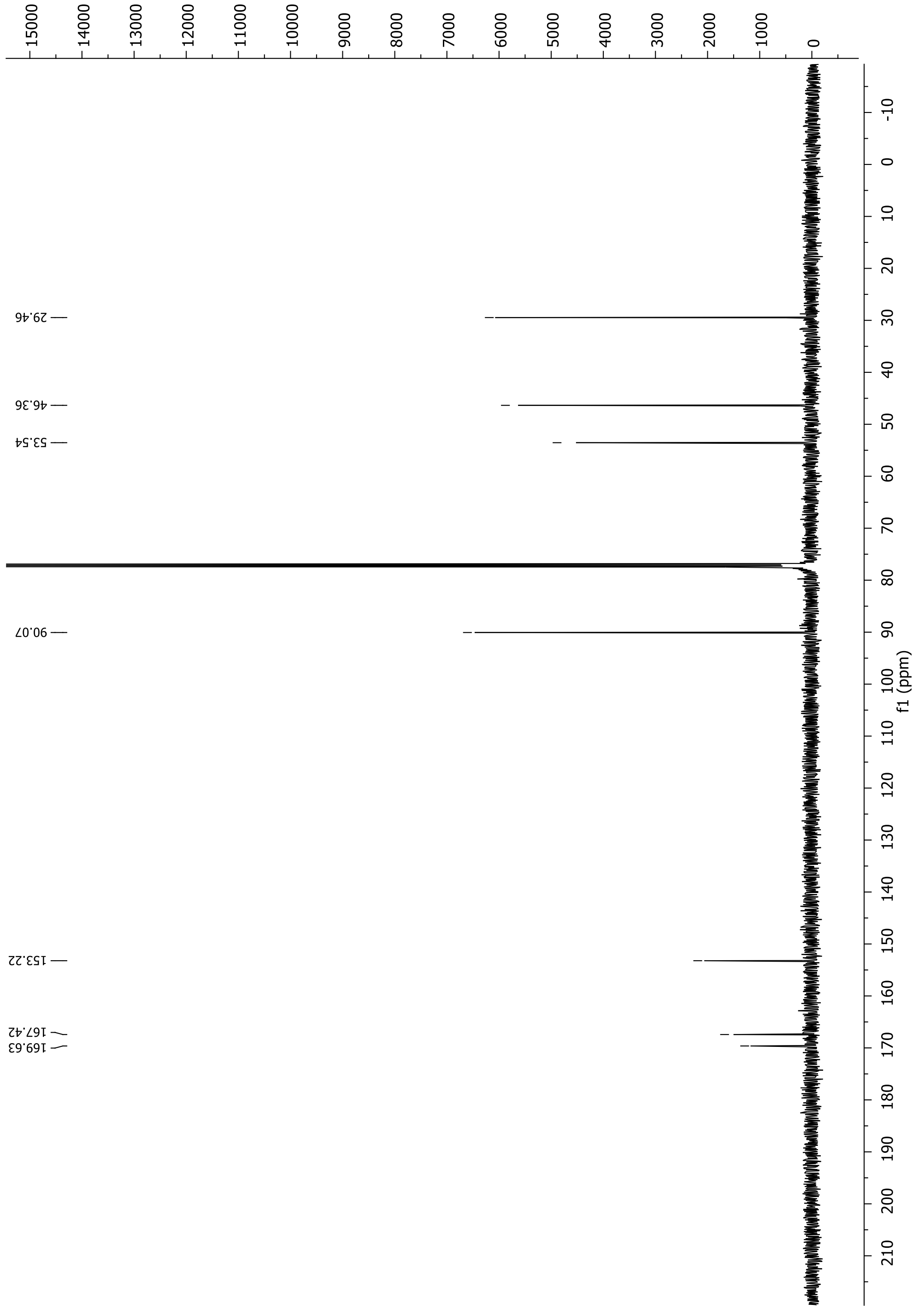

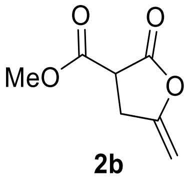

2b

Qualitative Analysis Report

**Data Filename**	LD1003.d	**Sample Name**	Unavailable
**Sample Type**	Unavailable	**Position**	Unavailable
**Instrument Name**	Unavailable	**User Name**	Unavailable
**Acq Method**		**Acquired Time**	Unavailable
**IRM Calibration Status**	Success	**DA Method**	furfural 25.03.2021.m
**Comment**	Sample information is unavailable		

Compounds

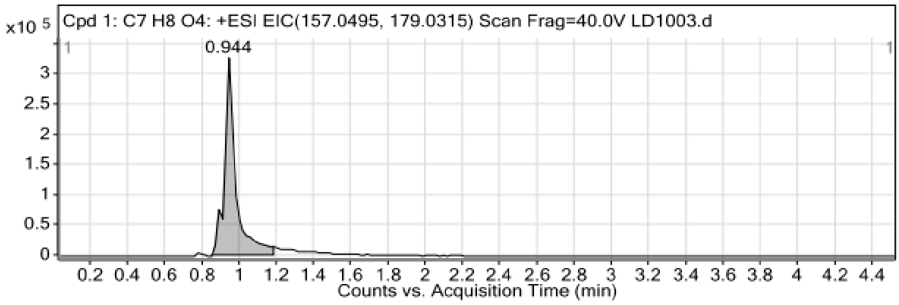

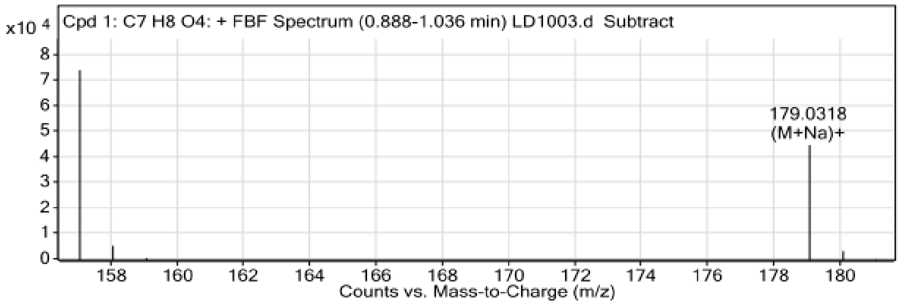

Peak List

m/z	z	Abund	Formula	Ion
157.0496	1	74276.86	C7H9O4	(M+H)+
158.053	1	5678.96	C7H9O4	(M+H)+
159.0549	1	750.81	C7H9O4	(M+H)+
179.0318	1	44859.98	C7H8NaO4	(M+Na)+
180.0355	1	3551.61	C7H8NaO4	(M+Na)+
181.0382	1	424.94	C7H8NaO4	(M+Na)+

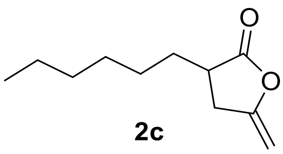

2c

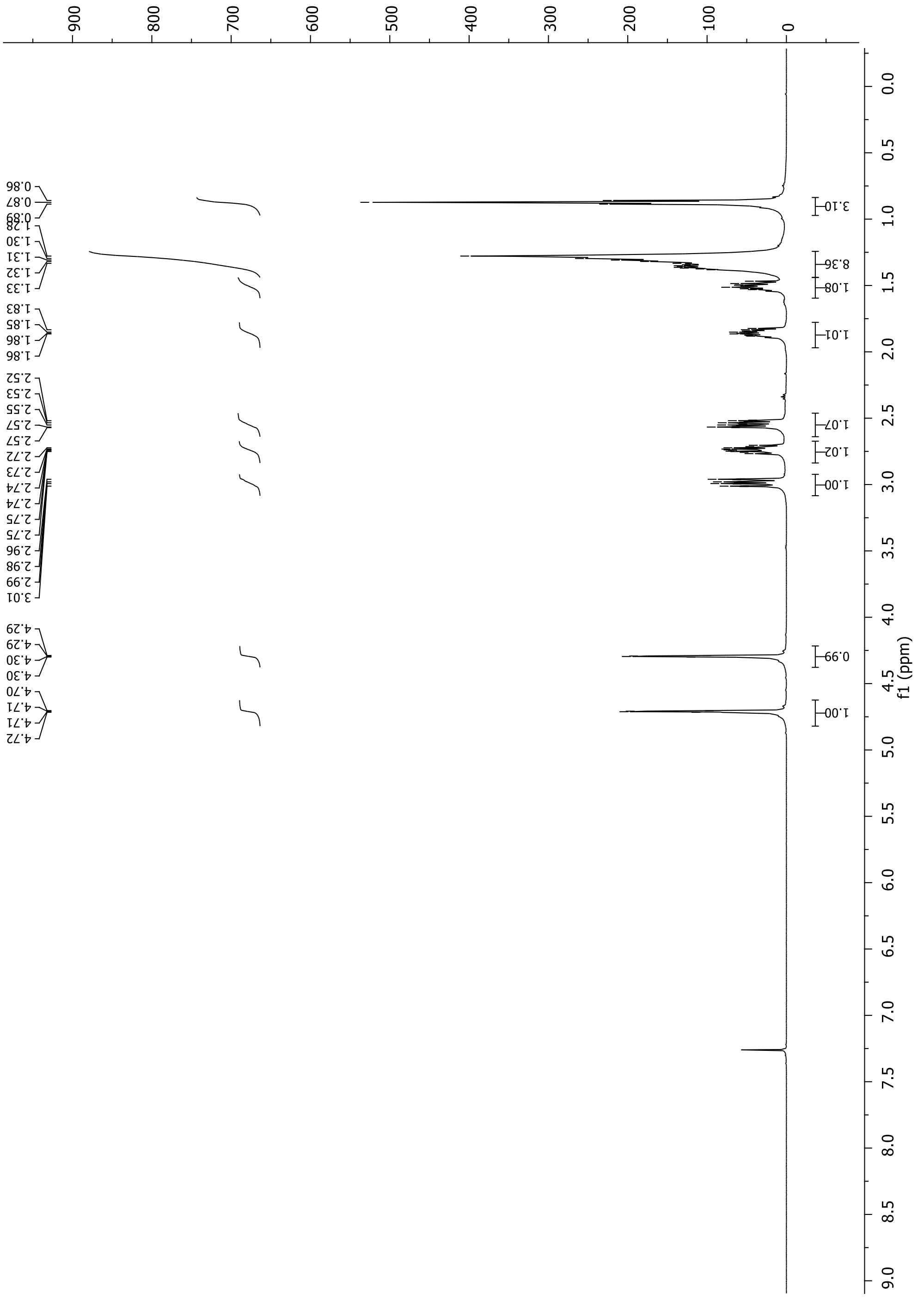

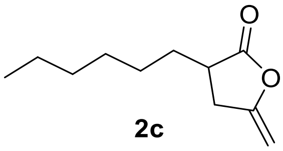
O
O

2c

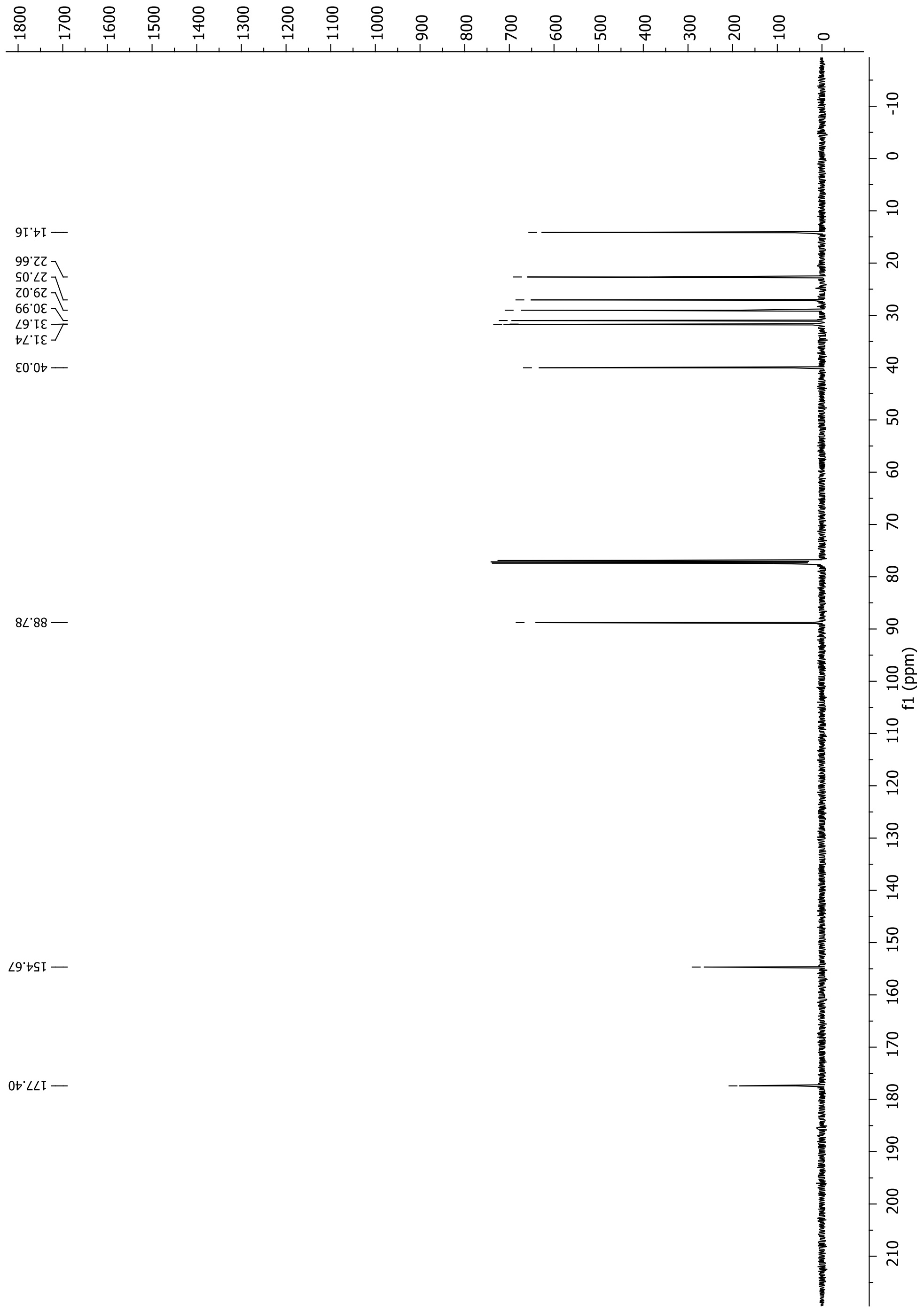
14.16
22.66
27.05
29.02
30.99
31.67
31.74
40.03
88.78
154.67
177.40
f1 (ppm)

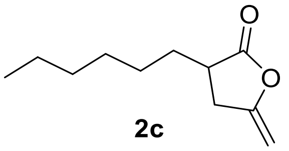

2c

Qualitative Analysis Report

**Data Filename**	LD1008.d	**Sample Name**	LD1008
**Sample Type**	Sample	**Position**	vial 1
**Instrument Name**	QTOF	**User Name**	QTOF-PC\admin
**Acq Method**	ACgroup_new.m	**Acquired Time**	2021-10-26 11:41:04
**IRM Calibration Status**	Success	**DA Method**	furfural 25.03.2021.m
**Comment**	LD1008		

**Acquisition SW**	6200 series TOF/6500 series
**Version**	Q-TOF B.05.00 (B5042.2)

Compounds

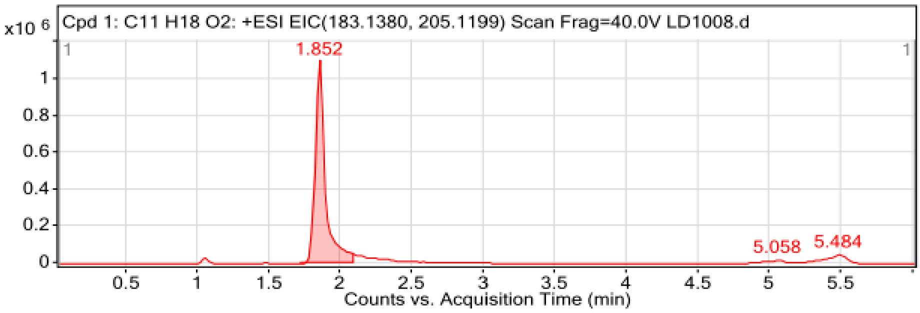

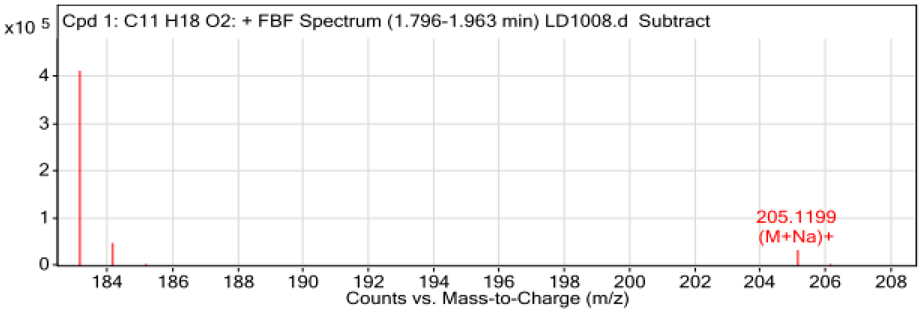

Peak List

m/z	z	Abund	Formula	Ion
183.138	1	412990.88	C11H19O2	(M+H)+
184.1412	1	50395.6	C11H19O2	(M+H)+
185.1441	1	4774.2	C11H19O2	(M+H)+
186.1453	1	390.21	C11H19O2	(M+H)+
205.1199	1	35213.46	C11H18NaO2	(M+Na)+
206.1233	1	4240.89	C11H18NaO2	(M+Na)+
207.1289	1	484.37	C11H18NaO2	(M+Na)+
208.1268	1	33.1	C11H18NaO2	(M+Na)+

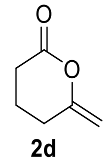

2d

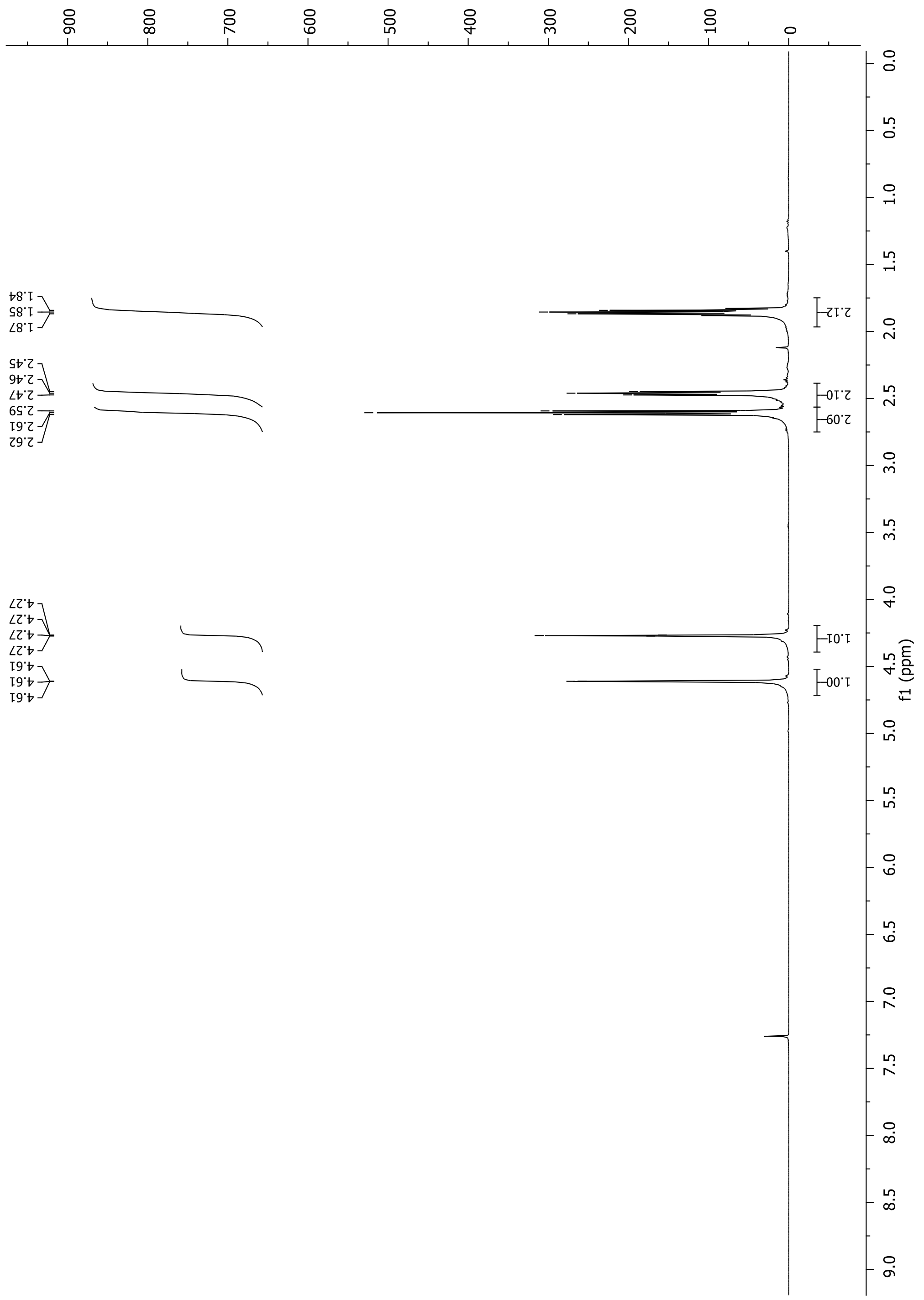
1.84
1.85
1.87
2.45
2.46
2.47
2.59
2.61
2.62
4.27
4.27
4.27
4.27
4.61
4.61
4.61
2.12
2.10
2.09
1.01
1.00
f1 (ppm)

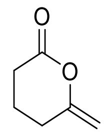

2d

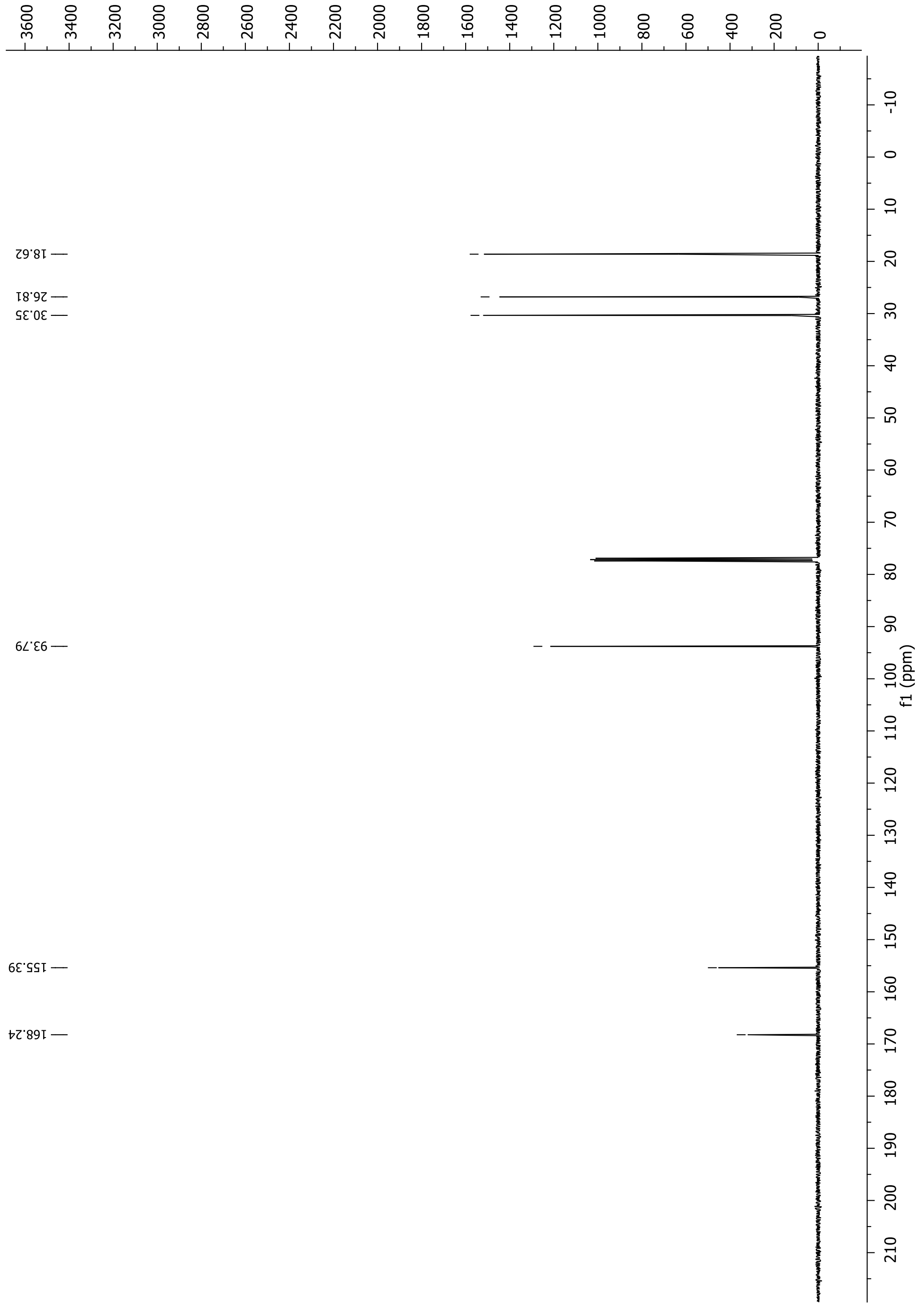

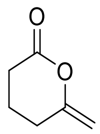

2d

Qualitative Analysis Report

**Data Filename**	LD1009.d	**Sample Name**	Unavailable
**Sample Type**	Unavailable	**Position**	Unavailable
**Instrument Name**	Unavailable	**User Name**	Unavailable
**Acq Method**		**Acquired Time**	Unavailable
**IRM Calibration Status**	Success	**DA Method**	furfural 25.03.2021.m
**Comment**	Sample information is unavailable		

Compounds

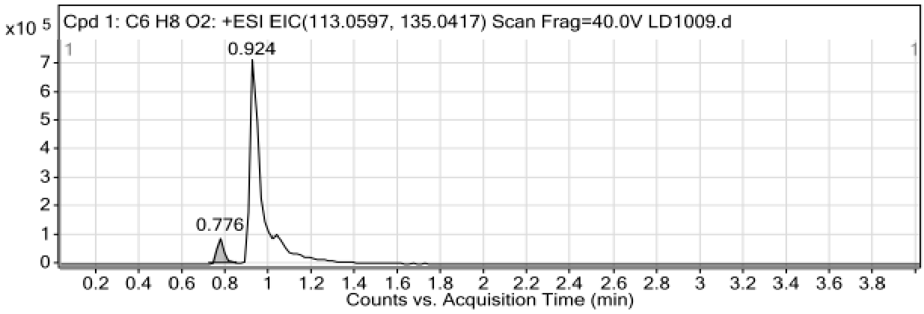

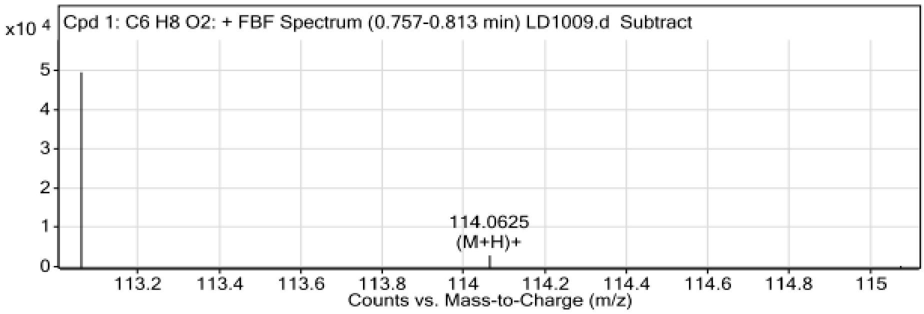

Peak List

m/z	z	Abund	Formula	Ion
113.0592	1	49842.42	C6H9O2	(M+H)+
114.0625	1	3318.83	C6H9O2	(M+H)+
115.0692	1	466.34	C6H9O2	(M+H)+

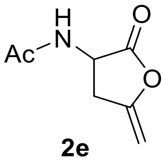

2e

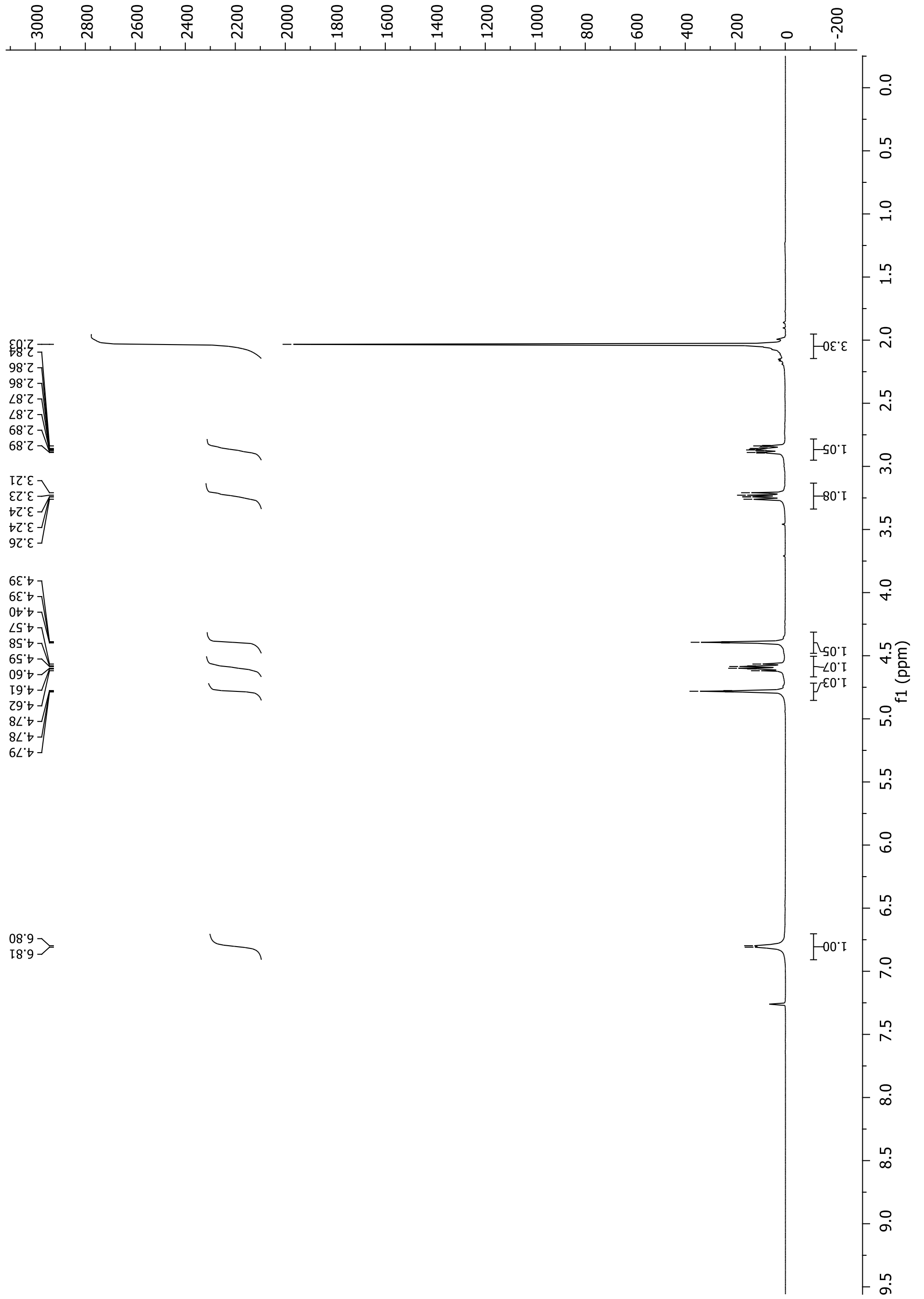
2.03
2.84
2.86
2.86
2.87
2.87
2.89
2.89
3.21
3.23
3.24
3.24
3.26
4.39
4.39
4.40
4.57
4.58
4.59
4.60
4.61
4.62
4.78
4.78
4.79
6.80
6.81
3.30
1.05
1.08
1.05
1.07
1.03
1.00
f1 (ppm)

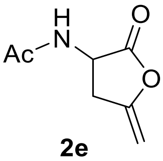

2e

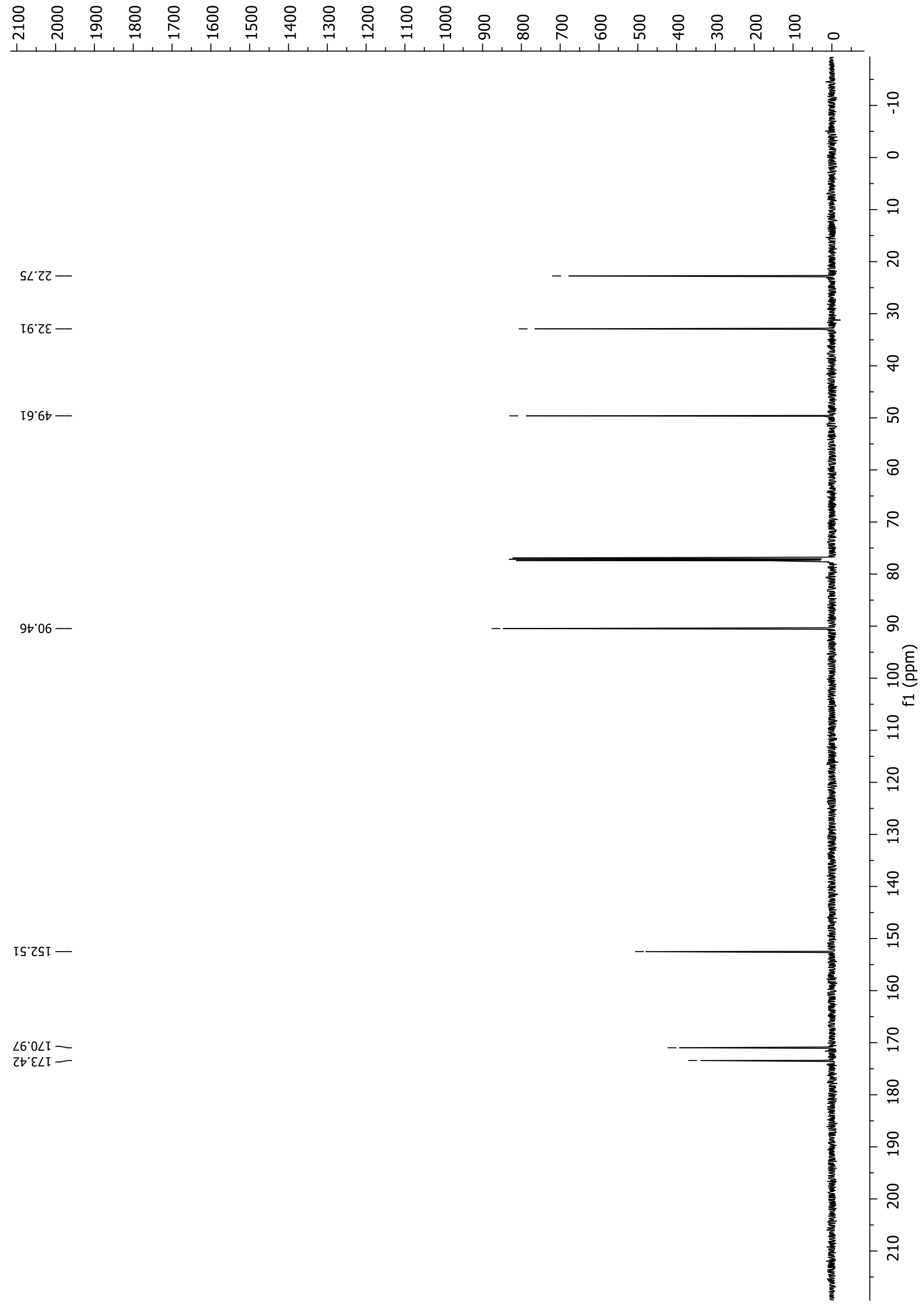

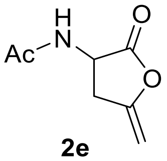

2e

Qualitative Analysis Report

**Data Filename**	LD1019.d	**Sample Name**	Unavailable
**Sample Type**	Unavailable	**Position**	Unavailable
**Instrument Name**	Unavailable	**User Name**	Unavailable
**Acq Method**		**Acquired Time**	Unavailable
**IRM Calibration Status**	Success	**DA Method**	furfural 25.03.2021.m
**Comment**	Sample information is unavailable		

Compounds

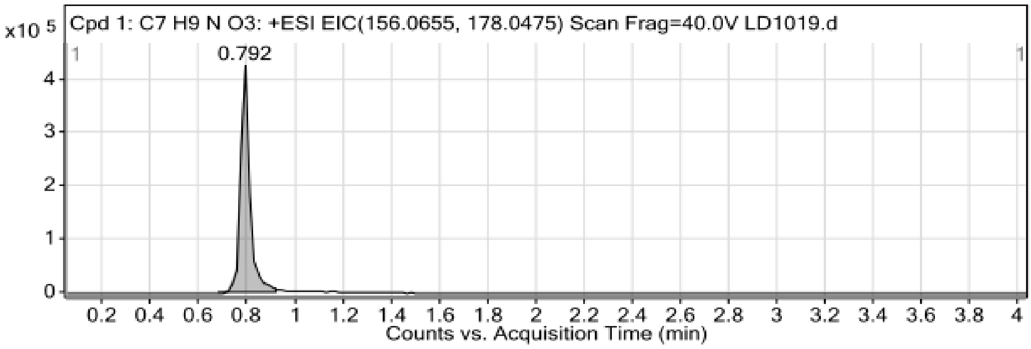

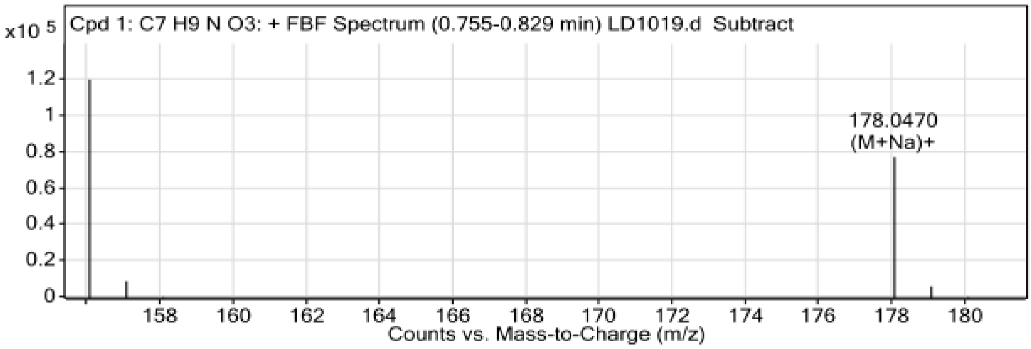

Peak List

m/z	z	Abund	Formula	Ion
156.0651	1	120375.58	C7H10NO3	(M+H)+
157.0686	1	9603.04	C7H10NO3	(M+H)+
158.0688	1	866.57	C7H10NO3	(M+H)+
178.047	1	78011.83	C7H9NNaO3	(M+Na)+
179.0503	1	6461.35	C7H9NNaO3	(M+Na)+
180.053	1	632.22	C7H9NNaO3	(M+Na)+
181.054	1	45.24	C7H9NNaO3	(M+Na)+

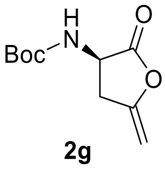
H
N
O
Boc
O

2g

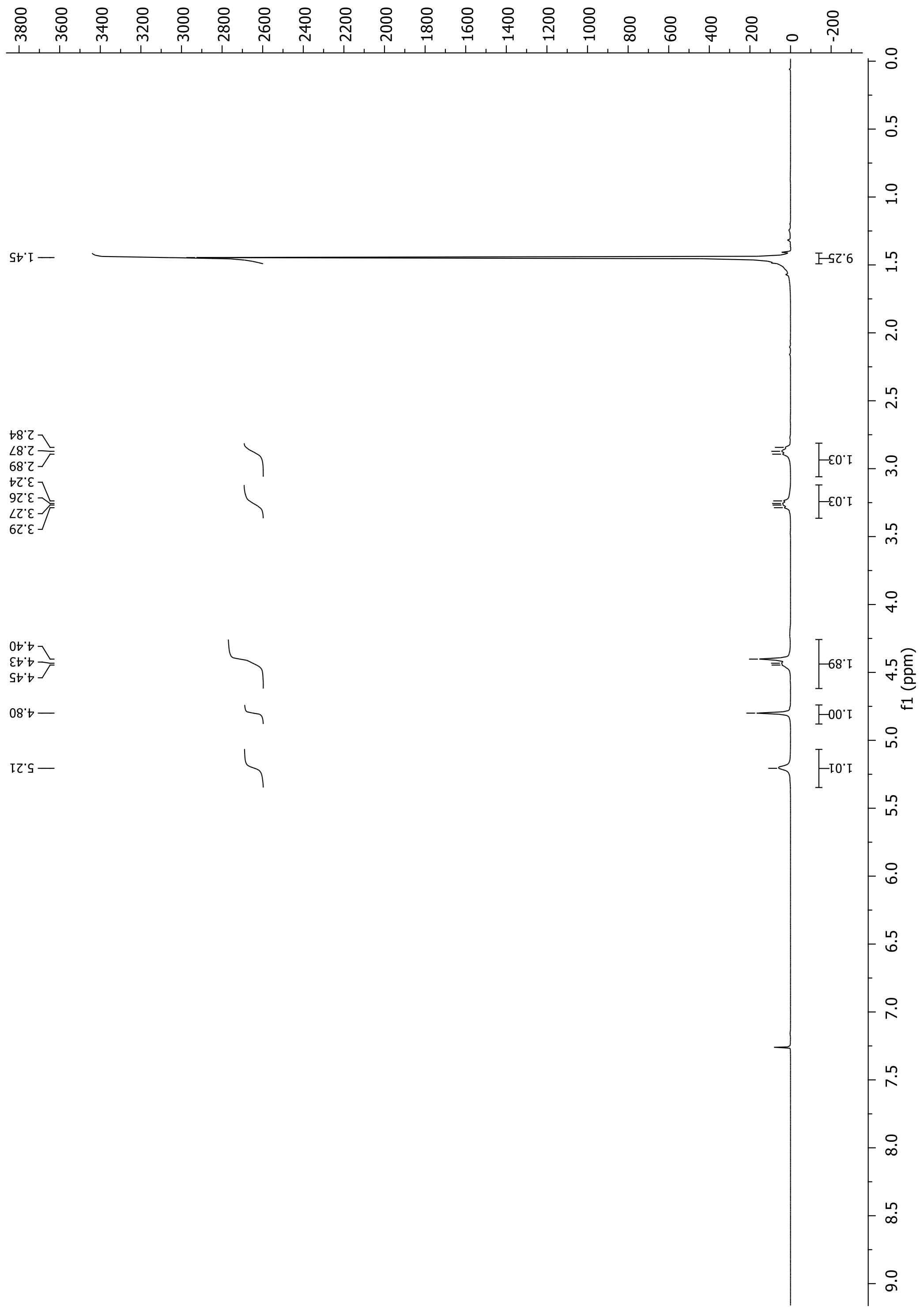
1.45
2.84
2.87
2.89
3.24
3.26
3.27
3.29
4.40
4.43
4.45
4.80
5.21
9.25
1.03
1.03
1.89
1.00
1.01
f1 (ppm)

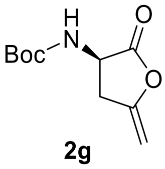

2g

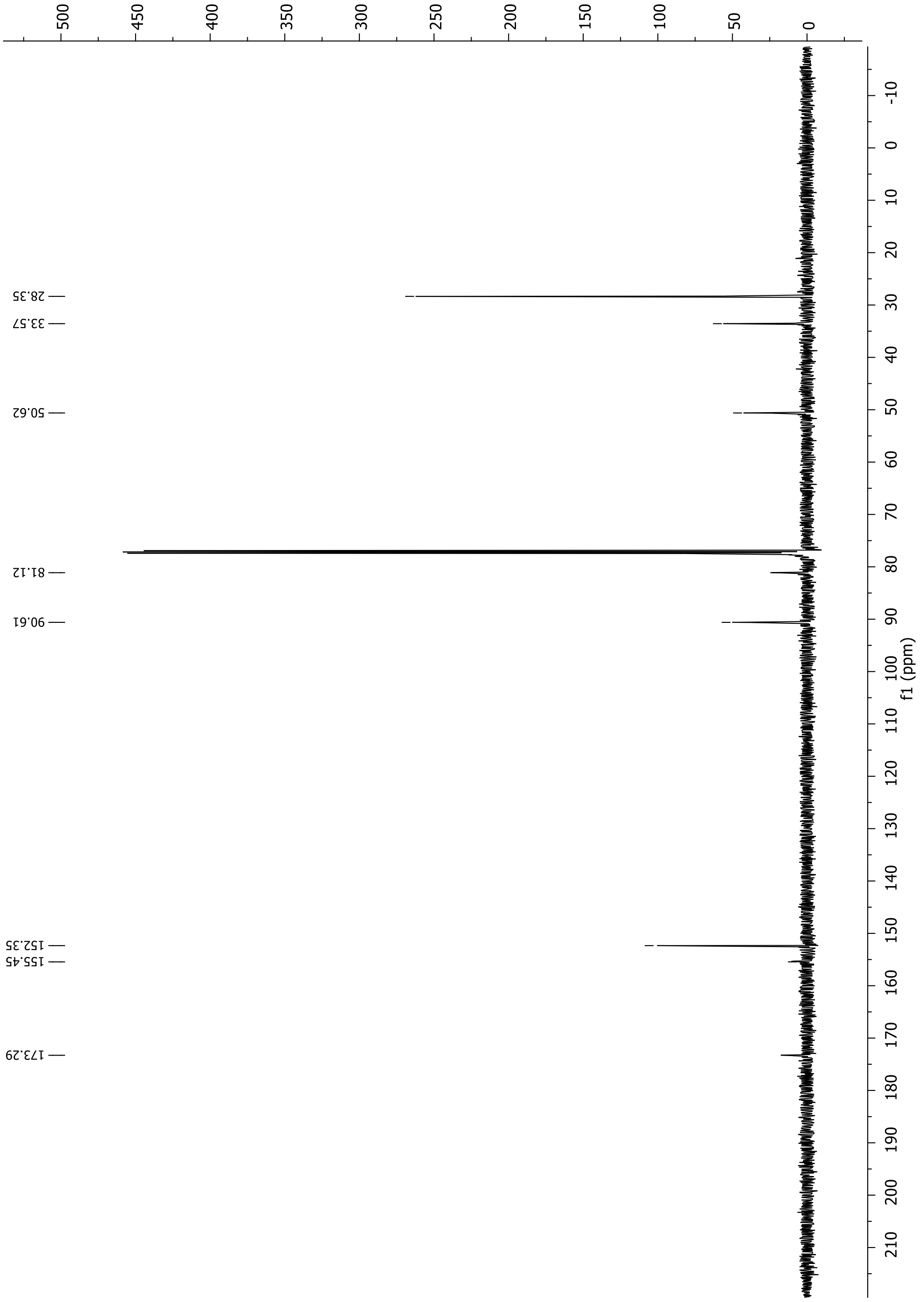

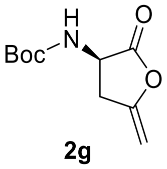

2g

Qualitative Analysis Report

**Data Filename**	LD1012.d	**Sample Name**	Unavailable
**Sample Type**	Unavailable	**Position**	Unavailable
**Instrument Name**	Unavailable	**User Name**	Unavailable
**Acq Method**		**Acquired Time**	Unavailable
**IRM Calibration Status**	Success	**DA Method**	furfural 25.03.2021.m
**Comment**	Sample information is unavailable		

Compounds

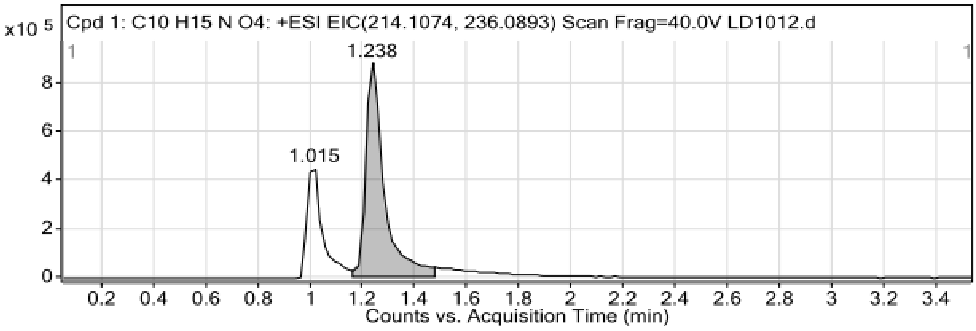

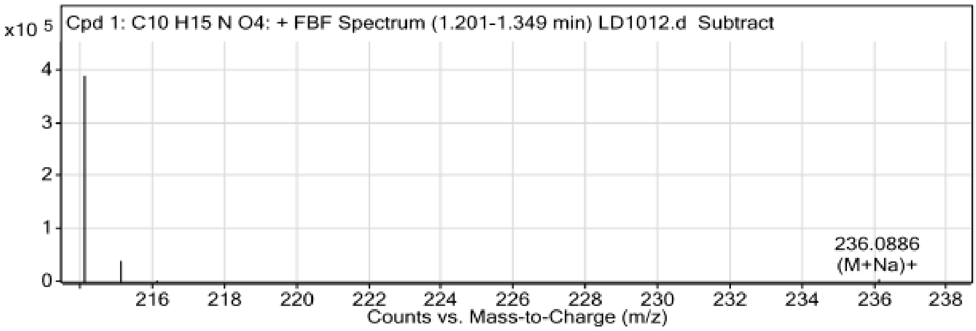

Peak List

m/z	z	Abund	Formula	Ion
214.1069	1	390669.13	C10H16NO4	(M+H)+
215.1101	1	42342.77	C10H16NO4	(M+H)+
216.1122	1	5366.55	C10H16NO4	(M+H)+
217.1083	1	1082.84	C10H16NO4	(M+H)+
236.0886	1	7780.07	C10H15NNaO4	(M+Na)+
237.0941	1	866.25	C10H15NNaO4	(M+Na)+
238.0919	1	25.59	C10H15NNaO4	(M+Na)+

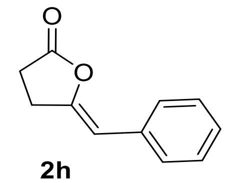

2h

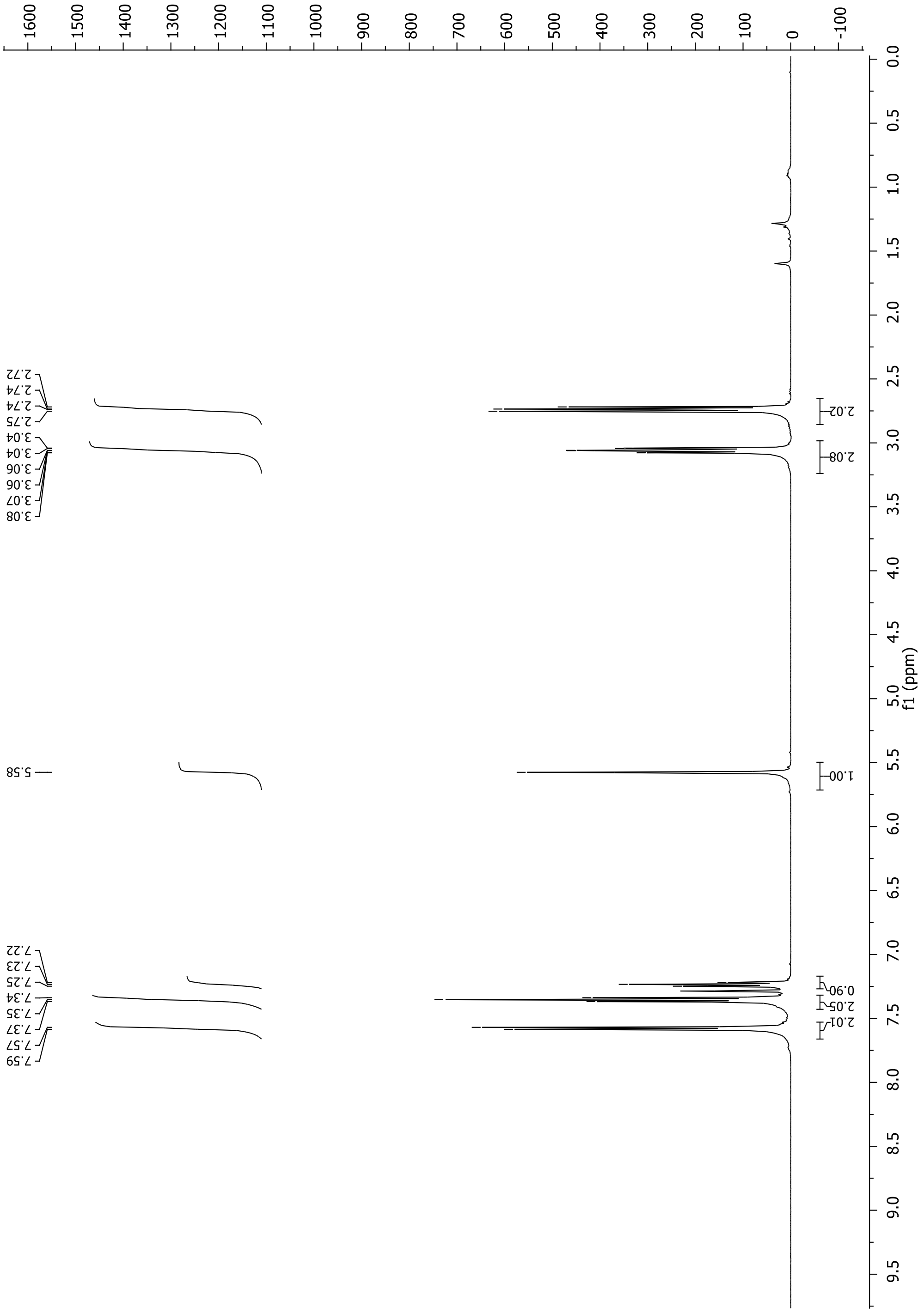

2.72
2.74
2.74
2.75
3.04
3.04
3.06
3.06
3.07
3.08
5.58
7.22
7.23
7.25
7.34
7.35
7.37
7.57
7.59
2.02
2.08
1.00
0.90
2.05
2.01
f1 (ppm)

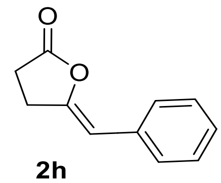

2h

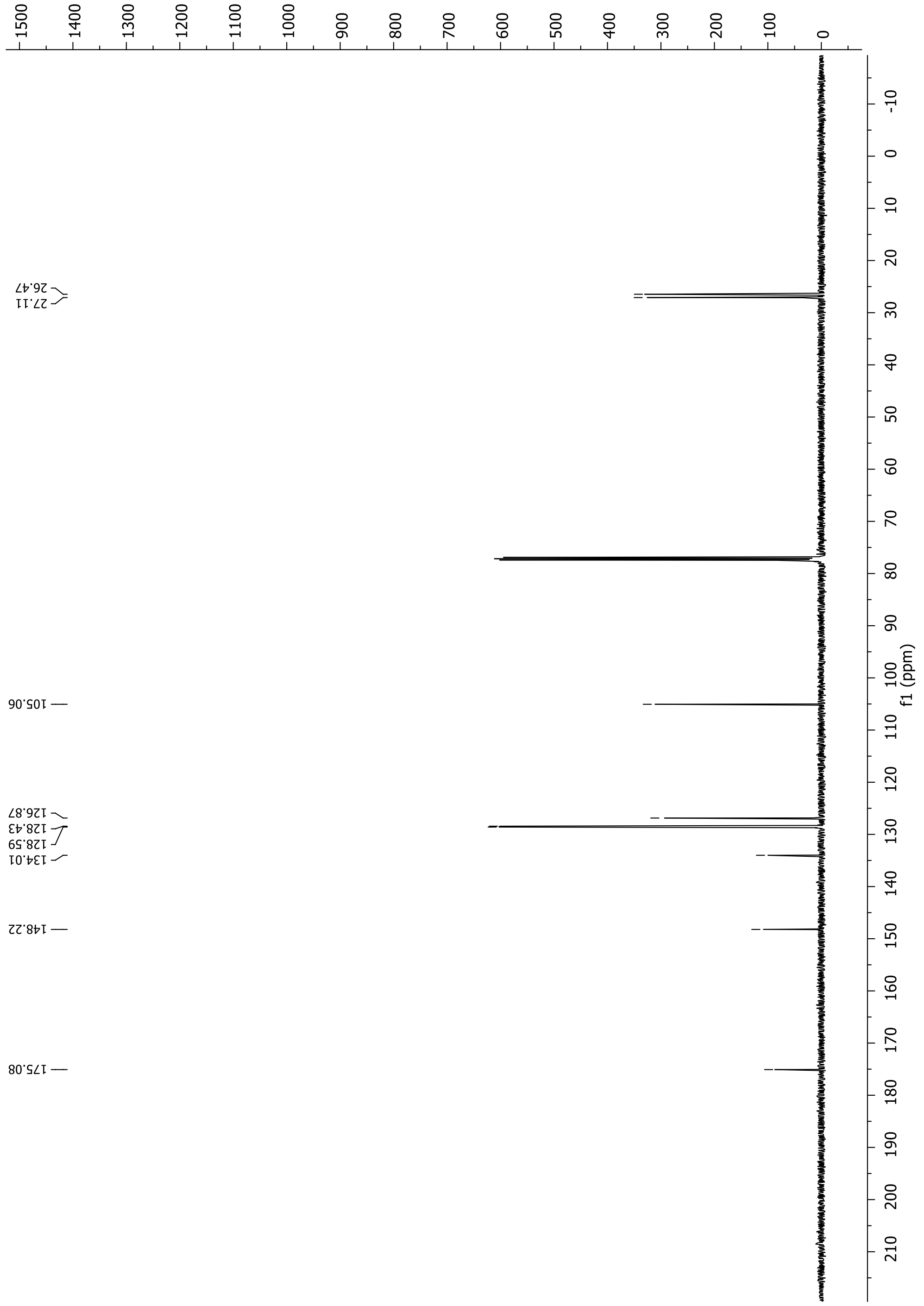

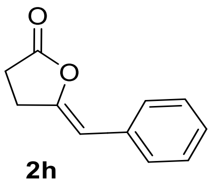

2h

Qualitative Analysis Report

**Data Filename**	LD1013.d	**Sample Name**	LD1013
**Sample Type**	Sample	**Position**	vial 1
**Instrument Name**	QTOF	**User Name**	QTOF-PC\admin
**Acq Method**	ACgroup_new.m	**Acquired Time**	2021-10-26 11:58:13
**IRM Calibration Status**	Success	**DA Method**	furfural 25.03.2021.m
**Comment**	LD1013		

**Acquisition SW**	6200 series TOF/6500 series
**Version**	Q-TOF B.05.00 (B5042.2)

Compounds

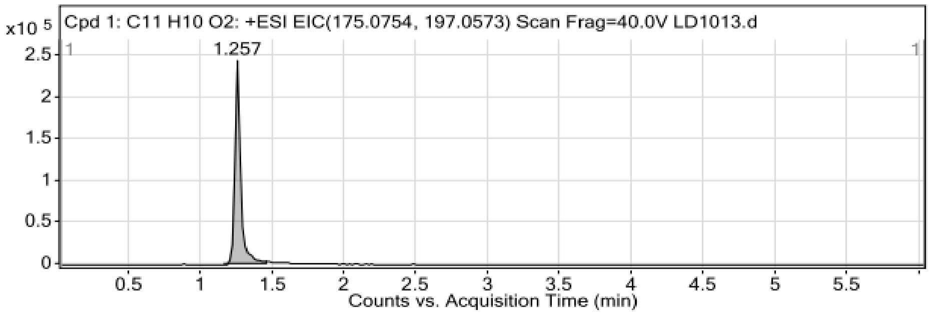

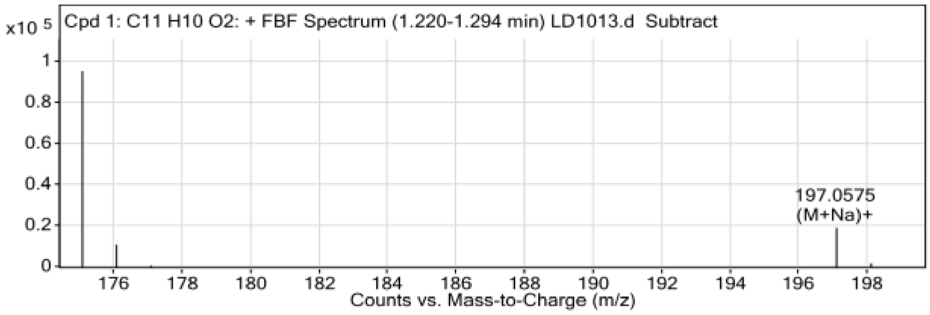

Peak List

m/z	z	Abund	Formula	Ion
175.0754	1	95714.61	C11H11O2	(M+H)+
176.0787	1	11696.18	C11H11O2	(M+H)+
177.0808	1	1080.24	C11H11O2	(M+H)+
197.0575	1	19451.15	C11H10NaO2	(M+Na)+
198.0607	1	2234.5	C11H10NaO2	(M+Na)+
199.0629	1	162.21	C11H10NaO2	(M+Na)+

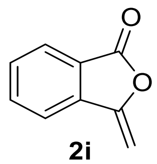

2i

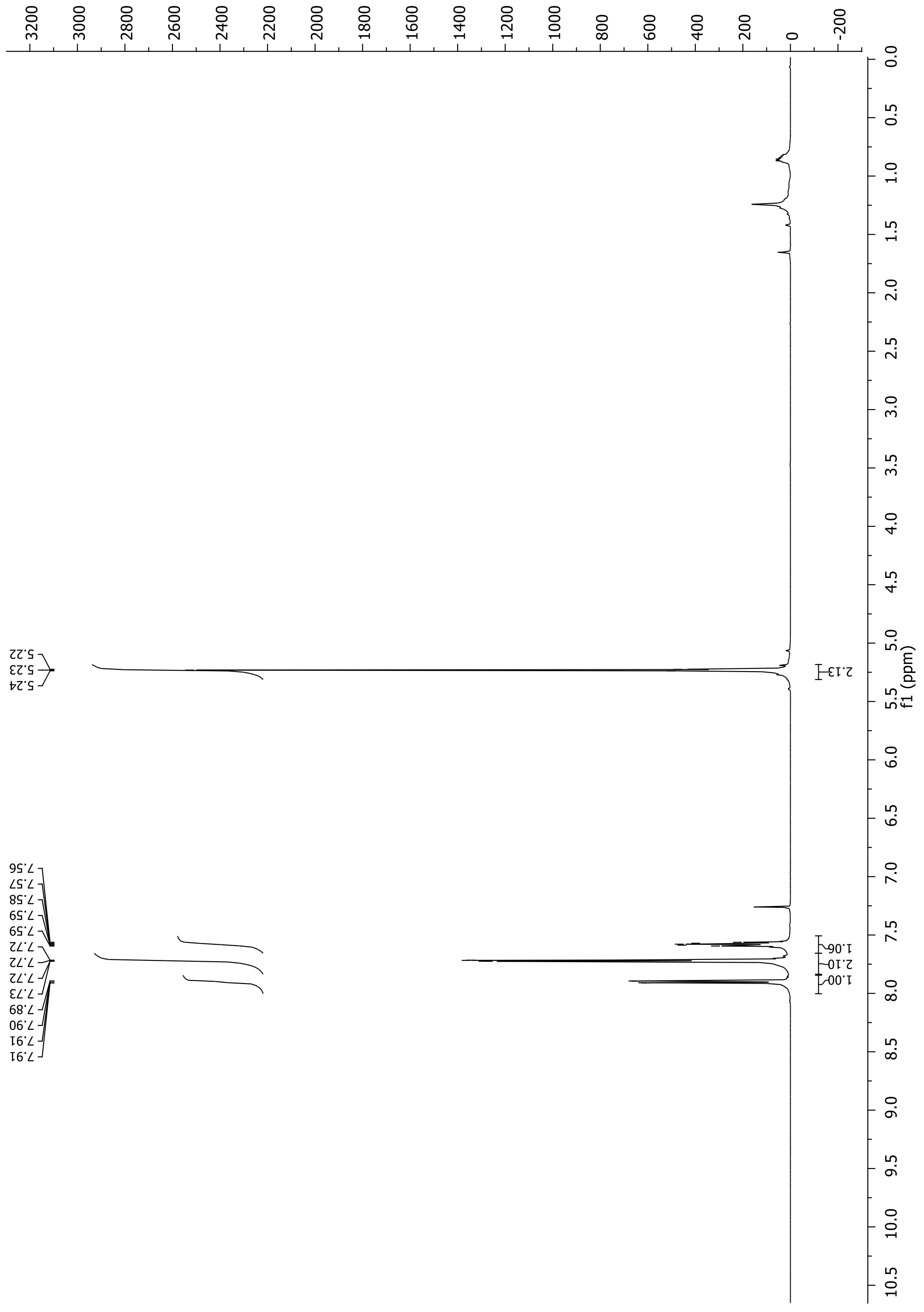
5.22
5.23
5.24
7.56
7.57
7.58
7.59
7.59
7.72
7.72
7.72
7.73
7.89
7.90
7.91
7.91
2.13
1.06
2.10
1.00
f1 (ppm)

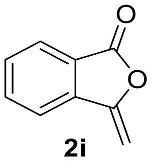

2i

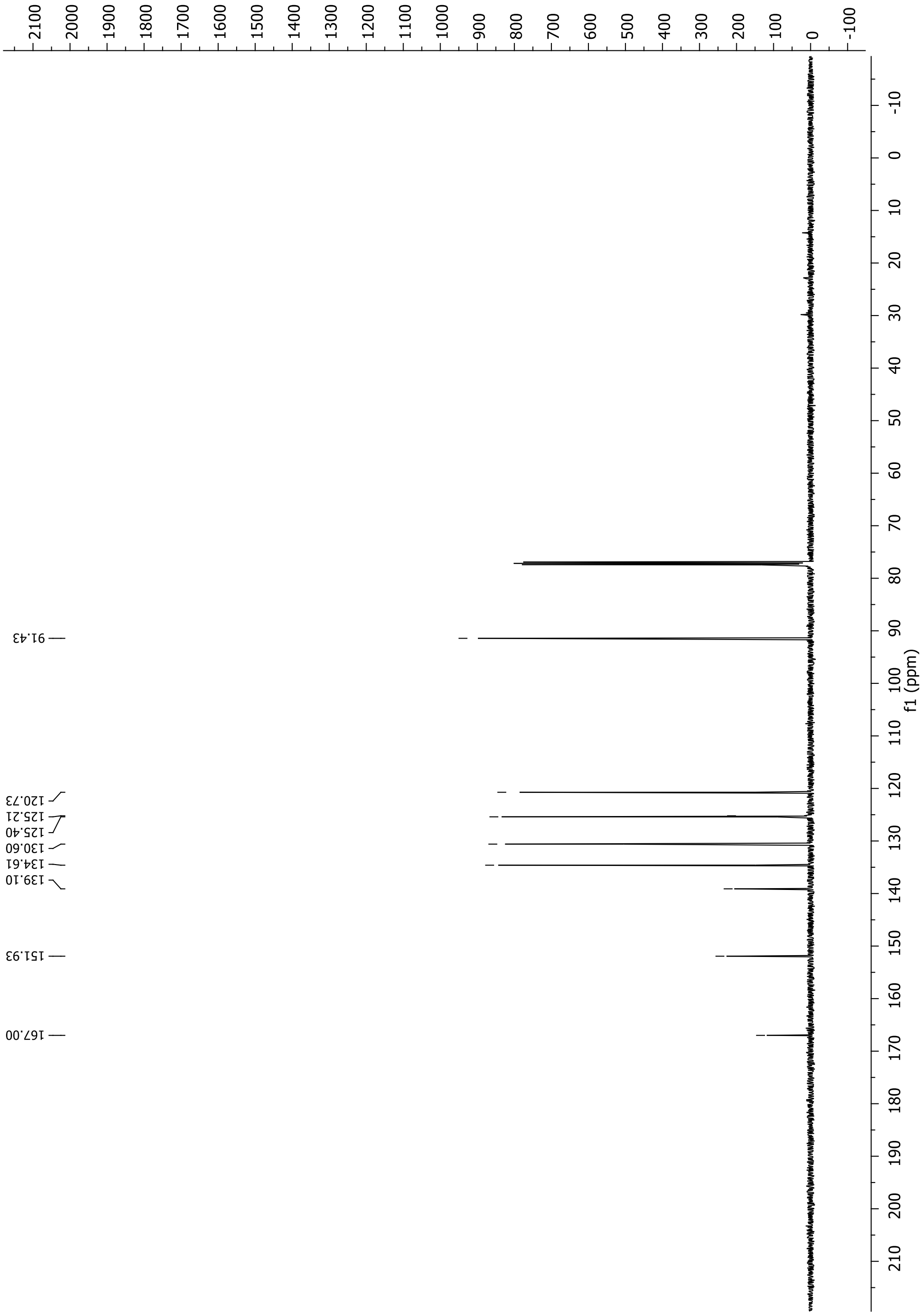

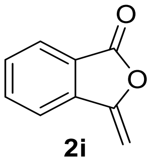

2i

Qualitative Analysis Report

**Data Filename**	LD1059.d	**Sample Name**	LD1059
**Sample Type**	Sample	**Position**	vial 1
**Instrument Name**	QTOF	**User Name**	QTOF-PC\admin
**Acq Method**	ACgroup_new.m	**Acquired Time**	2021-12-03 10:06:03
**IRM Calibration Status**	Success	**DA Method**	furfural 25.03.2021.m
**Comment**	LD1059		

**Acquisition SW**	6200 series TOF/6500 series
**Version**	Q-TOF B.05.00 (B5042.2)

Compounds

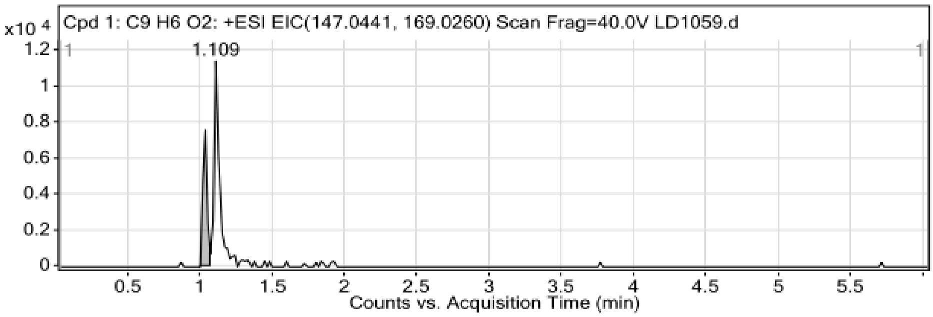

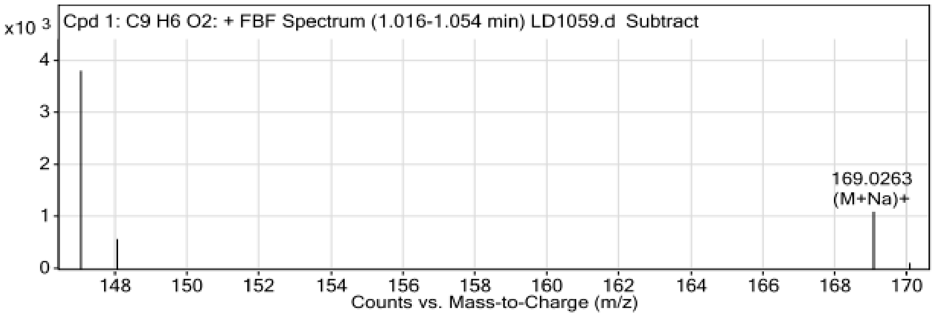

Peak List

m/z	z	Abund	Formula	Ion
147.0428	1	3810.38	C9H7O2	(M+H)+
148.0439	1	580.53	C9H7O2	(M+H)+
169.0263	1	1108.87	C9H6NaO2	(M+Na)+
170.0278	1	136.82	C9H6NaO2	(M+Na)+

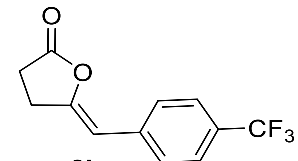

O
O
CF3

2j

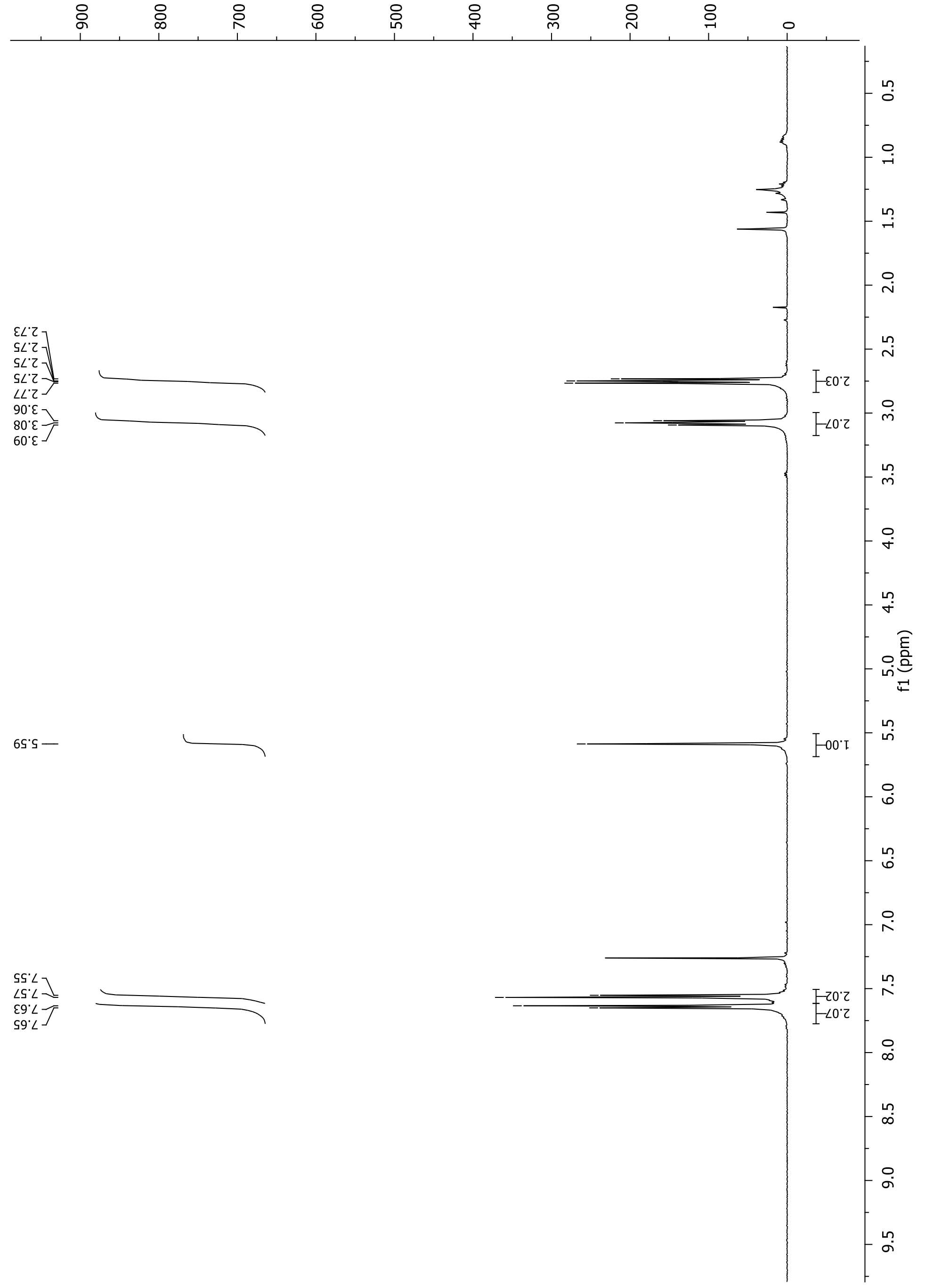

2.73
2.75
2.75
2.75
2.77
3.06
3.08
3.09
5.59
7.55
7.57
7.63
7.65
2.03
2.07
1.00
2.02
2.07
f1 (ppm)

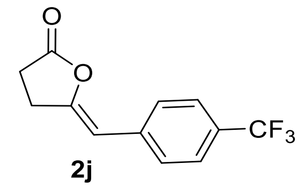

2j

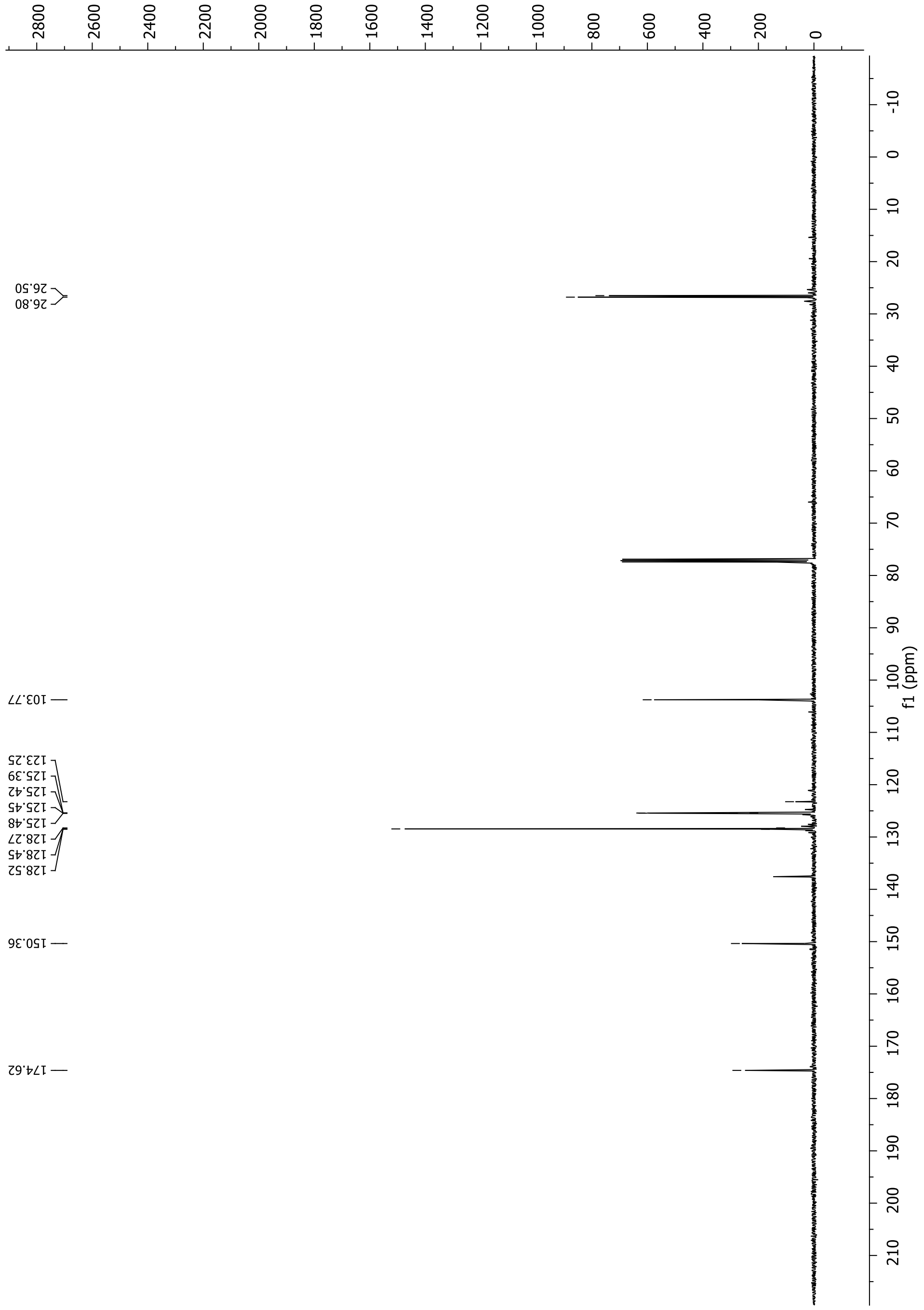
26.50
26.80
103.77
123.25
125.39
125.42
125.45
125.48
128.27
128.45
128.52
150.36
174.62
f1 (ppm)

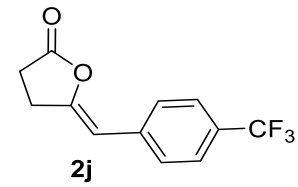

2j

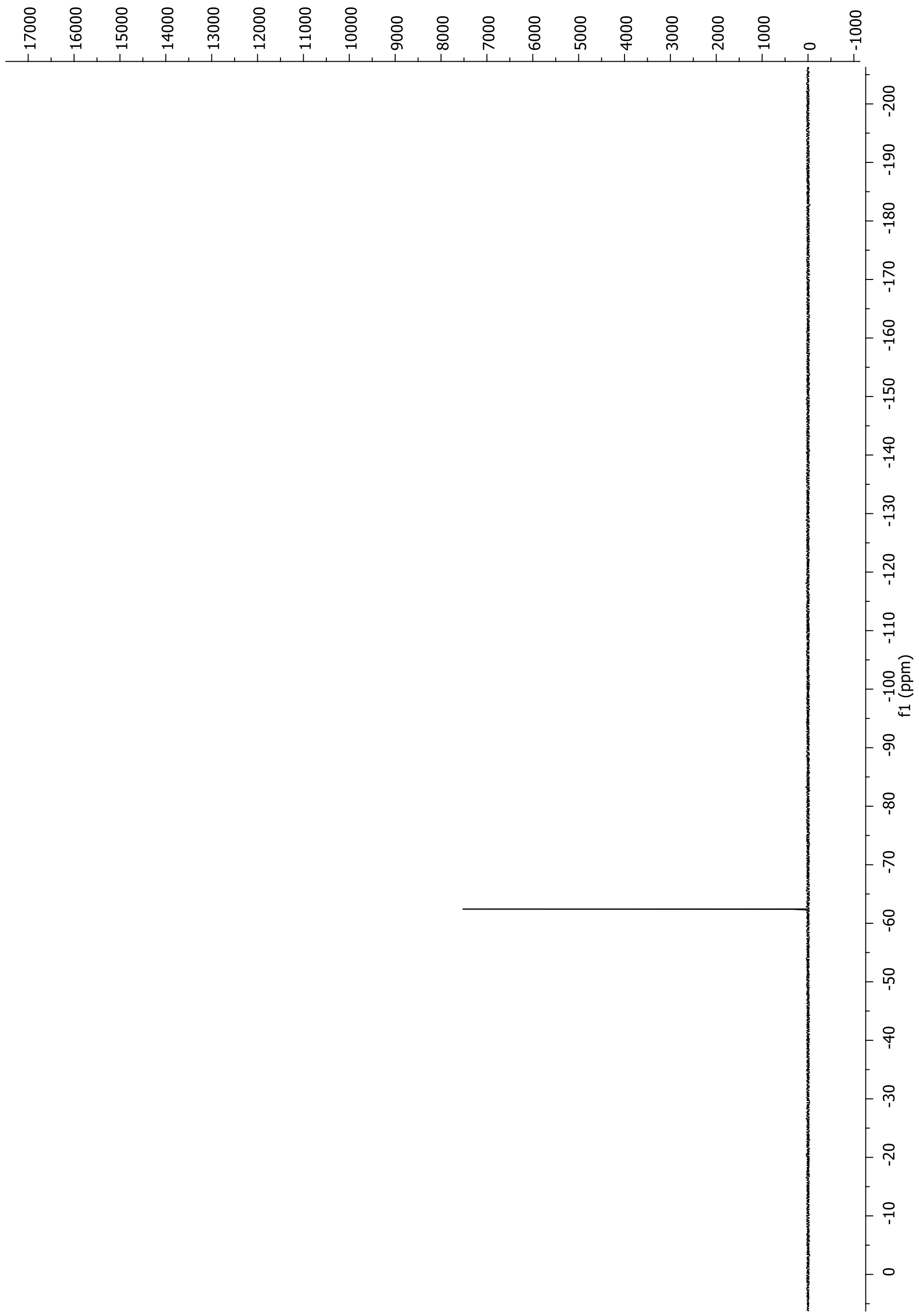

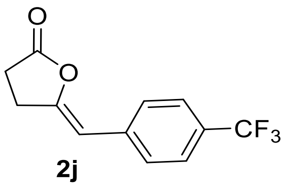

2j

Qualitative Analysis Report

**Data Filename**	LD1064.d	**Sample Name**	LD1064
**Sample Type**	Sample	**Position**	vial 1
**Instrument Name**	QTOF	**User Name**	QTOF-PC\admin
**Acq Method**	ACgroup_new.m	**Acquired Time**	2021-12-08 10:04:35
**IRM Calibration Status**	Success	**DA Method**	furfural 25.03.2021.m
**Comment**	LD1064		

**Acquisition SW**	6200 series TOF/6500 series
**Version**	Q-TOF B.05.00 (B5042.2)

Compounds

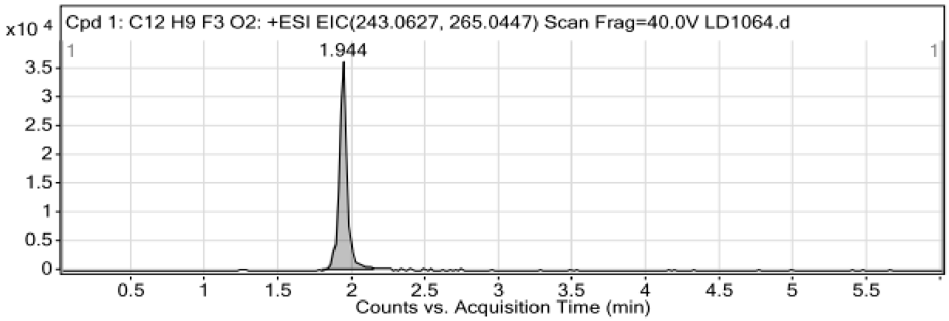

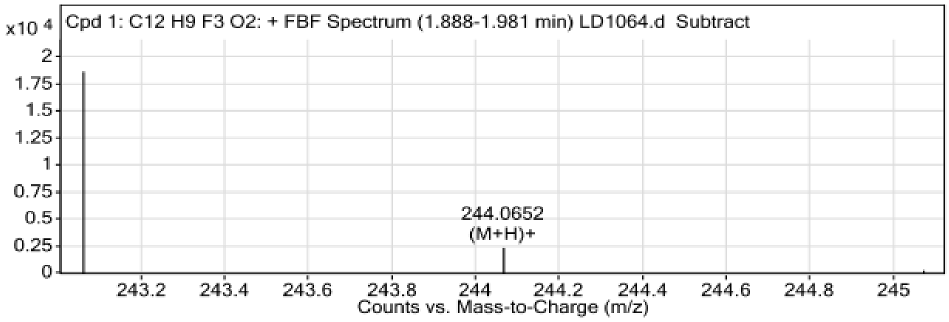

Peak List

m/z	z	Abund	Formula	Ion
243.0616	1	18663.46	C12H10F3O2	(M+H)+
244.0652	1	2481.86	C12H10F3O2	(M+H)+
245.0686	1	313.9	C12H10F3O2	(M+H)+

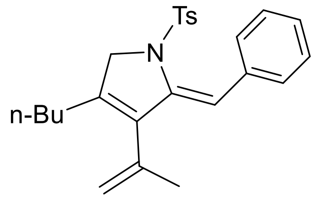
Ts
N
n-Bu

4a

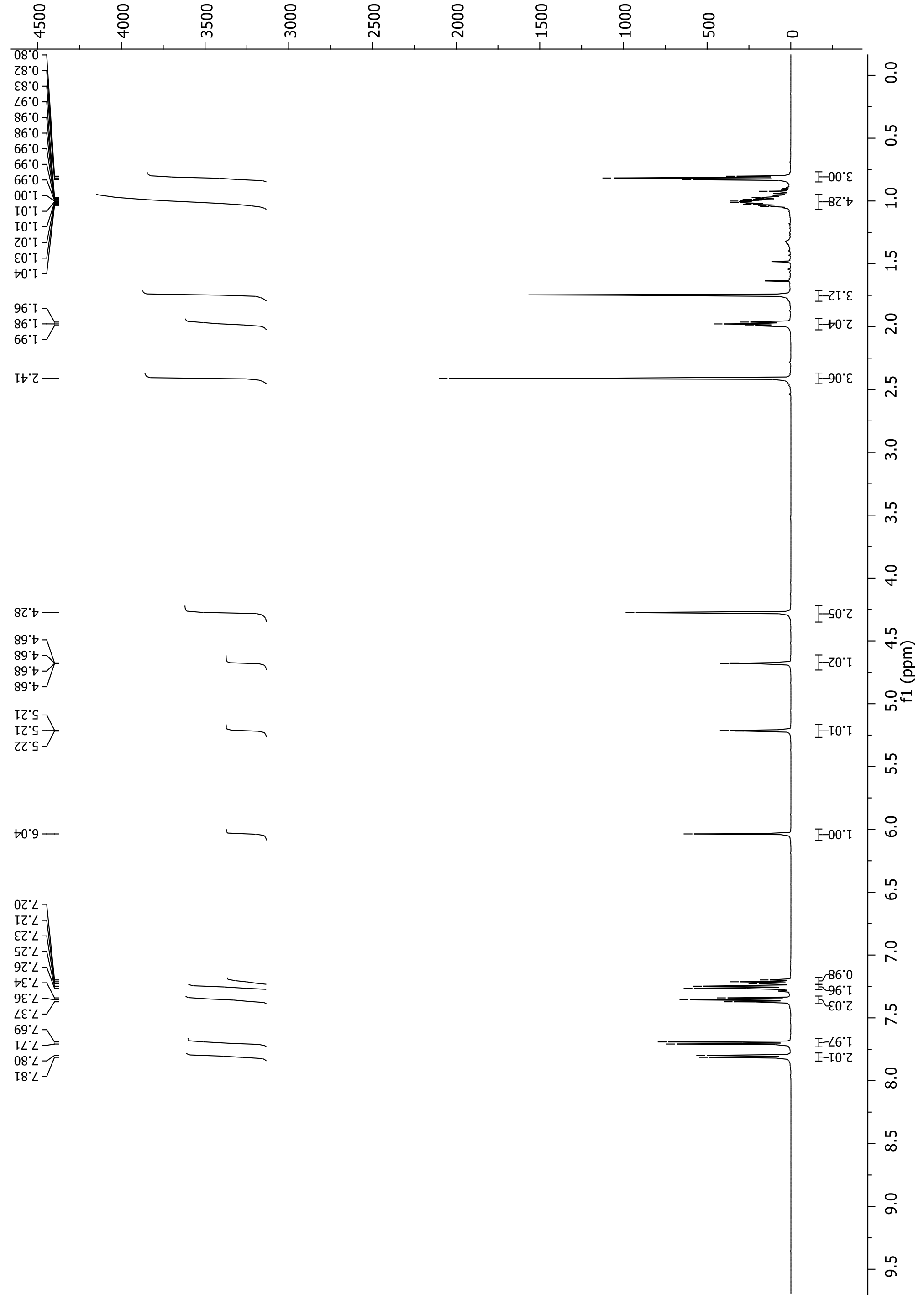
0.80
0.82
0.83
0.97
0.98
0.98
0.99
0.99
0.99
1.00
1.01
1.01
1.02
1.03
1.04
1.96
1.98
1.99
2.41
4.28
4.68
4.68
4.68
4.68
5.21
5.21
5.22
6.04
7.20
7.21
7.23
7.25
7.26
7.34
7.36
7.37
7.69
7.71
7.80
7.81
3.00
4.28
3.12
2.04
3.06
2.05
1.02
1.01
1.00
0.98
1.96
2.03
1.97
2.01
f1 (ppm)

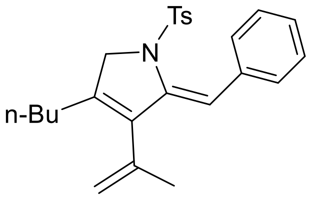
Ts
N
n-Bu

4a

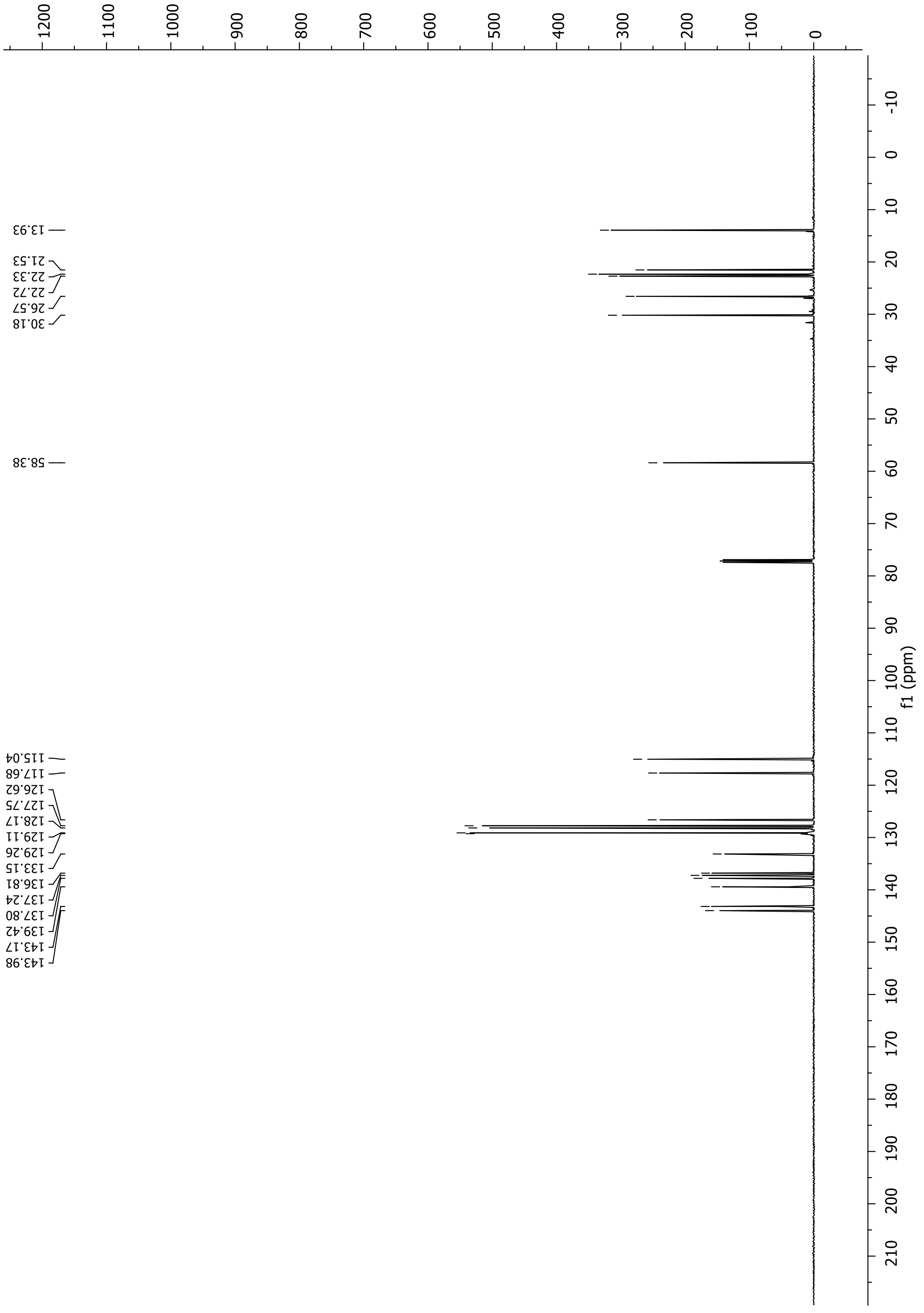
13.93
21.53
22.33
22.72
26.57
30.18
58.38
115.04
117.68
126.62
127.75
128.17
129.11
129.26
133.15
136.81
137.24
137.80
139.42
143.17
143.98
f1 (ppm)

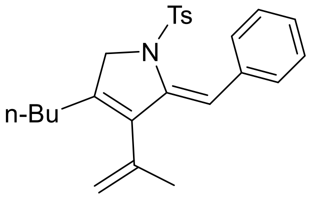

4a

Qualitative Analysis Report

**Data Filename**	LD1002.d	**Sample Name**	Unavailable
**Sample Type**	Unavailable	**Position**	Unavailable
**Instrument Name**	Unavailable	**User Name**	Unavailable
**Acq Method**		**Acquired Time**	Unavailable
**IRM Calibration Status**	Success	**DA Method**	furfural 25.03.2021.m
**Comment**	Sample information is unavailable		

Compounds

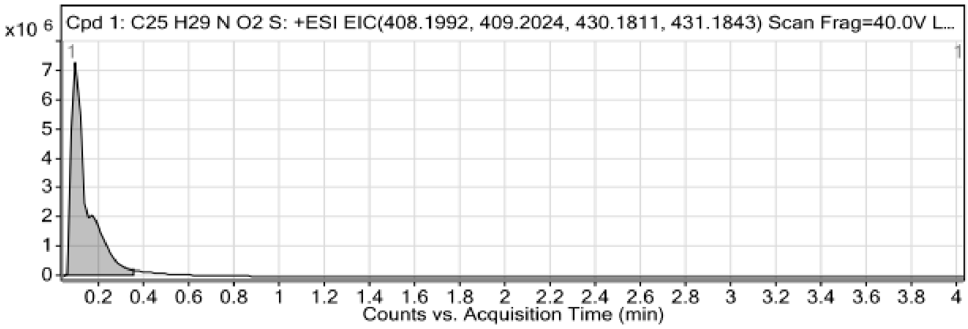

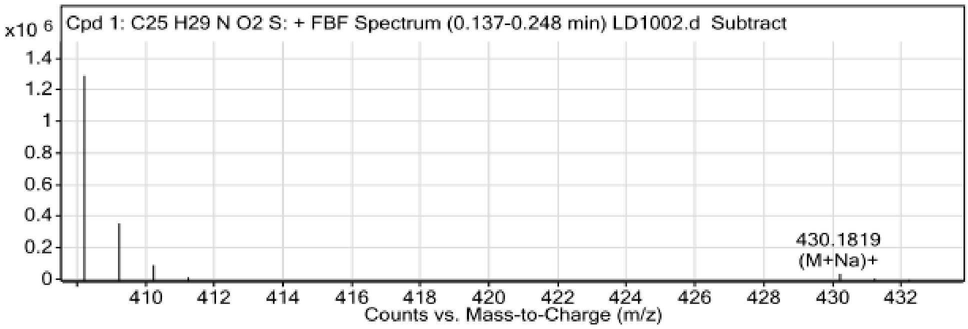

Peak List

m/z	z	Abund	Formula	Ion
408.1995	1	1297779.75	C25H30NO2S	(M+H)+
409.2032	1	363563.31	C25H30NO2S	(M+H)+
410.2011	1	99266.23	C25H30NO2S	(M+H)+
411.203	1	20373.52	C25H30NO2S	(M+H)+
412.2079	1	2977.46	C25H30NO2S	(M+H)+
430.1819	1	43540.45	C25H29NNaO2S	(M+Na)+
431.1851	1	12722.89	C25H29NNaO2S	(M+Na)+
432.1867	1	4295.62	C25H29NNaO2S	(M+Na)+
433.1868	1	944.84	C25H29NNaO2S	(M+Na)+

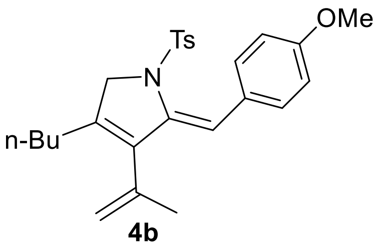

4b

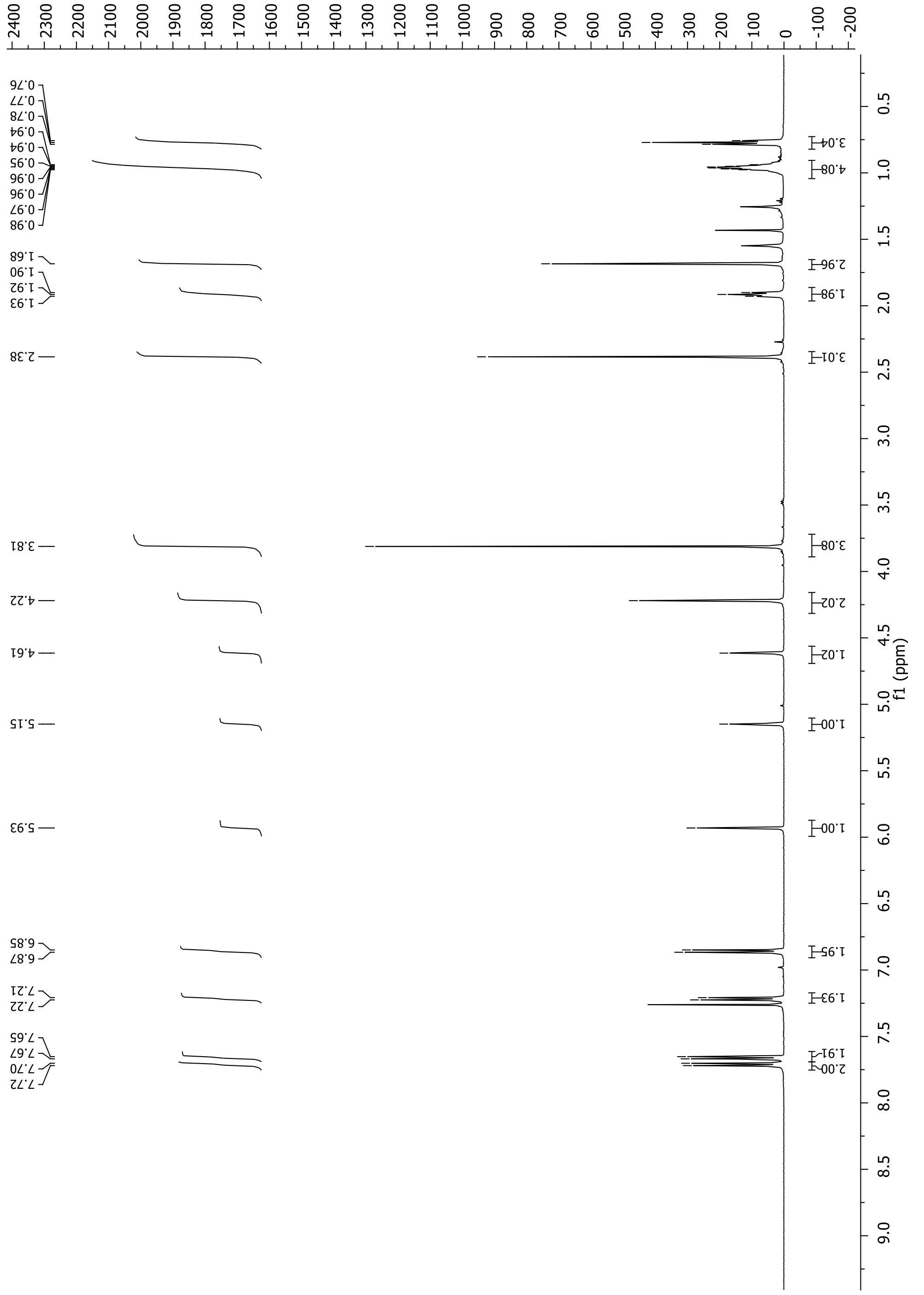

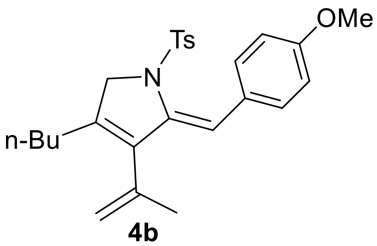
OMe
Ts
N
n-Bu

4b

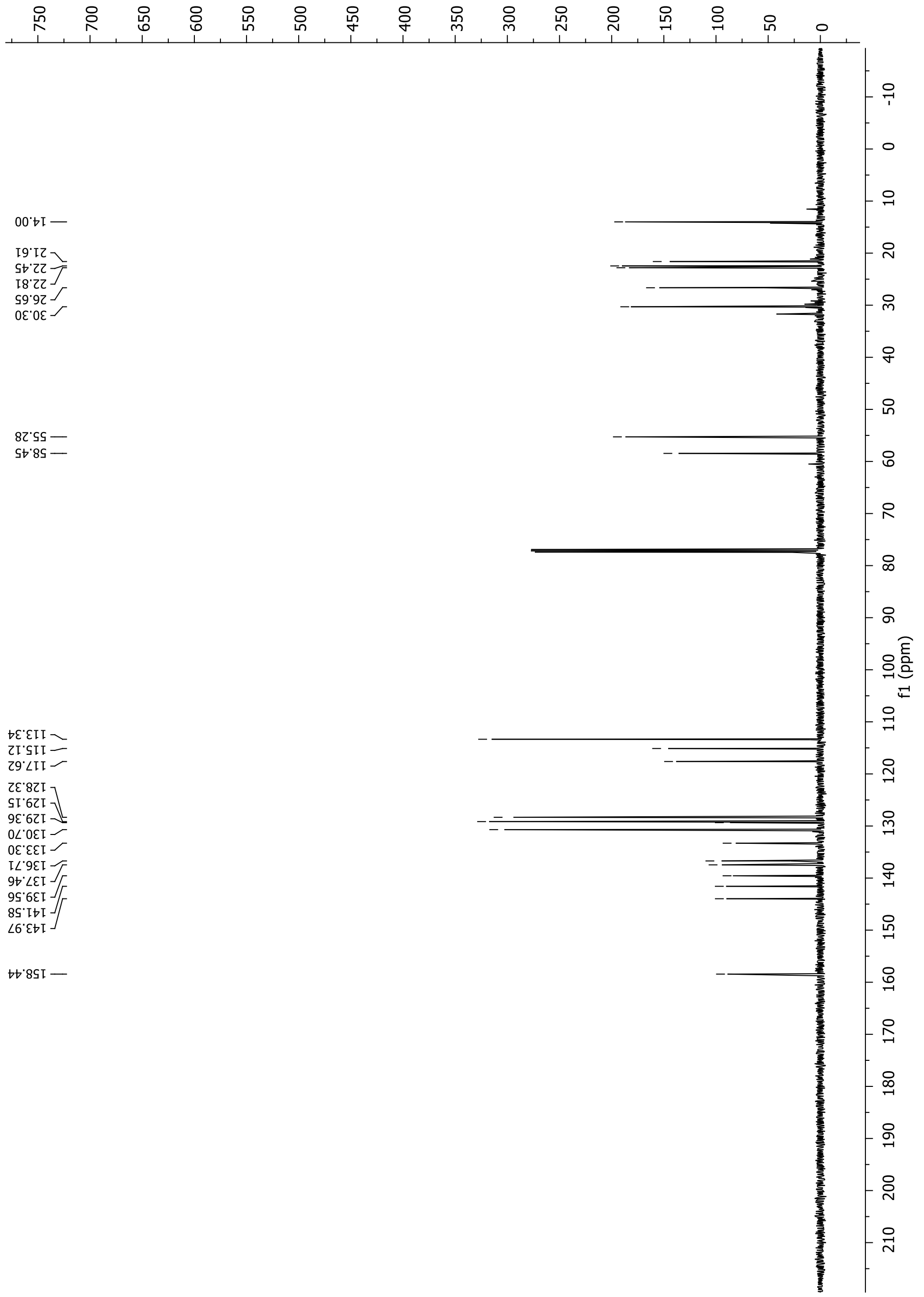
14.00
21.61
22.45
22.81
26.65
30.30
55.28
58.45
113.34
115.12
117.62
128.32
129.15
129.36
130.70
133.30
136.71
137.46
139.56
141.58
143.97
158.44
f1 (ppm)

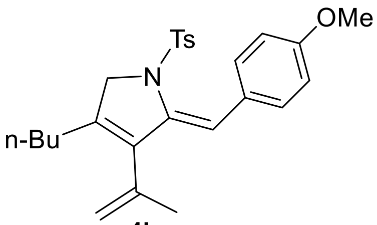

4b

Qualitative Analysis Report

**Data Filename**	LD1001.d	**Sample Name**	LD1001
**Sample Type**	Sample	**Position**	vial 1
**Instrument Name**	QTOF	**User Name**	QTOF-PC\admin
**Acq Method**	ACgroup_new_no_column.m	**Acquired Time**	2022-03-29 11:11:00
**IRM Calibration Status**	Success	**DA Method**	furfural 25.03.2021.m
**Comment**			

**Acquisition SW**	6200 series TOF/6500 series
**Version**	Q-TOF B.05.00 (B5042.2)

Compounds

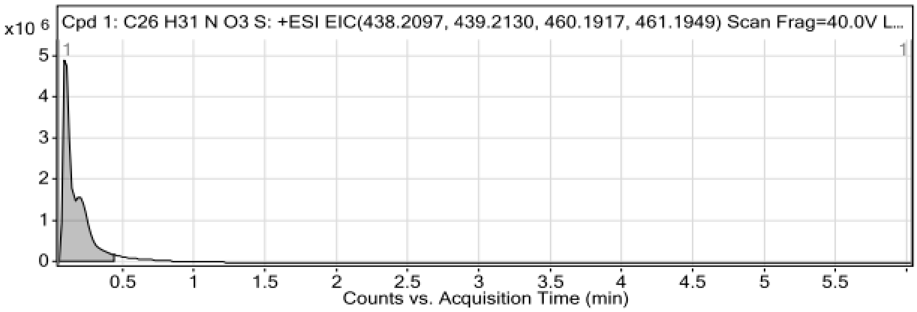

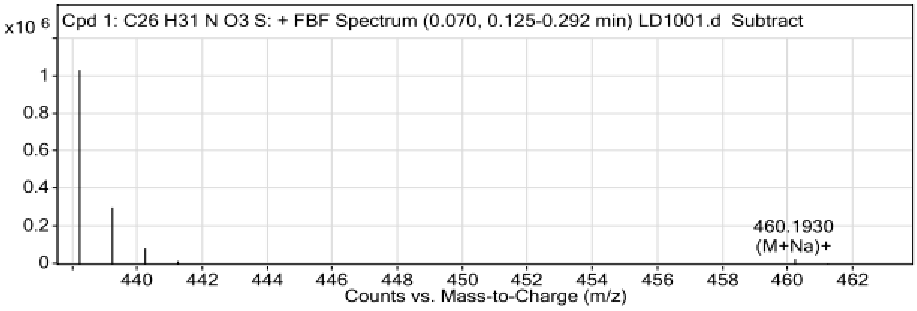

Peak List

m/z	z	Abund	Formula	Ion
438.2112	1	1036139.56	C26H32NO3S	(M+H)+
439.2145	1	302991.34	C26H32NO3S	(M+H)+
440.2127	1	86475.06	C26H32NO3S	(M+H)+
441.2143	1	18562.3	C26H32NO3S	(M+H)+
442.2166	1	2818.17	C26H32NO3S	(M+H)+
460.193	1	29152.61	C26H31NNaO3S	(M+Na)+
461.1962	1	9094.16	C26H31NNaO3S	(M+Na)+
462.1957	1	2741.51	C26H31NNaO3S	(M+Na)+
463.1964	1	634.7	C26H31NNaO3S	(M+Na)+

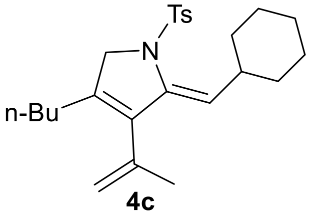

4c

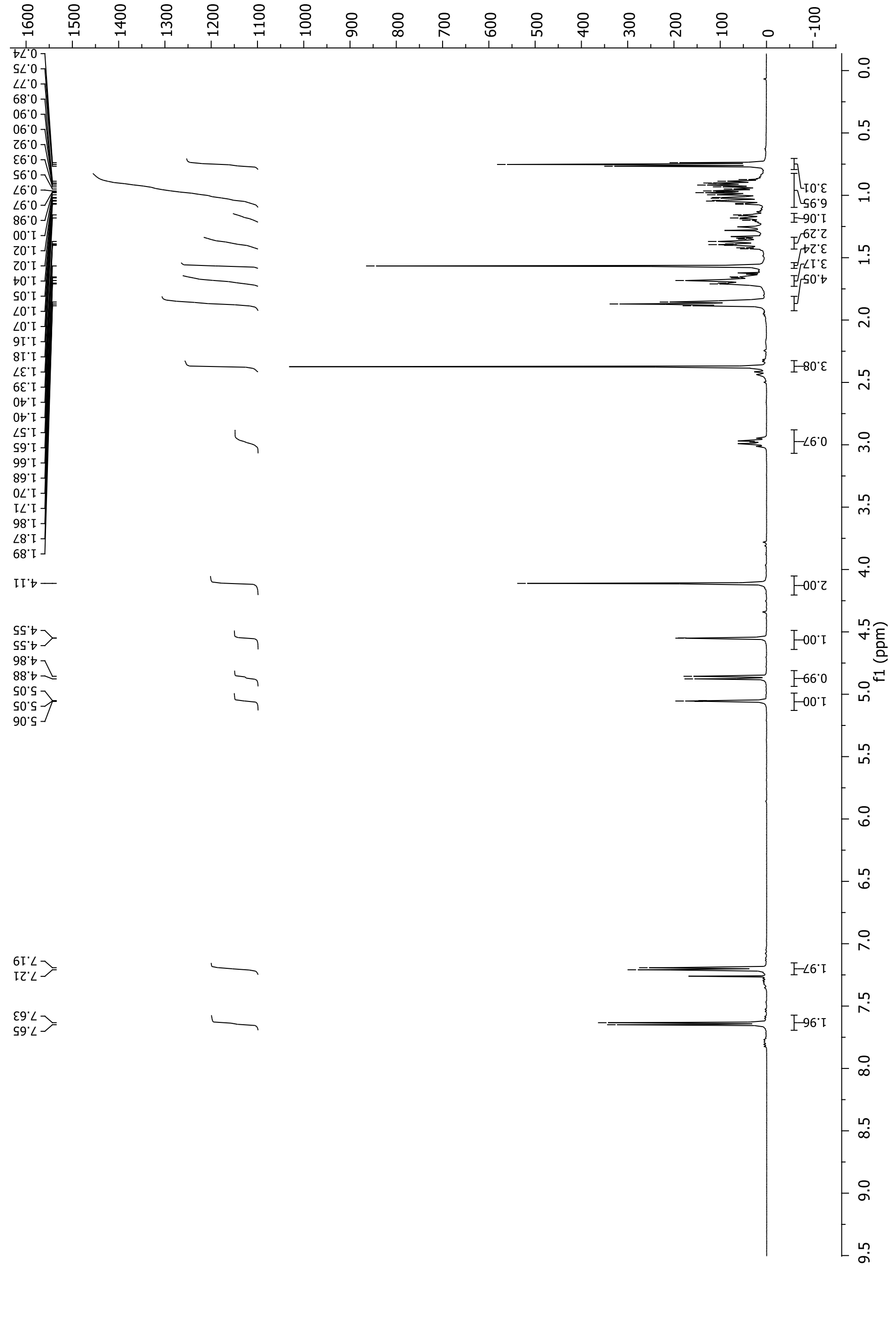

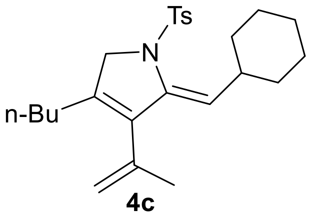
Ts
N
n-Bu

4c

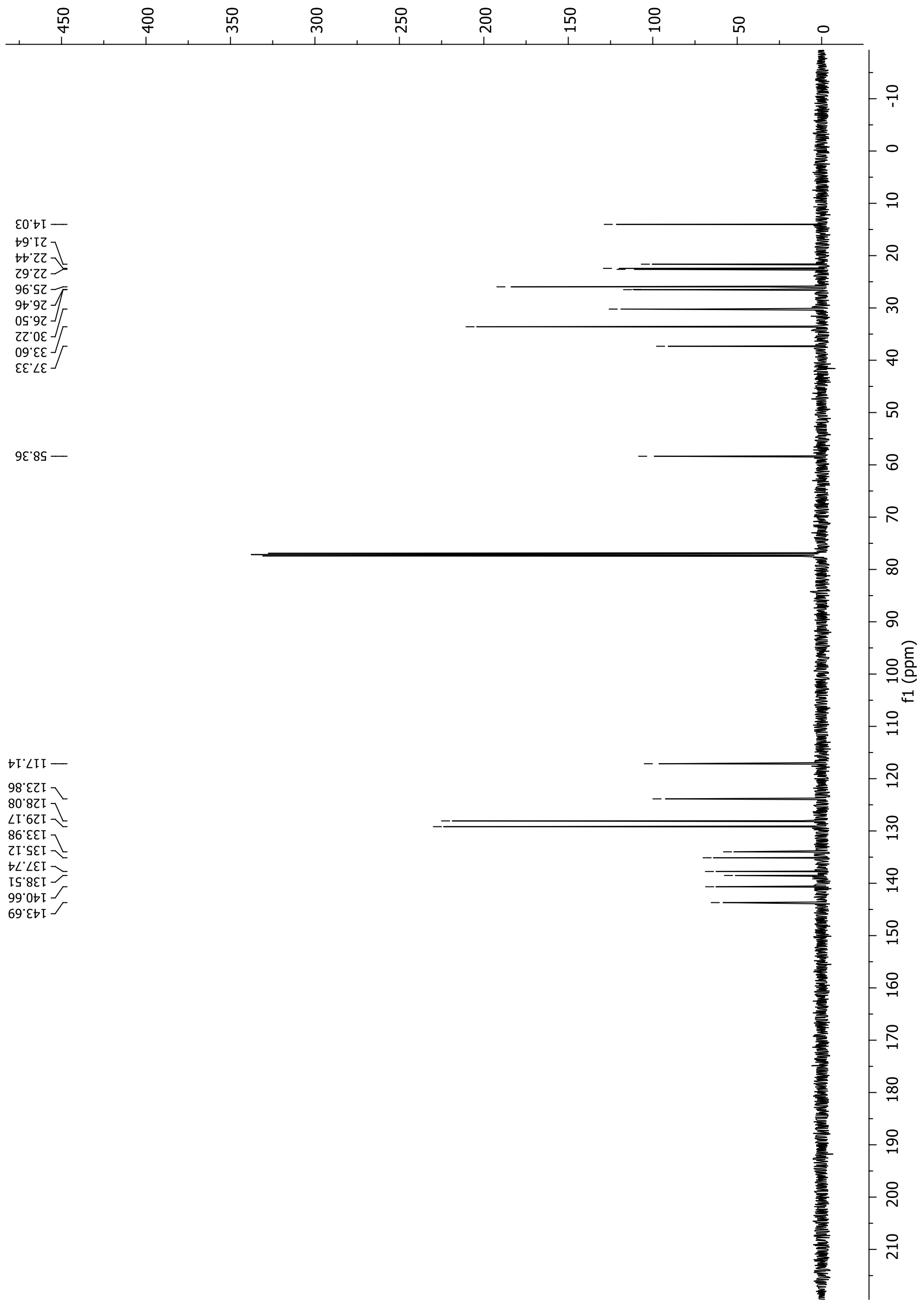
14.03
21.64
22.44
22.62
25.96
26.46
26.50
30.22
33.60
37.33
58.36
117.14
123.86
128.08
129.17
133.98
135.12
137.74
138.51
140.66
143.69
f1 (ppm)

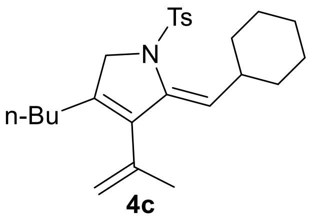

4c

Qualitative Analysis Report

**Data Filename**	LD1065.d	**Sample Name**	LD1065
**Sample Type**	Sample	**Position**	vial 1
**Instrument Name**	QTOF	**User Name**	QTOF-PC\admin
**Acq Method**	ACgroup_new_no_column.m	**Acquired Time**	2022-03-29 11:03:59
**IRM Calibration Status**	Success	**DA Method**	furfural 25.03.2021.m
**Comment**			

**Acquisition SW**	6200 series TOF/6500 series
**Version**	Q-TOF B.05.00 (B5042.2)

Compounds

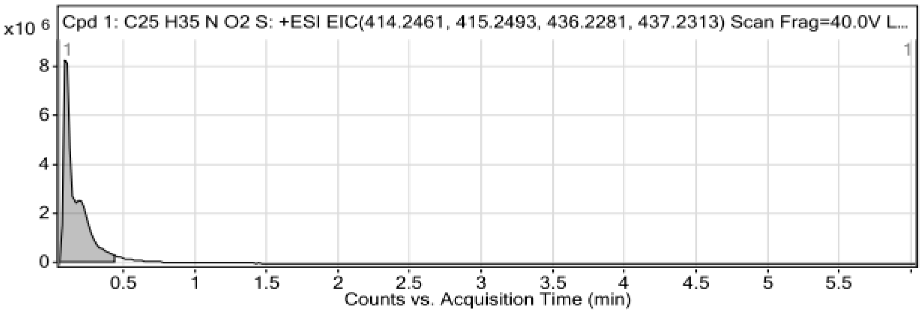

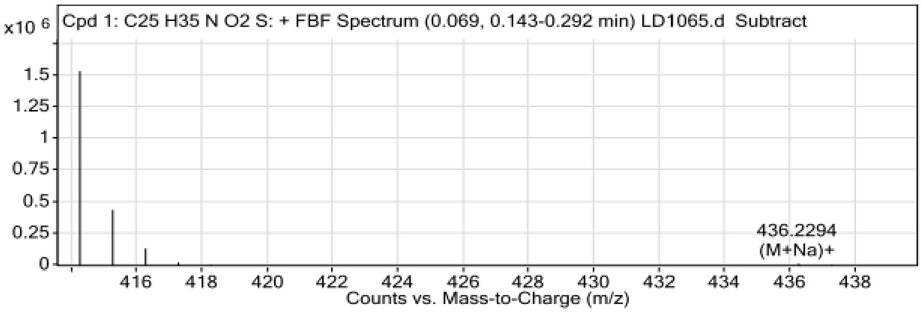

Peak List

m/z	z	Abund	Formula	Ion
414.247	1	1538063.75	C25H36NO2S	(M+H)+
415.2507	1	440475.06	C25H36NO2S	(M+H)+
416.2515	1	142290.47	C25H36NO2S	(M+H)+
417.2533	1	29317.34	C25H36NO2S	(M+H)+
418.2554	1	5653.03	C25H36NO2S	(M+H)+
436.2294	1	22091.54	C25H35NNaO2S	(M+Na)+
437.2331	1	6881.63	C25H35NNaO2S	(M+Na)+
438.239	1	3462.81	C25H35NNaO2S	(M+Na)+
439.2454	1	861.85	C25H35NNaO2S	(M+Na)+

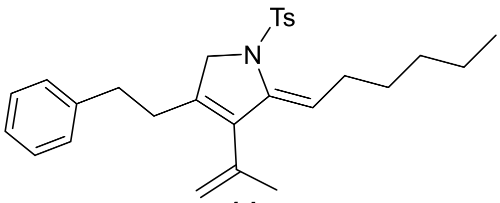
Ts
N

4d

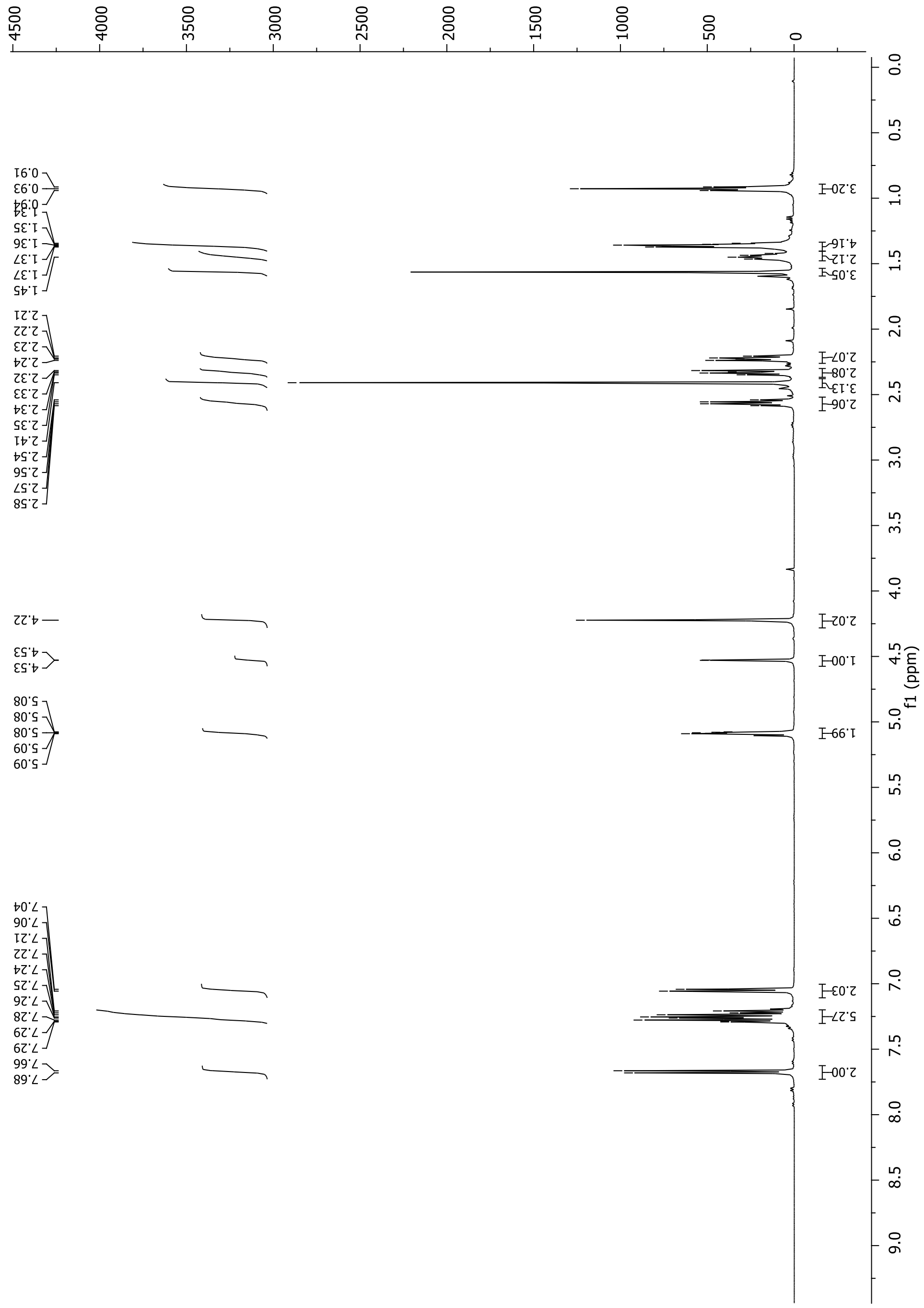
0.91
0.93
0.94
1.34
1.35
1.36
1.37
1.37
1.45
2.21
2.22
2.23
2.24
2.32
2.33
2.34
2.35
2.41
2.54
2.56
2.57
2.58
4.22
4.53
4.53
5.08
5.08
5.08
5.09
5.09
7.04
7.06
7.21
7.22
7.24
7.25
7.26
7.28
7.29
7.29
7.66
7.68
3.20
4.16
2.12
3.05
2.07
2.08
3.13
2.06
2.02
1.00
1.99
2.03
5.27
2.00
f1 (ppm)

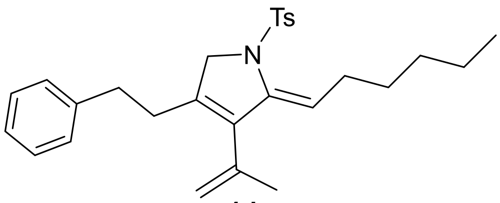

4d

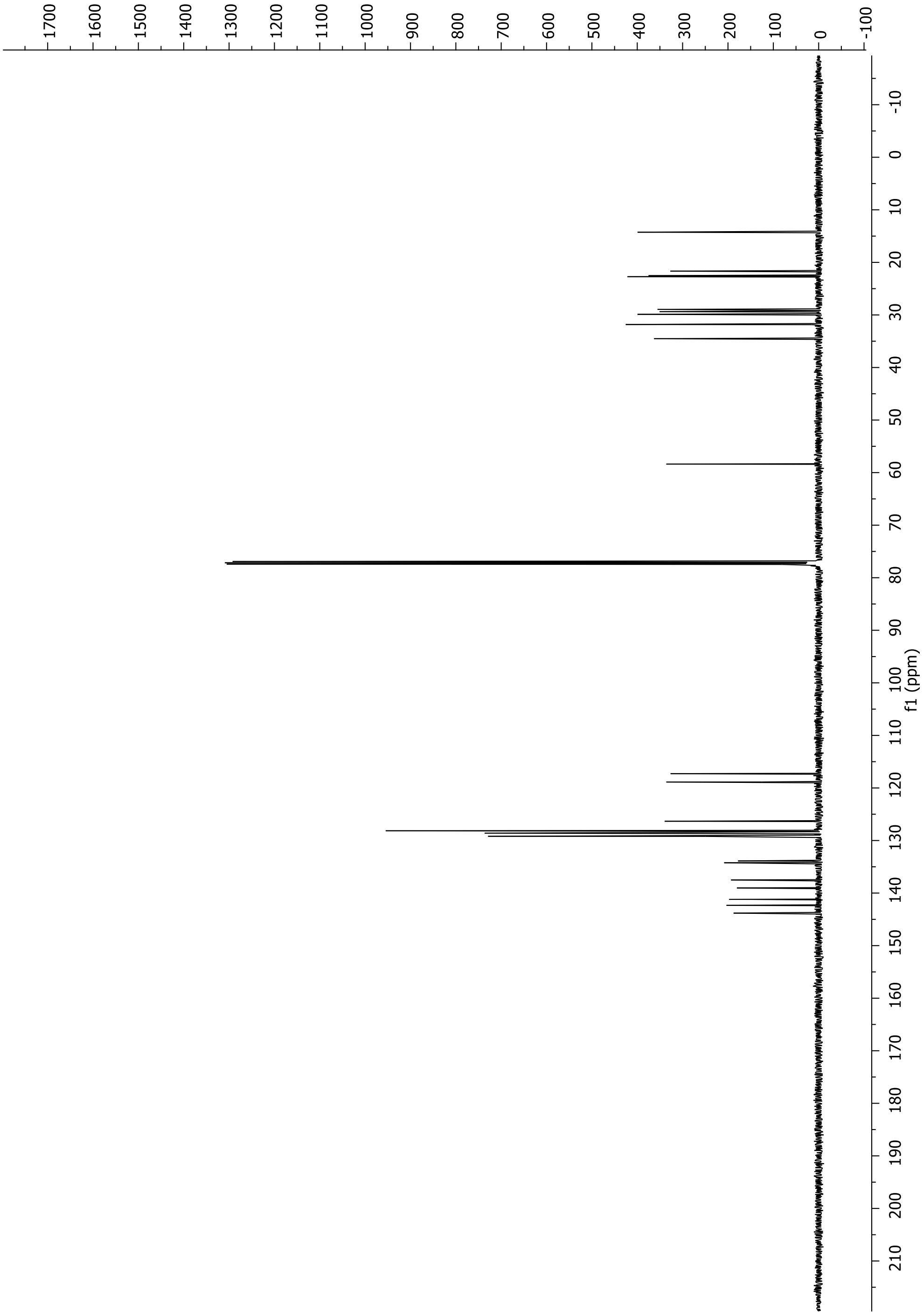

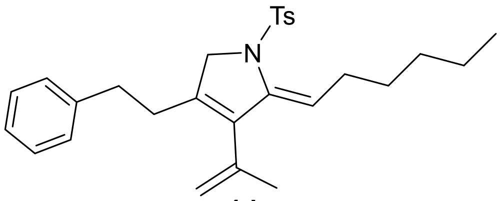

4d

Qualitative Analysis Report

**Data Filename**	LD956.d	**Sample Name**	LD956
**Sample Type**	Sample	**Position**	vial 1
**Instrument Name**	QTOF	**User Name**	QTOF-PC\admin
**Acq Method**	ACgroup_new_no_column.m	**Acquired Time**	2022-03-29 10:56:50
**IRM Calibration Status**	Success	**DA Method**	furfural 25.03.2021.m
**Comment**			

**Acquisition SW**	6200 series TOF/6500 series
**Version**	Q-TOF B.05.00 (B5042.2)

Compounds

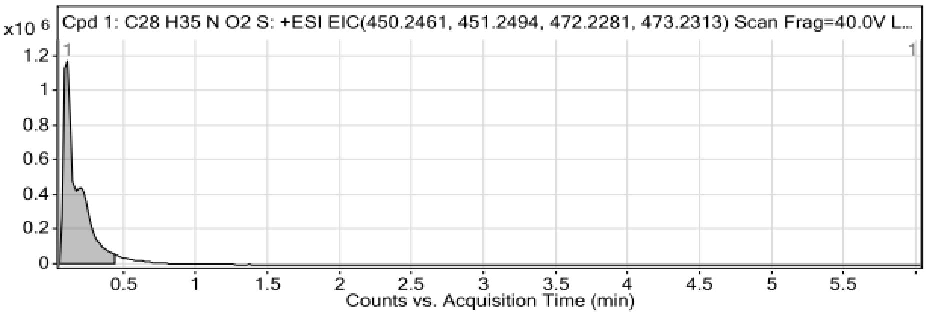

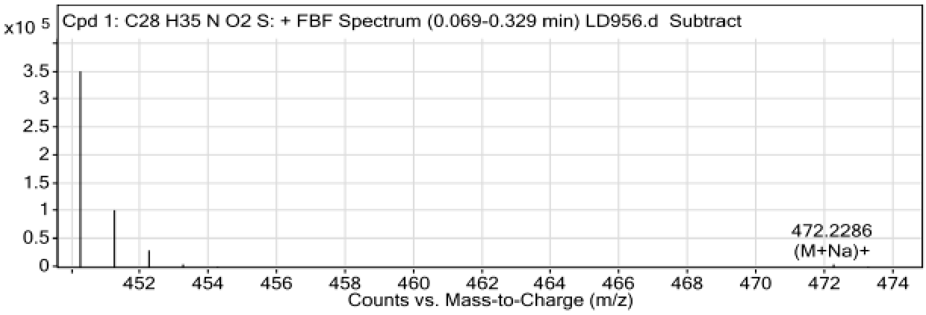

Peak List

m/z	z	Abund	Formula	Ion
450.2464	1	351618.91	C28H36NO2S	(M+H)+
451.2496	1	103865.88	C28H36NO2S	(M+H)+
452.2482	1	30816.34	C28H36NO2S	(M+H)+
453.2472	1	7285.8	C28H36NO2S	(M+H)+
454.2422	1	1696.12	C28H36NO2S	(M+H)+
472.2286	1	6857.66	C28H35NNaO2S	(M+Na)+
473.2301	1	2370.59	C28H35NNaO2S	(M+Na)+
474.234	1	907.45	C28H35NNaO2S	(M+Na)+

Electron Microscopy data

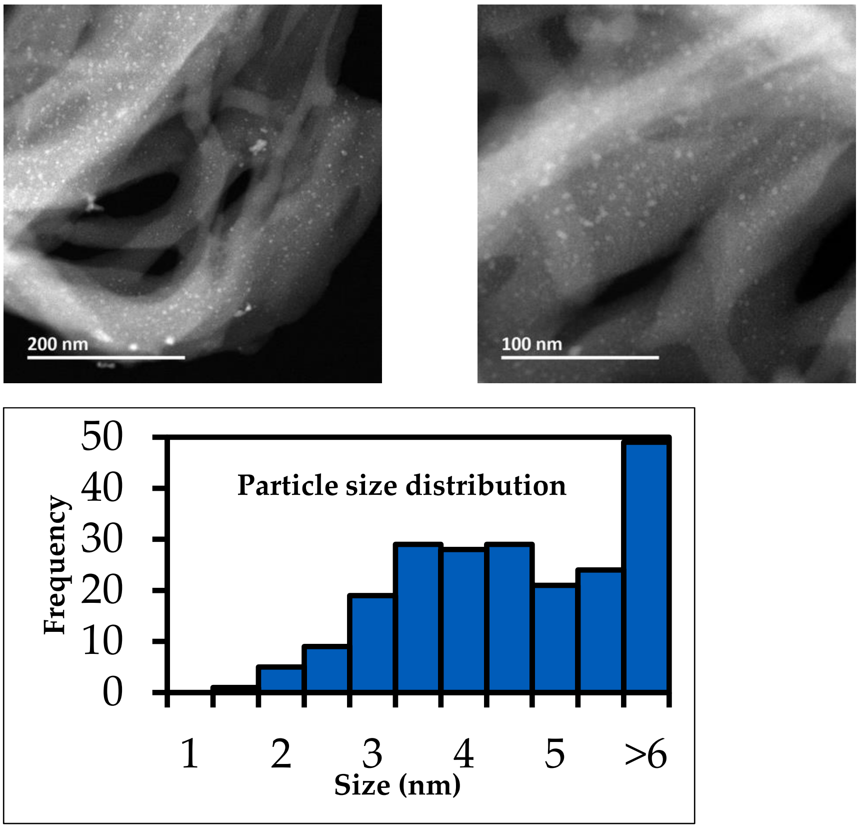

Figure S1. HAADF-STEM images of well-dispersed Au nanoparticles on MCC-McP-Au^0. Particles size distribution on MCC-McP-Au^0 according to STEM analysis.

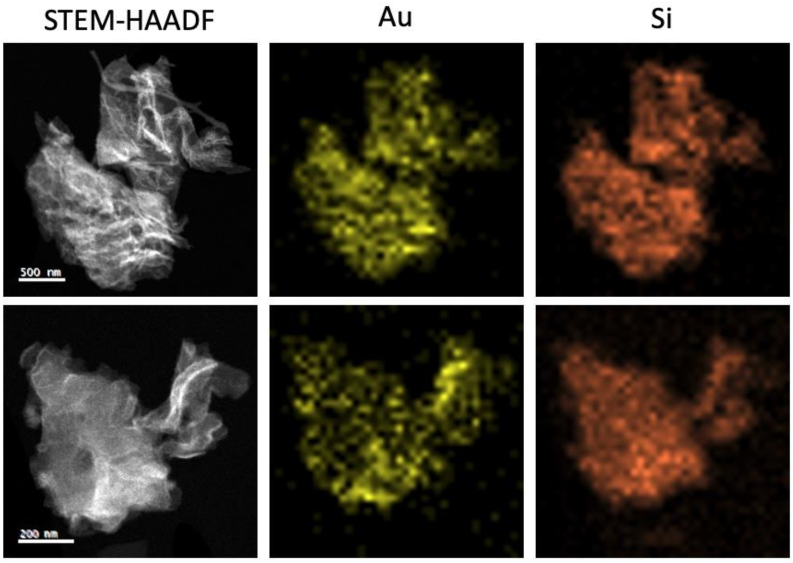

Figure S2. HAADF-STEM images, and the corresponding Au L-edge and Si K-edge EDS map of the MCC-McP-Au^0/Au^I,. No nanoparticle is observed. The variations of intensity in EDS maps reflect the thickness of the samples, which is consistent with HAADF-STEM image contrasts.

FT-IR spectra of materials and catalysts

FT-IR spectrum of MCC:

The broad absorption bands in all spectra at around 3330 cm^{-1} and 2892 cm^{-1} correspond to the O-H and C-H stretching vibrations, respectively. The bands at around 1637 cm^{-1} is attributed to the O-H bending vibrations of the hydroxyl groups of absorbed water. The absorption peaks at about 1162 cm^{-1} and 1103 cm^{-1} are attributed to the stretching vibrations of C–C and C–O, respectively. The absorption peaks about 1028 cm^{-1} comes from the vibration of C-O-C in the pyranose ring.

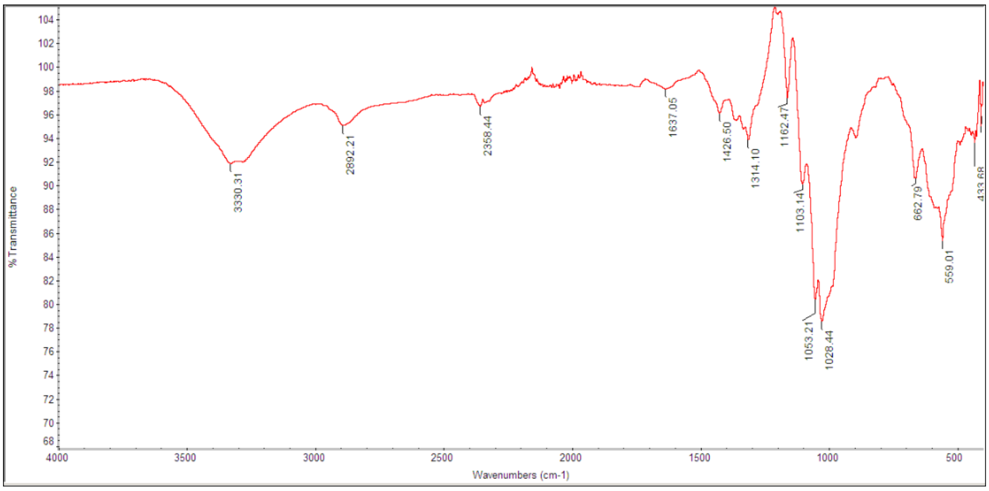

Figure S3. FT-IR spectrum of MCC.

FT-IR spectrum of MCC-McP:

The broad absorption bands in all spectra at around 3330 cm^{-1} and 2892 cm^{-1} are related to the O-H and C-H stretching vibrations, respectively. The bands at around 1636 cm^{-1} is attributed to the O-H bending vibrations of the hydroxyl groups of absorbed water. The absorption peaks at about 1162 cm^{-1} and 1103 cm^{-1} are attributed to the stretching vibrations of C–C and C–O, respectively. The absorption peaks about 1028 cm^{-1} comes from the vibration of C-O-C in the pyranose ring. Specific bands corresponding to the Si-O-Si and Si-O-Cellulose bridges (around 1135 and 1150 cm^{-1}, respectively) were overlapped with the large and intense C-O-C vibration bands of cellulose in the same spectral region. The low degree of substitution did also show very small amount of the -SH band at around 2550 cm^{-1}.

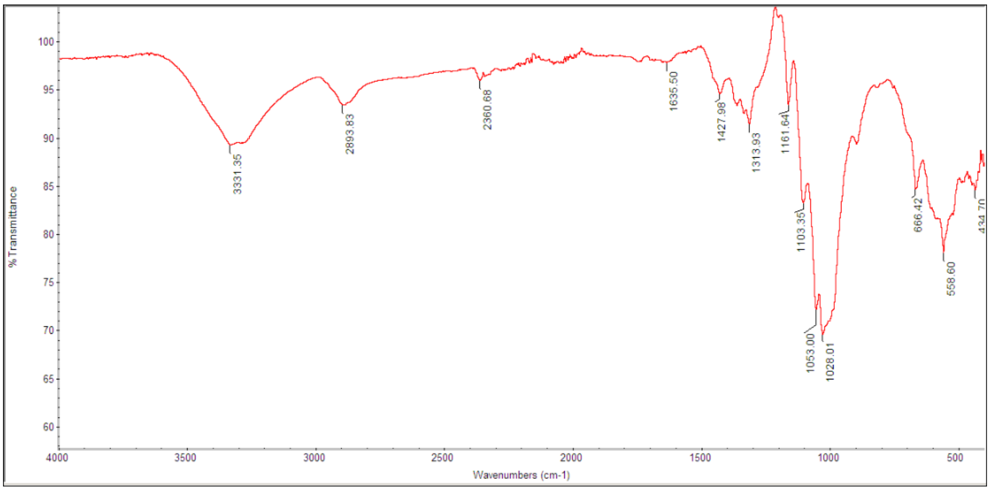

Figure S4. FT-IR spectrum of MCC-McP.

FT-IR spectrum of MCC-McP-Au^0

The broad absorption bands at around 3331 cm^{-1} and 2895 cm^{-1} correspond to the O-H and C-H stretching vibrations, respectively. The bands at around 1637 cm^{-1} is attributed to the O-H bending vibrations of the hydroxyl groups of absorbed water. The absorption peaks at about 1162 cm^{-1} and 1103 cm^{-1} are attributed to the stretching vibrations of C–C and C–O, respectively. The absorption peaks about 1028 cm^{-1} comes from the vibration of C-O-C in the pyranose ring. Specific bands corresponding to the Si-O-Si and Si-O-Cellulose bridges (around 1135 and 1150 cm^{-1}, respectively) were overlapped with the large and intense C-O-C vibration bands of cellulose in the same spectral region. The low degree of substitution did also show very small amount of the -SH band at 2550 cm^{-1}.

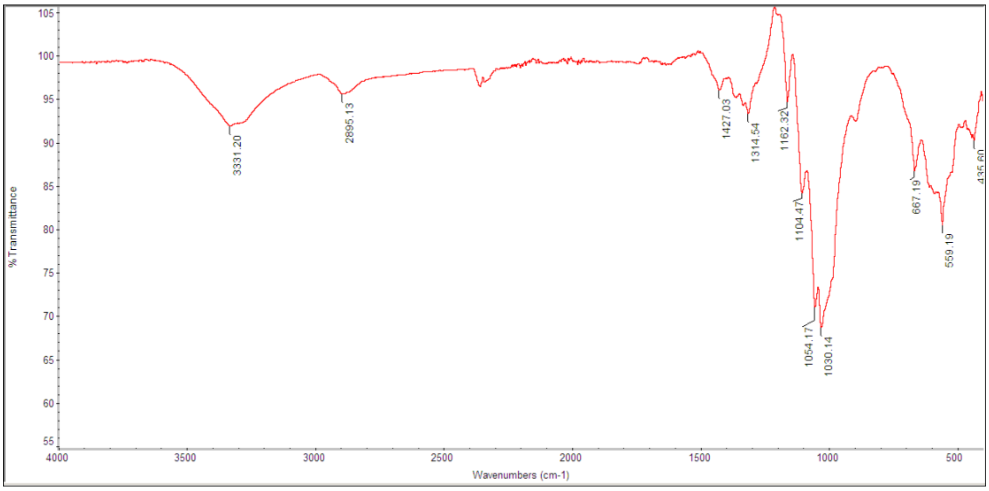

Figure S5. FT-IR spectrum of MCC-McP-Au^0.

FT-IR spectrum of MCC-AmP

The broad absorption bands in all spectra at around 3330 cm-1 and 2898 cm-1 correspond to the O-H and C-H stretching vibrations, respectively. The bands at around 1636 cm^{-1} is attributed to the O-H bending vibrations of the hydroxyl groups of absorbed water. The absorption peaks at about 1160 cm-1 and 1104 cm-1 are attributed to the stretching vibrations of C–C and C–O, respectively. The absorption peaks about 1030 cm-1 comes from the vibration of C-O-C in the pyranose ring. Specific bands corresponding to the Si-O-Si and Si-O-Cellulose bridges (around 1135 and 1150 cm^{-1}, respectively) were overlapped with the large andintense C-O-C-vibration bands of cellulose in the same spectral region. The low degree of substitution did also show very small amount of the -NH band at 1150 cm^{-1}.

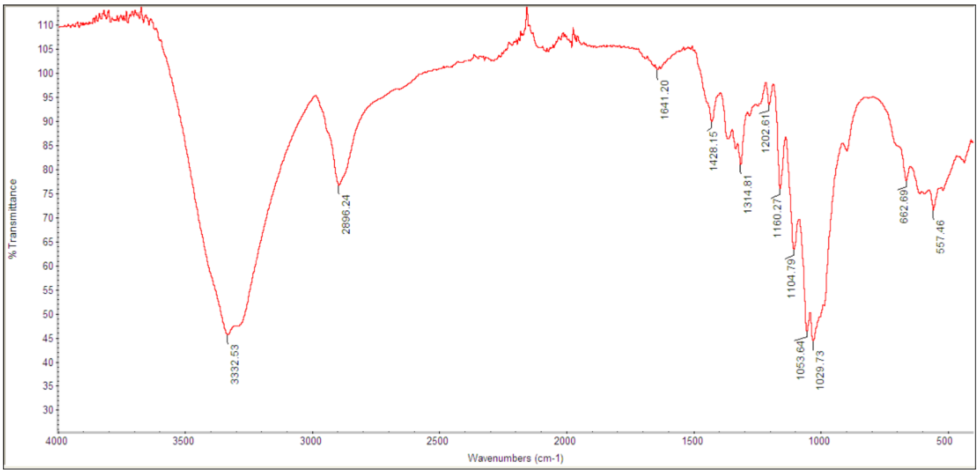

Figure S6. FT-IR spectrum of MCC-AmP.

FT-IR spectrum of MCC-AmP-Au^0

The broad absorption bands at 3333 cm^{-1} and 2895 cm^{-1} are attributed to the O-H and C-H stretching vibrations, respectively. The bands at around 1630 cm^{-1} is attributed to the O-H bending vibrations of the hydroxyl groups of absorbed water. The absorption peaks at about 1162 cm^{-1} and 1106 cm^{-1} correspond to the stretching vibrations of C–C and C–O, respectively. The absorption peaks about 1032 cm^{-1} comes from the vibration of C-O-C in the pyranose ring. Specific bands corresponding to the Si-O-Si and Si-O-Cellulose bridges (around 1135 and 1150 cm^{-1}, respectively) were overlapped with the large and intense C-O-C vibration bands of cellulose in the same spectral region. The low degree of substitution did also show very small amount of the -NH band at 1150 cm^{-1}.

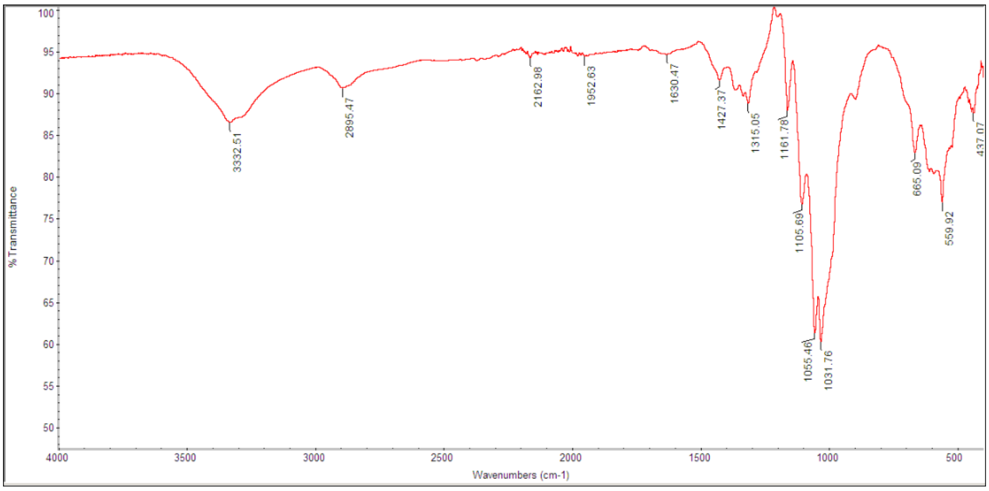

Figure S7. FT-IR spectrum of MCC-AmP-Au^0.

XPS spectra of materials and catalysts

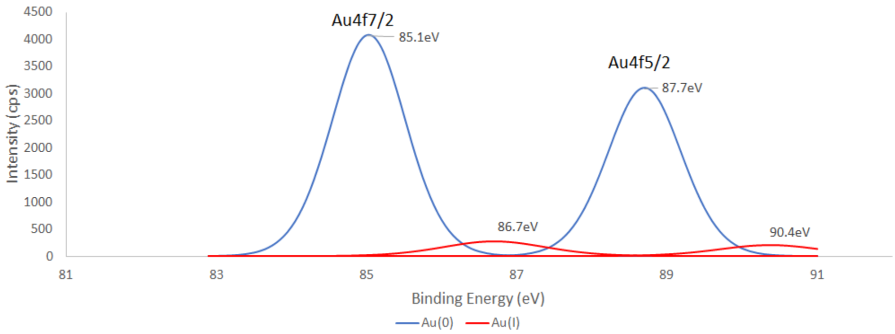

Figure S8. XPS spectrum of Au4f for MCC-McP-Au^0/Au^I.

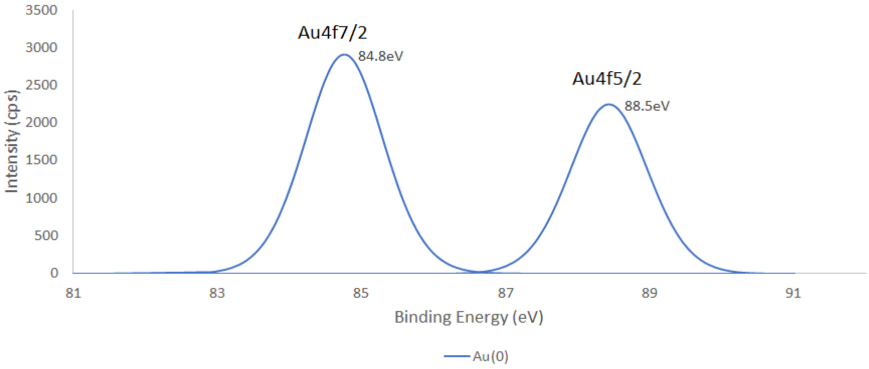

Figure S9. XPS spectrum of Au4f for MCC-McP-Au^0.

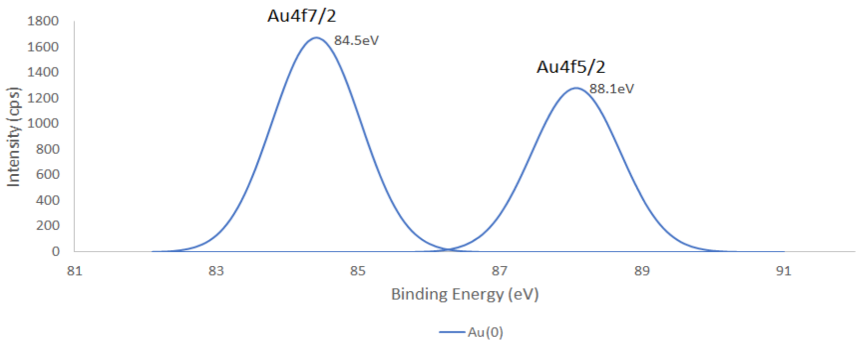

Figure S10. XPS spectrum of Au4f for MCC-McP-Au^0 recovered after reaction.

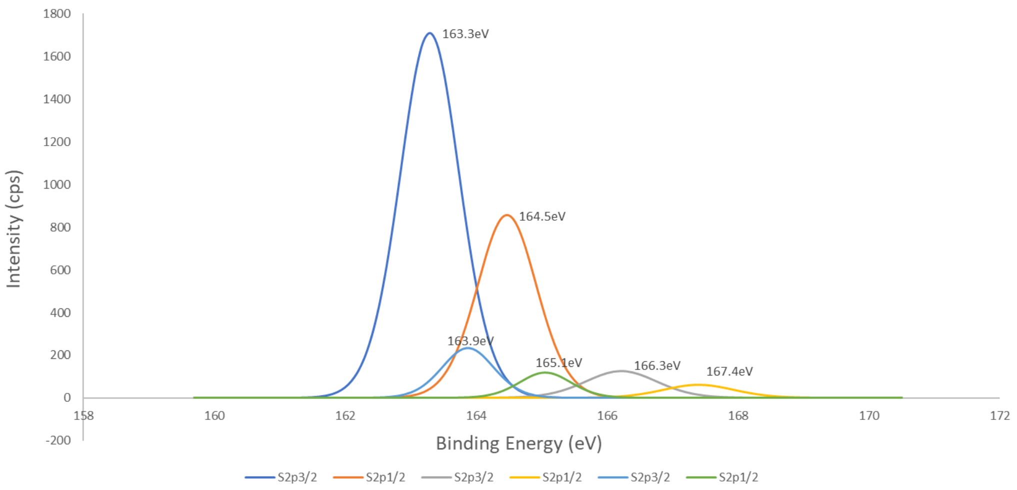

Figure S11. XPS spectrum of S2p for MCC- McP.

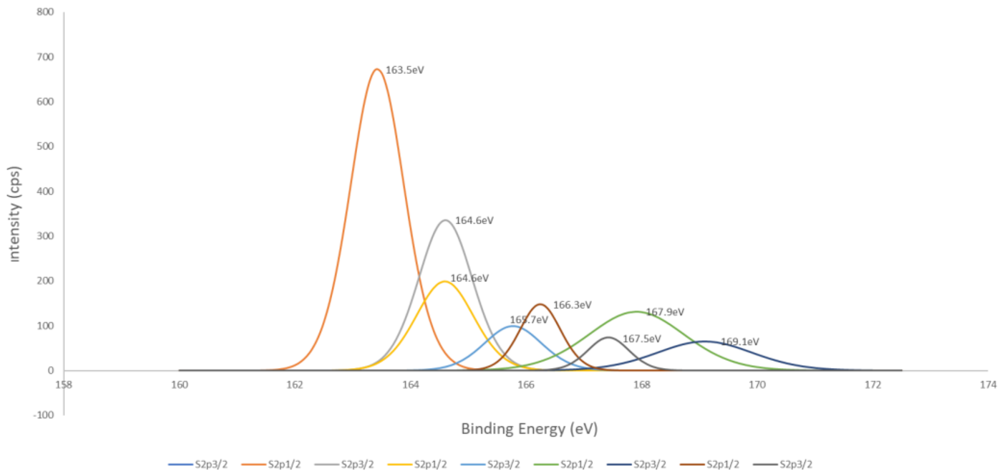

Figure S12. XPS spectrum of S2p for MCC-McP-Au^0/Au^I.

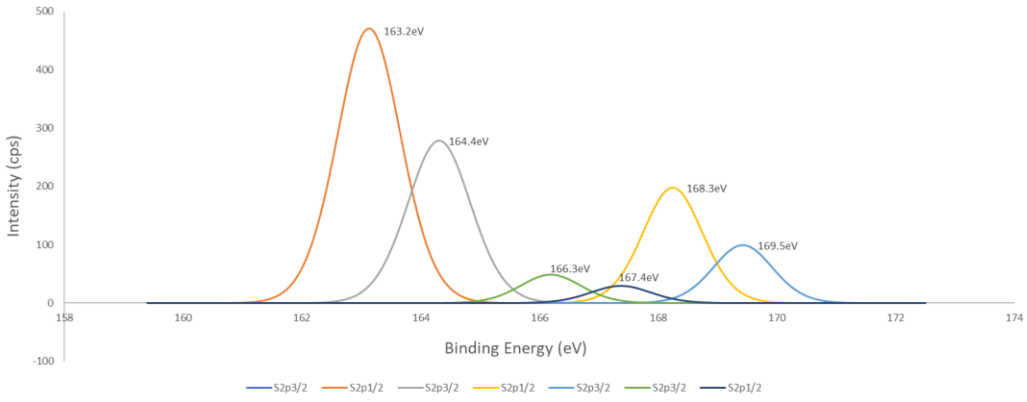

Figure S13. XPS spectrum of S2p for MCC-McP-Au^0.

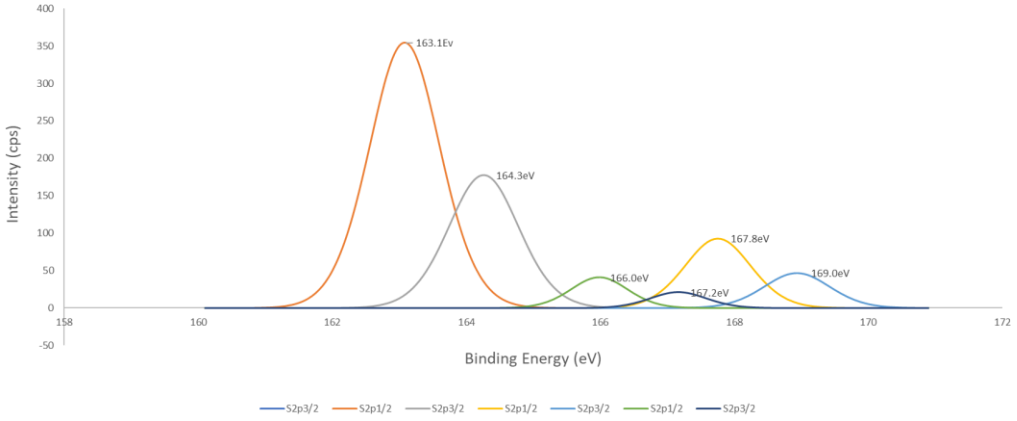

Figure S14. XPS spectrum of S2p for MCC-McP-Au^0 recovered after reaction.

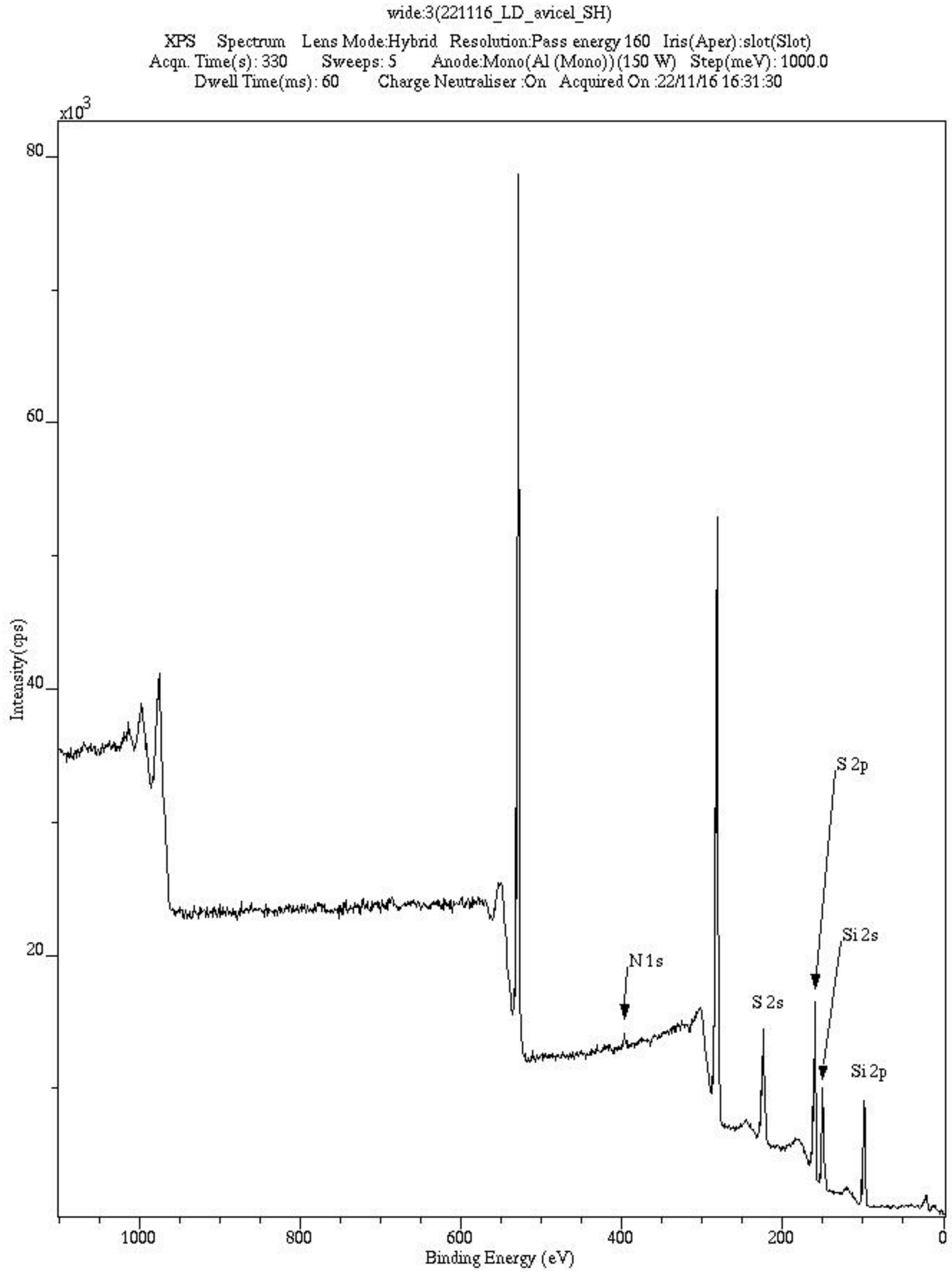

Figure S15. XPS spectrum for MCC-McP.

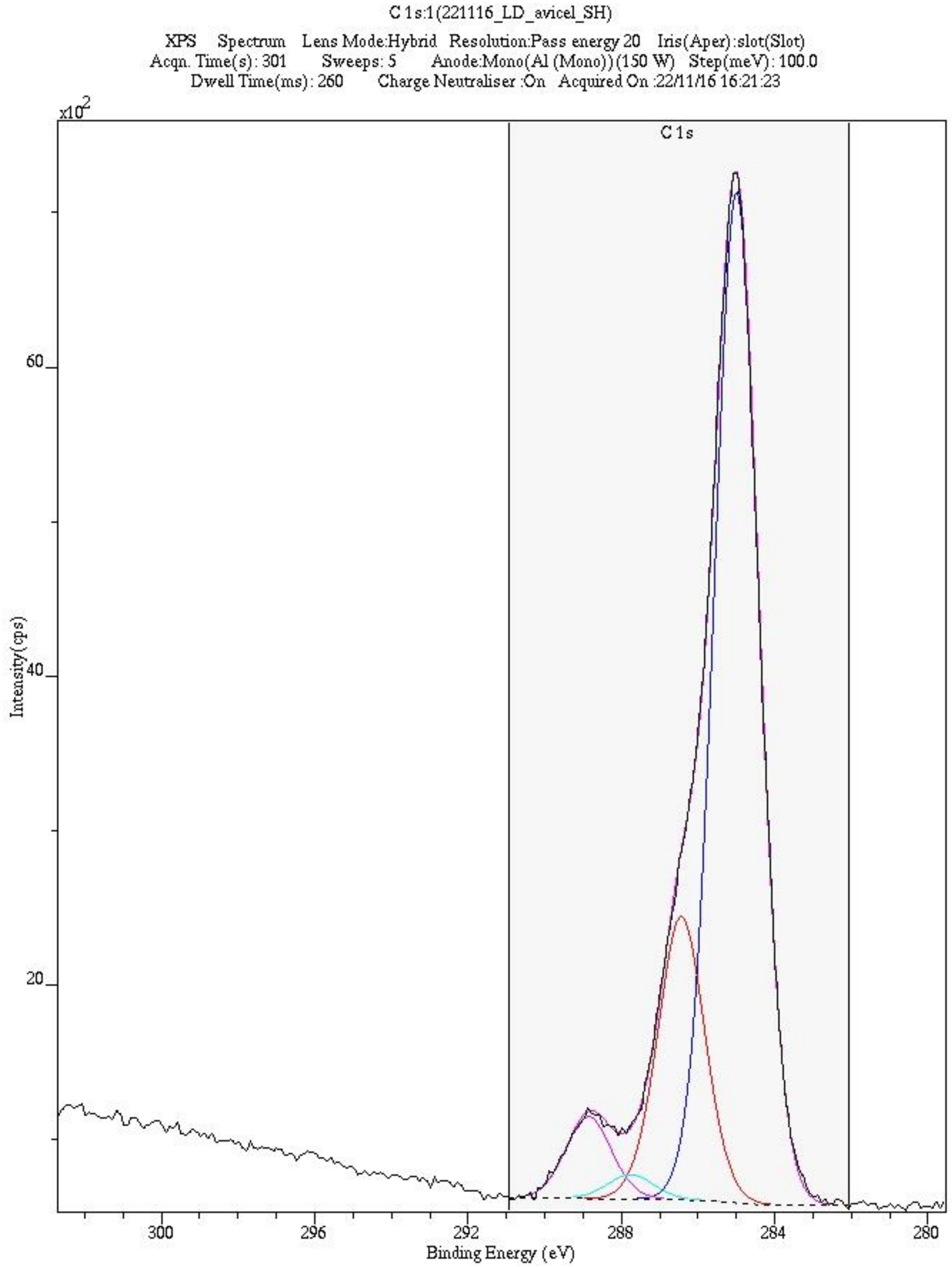

Figure S16. XPS spectrum of C1s for MCC-McP.

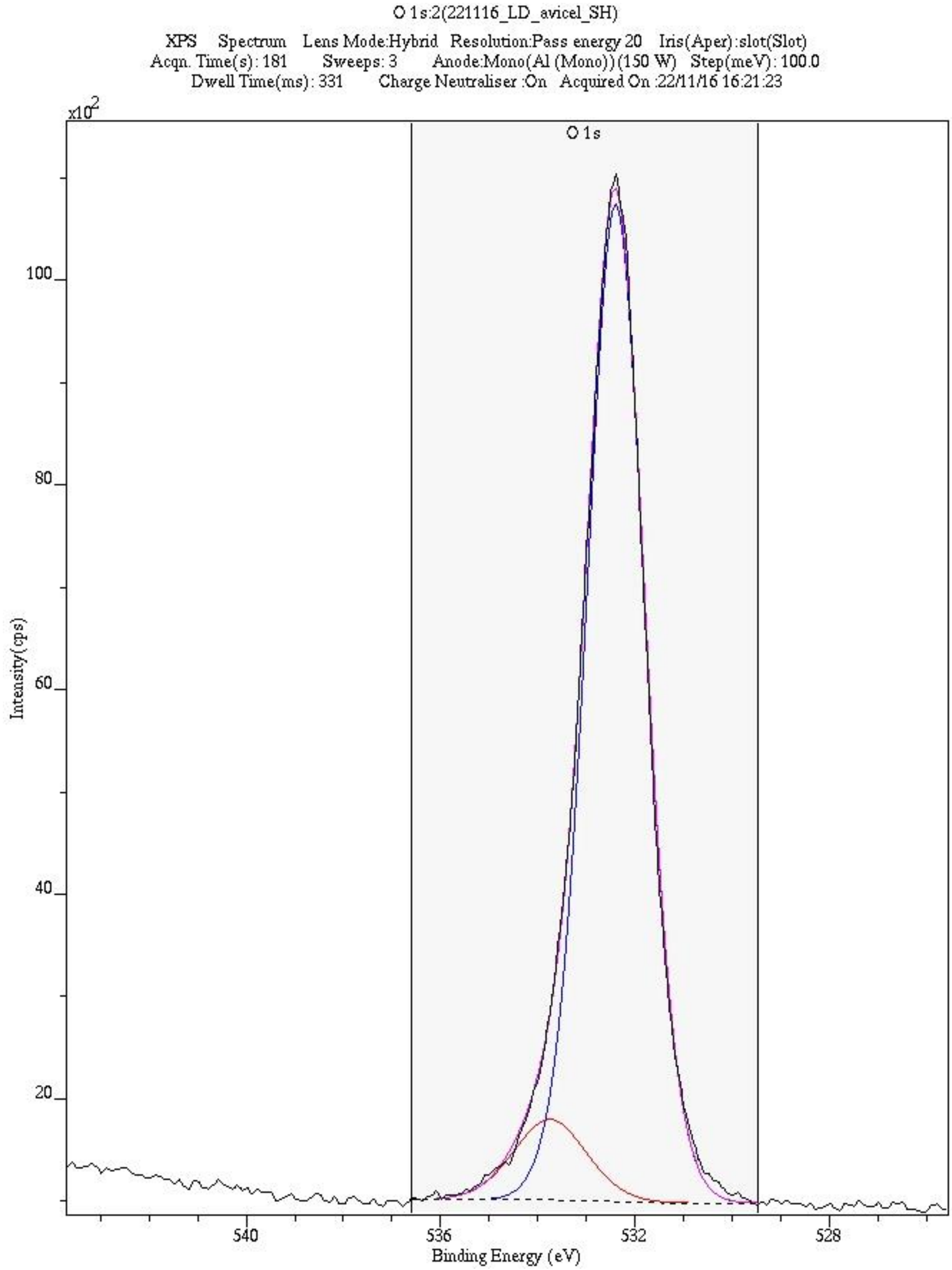

Figure S17. XPS spectrum of O1s for MCC-McP.

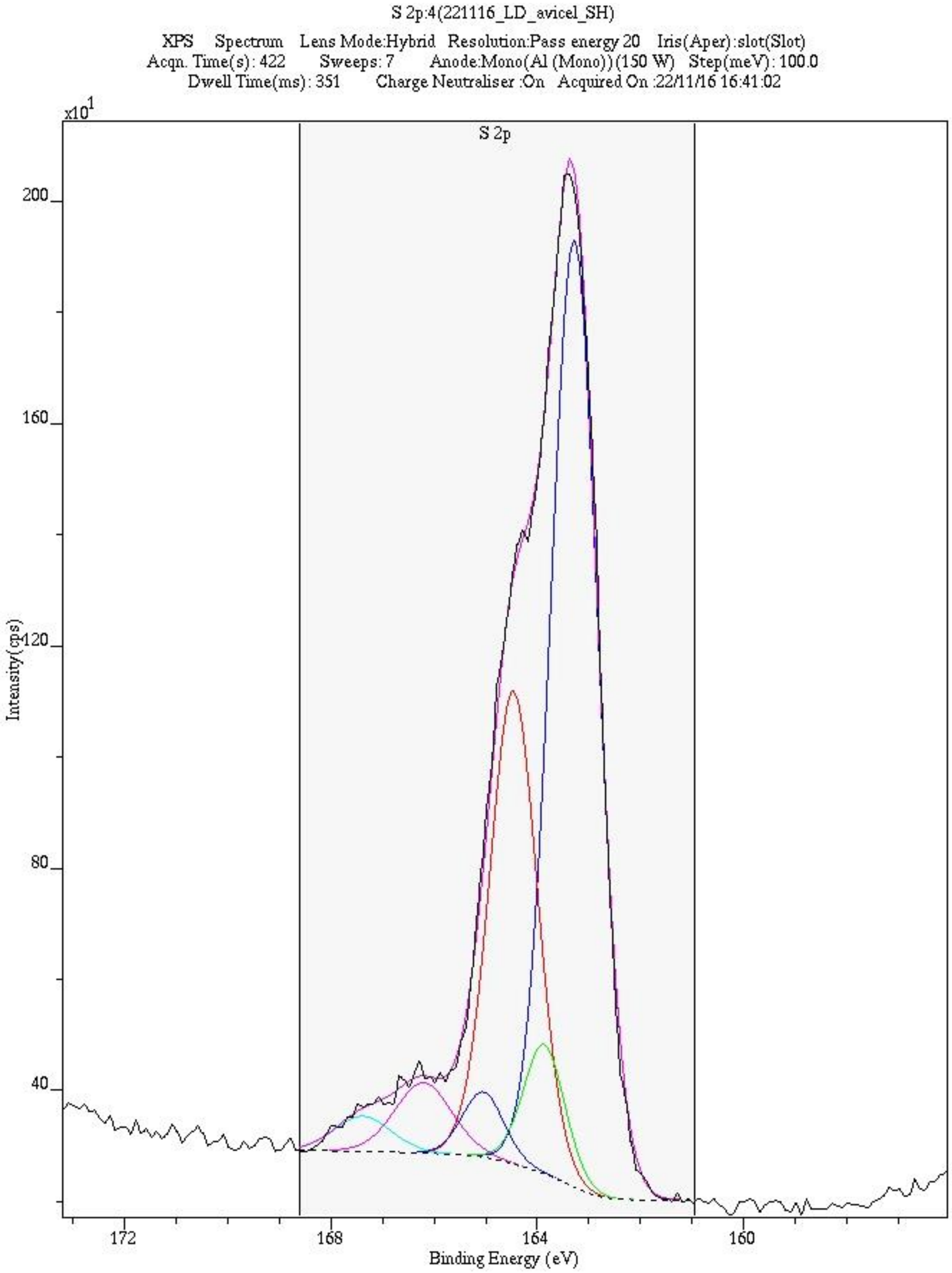

Figure S18. XPS spectrum of S2p for MCC-McP.

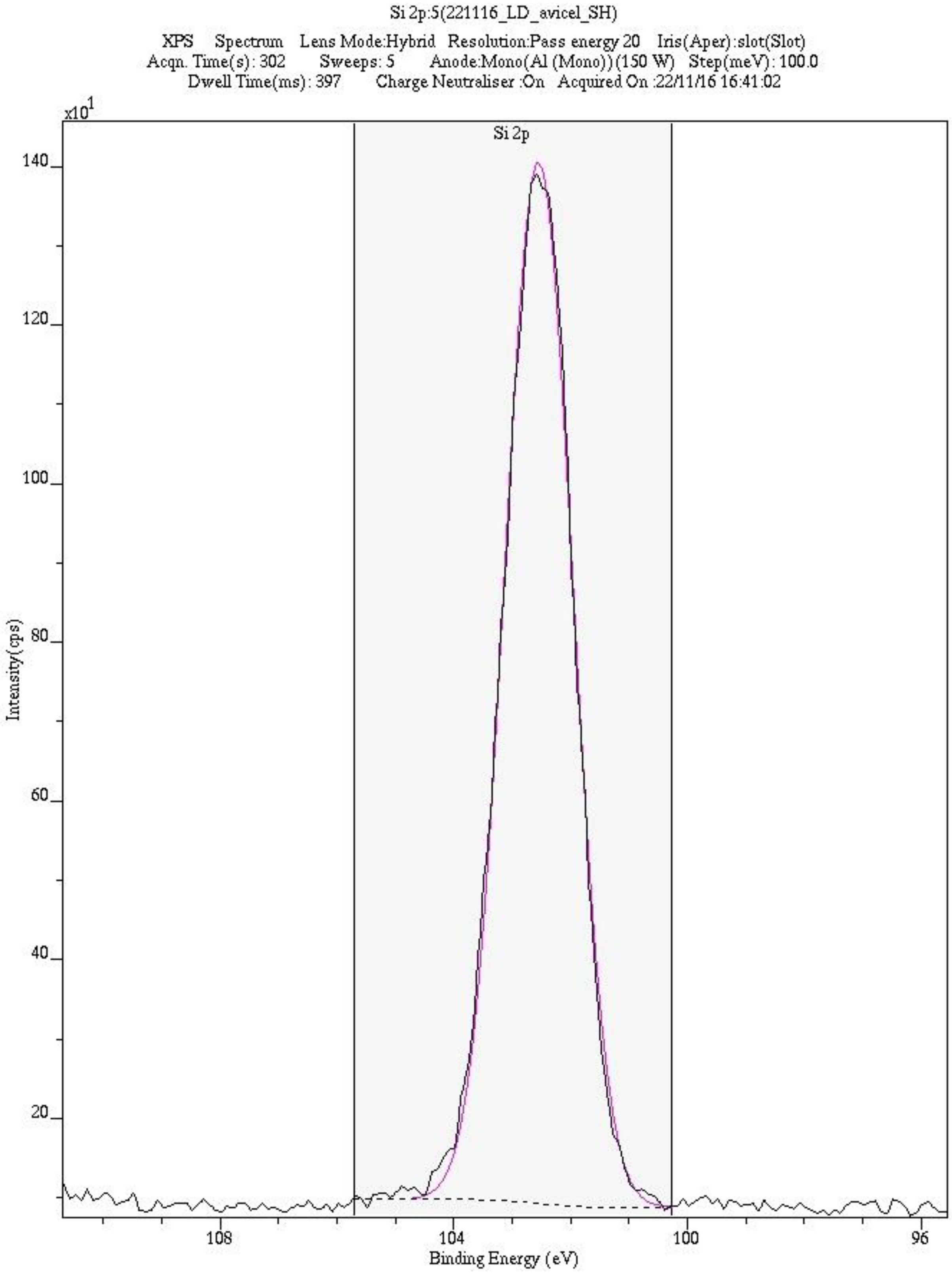

Figure S19. XPS spectrum of Si2p for MCC-McP.

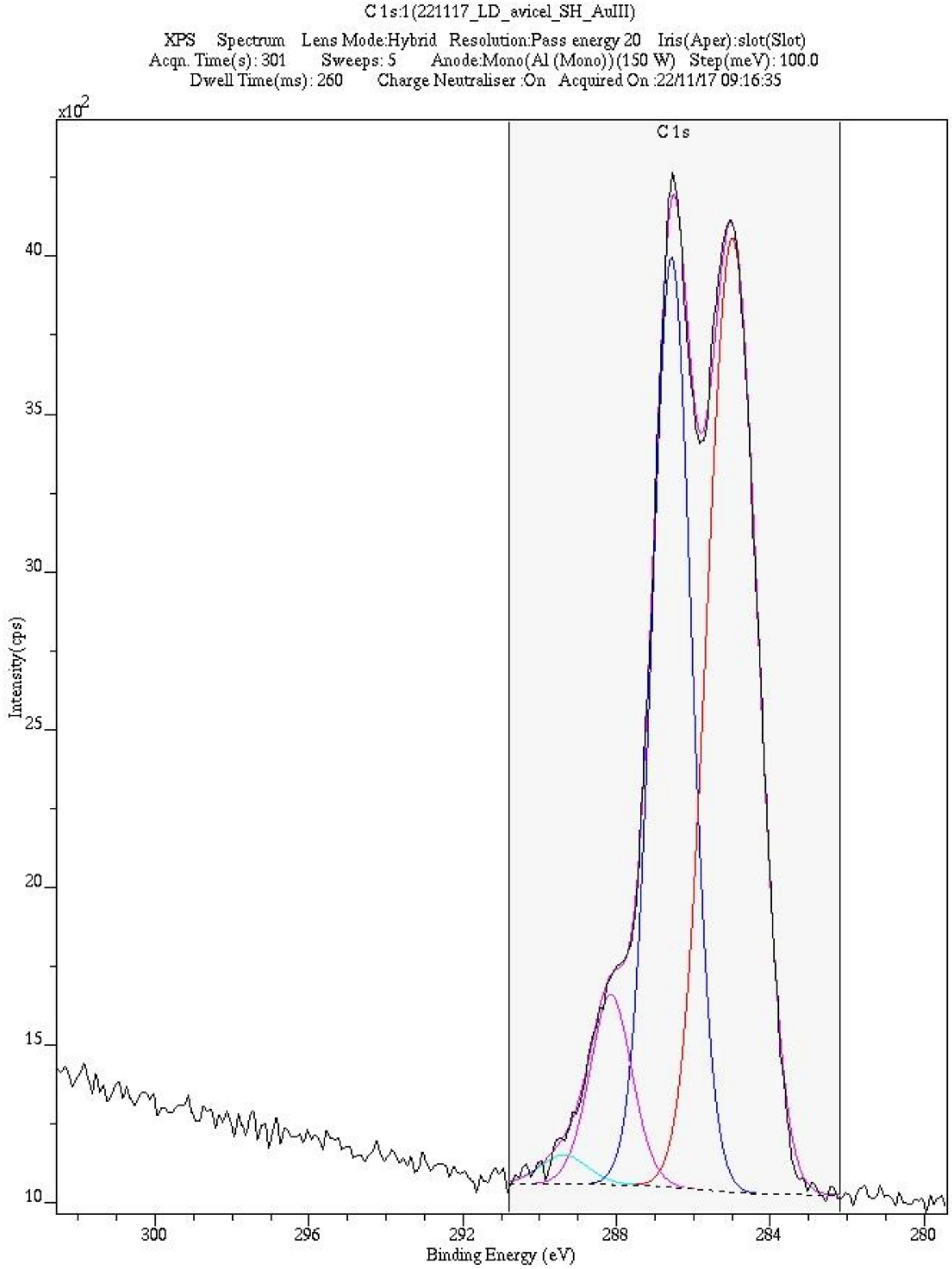

Figure S20. XPS spectrum of Si2p for MCC-McP.

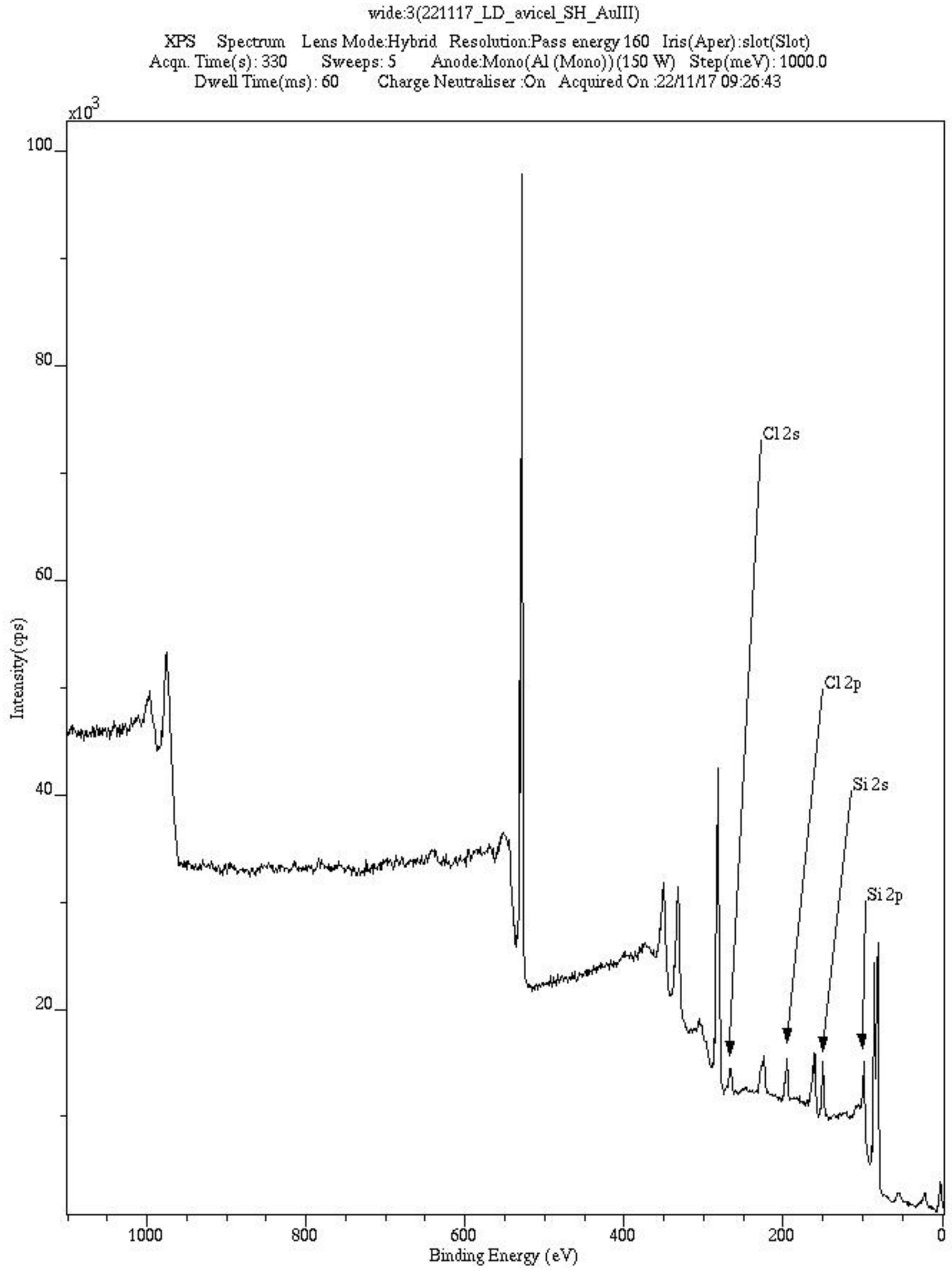

Figure S21. XPS spectrum for MCC-McP-Au^0/Au^I

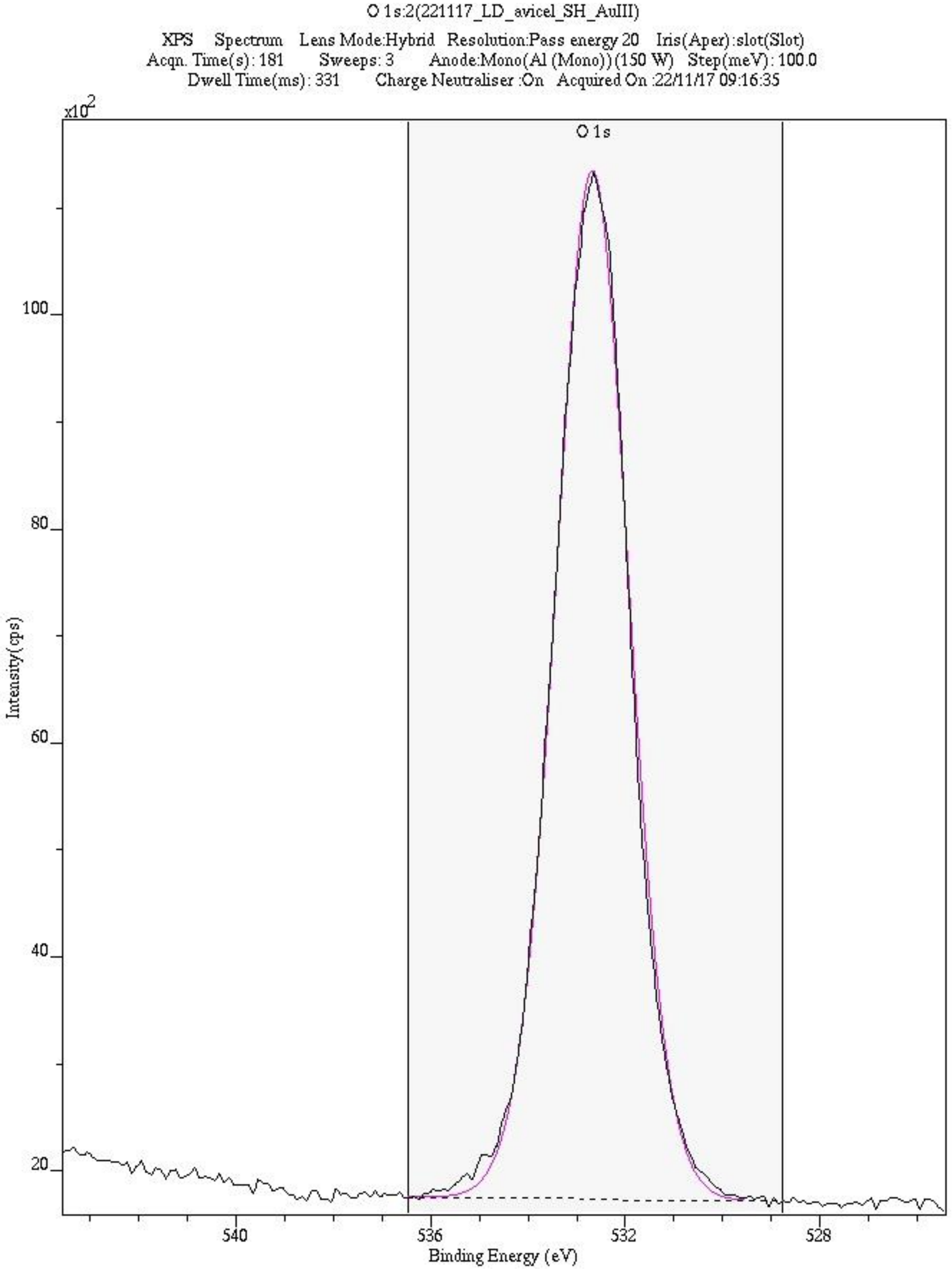

Figure S22. XPS spectrum of O1s for MCC-McP- Au^0/Au^I

.

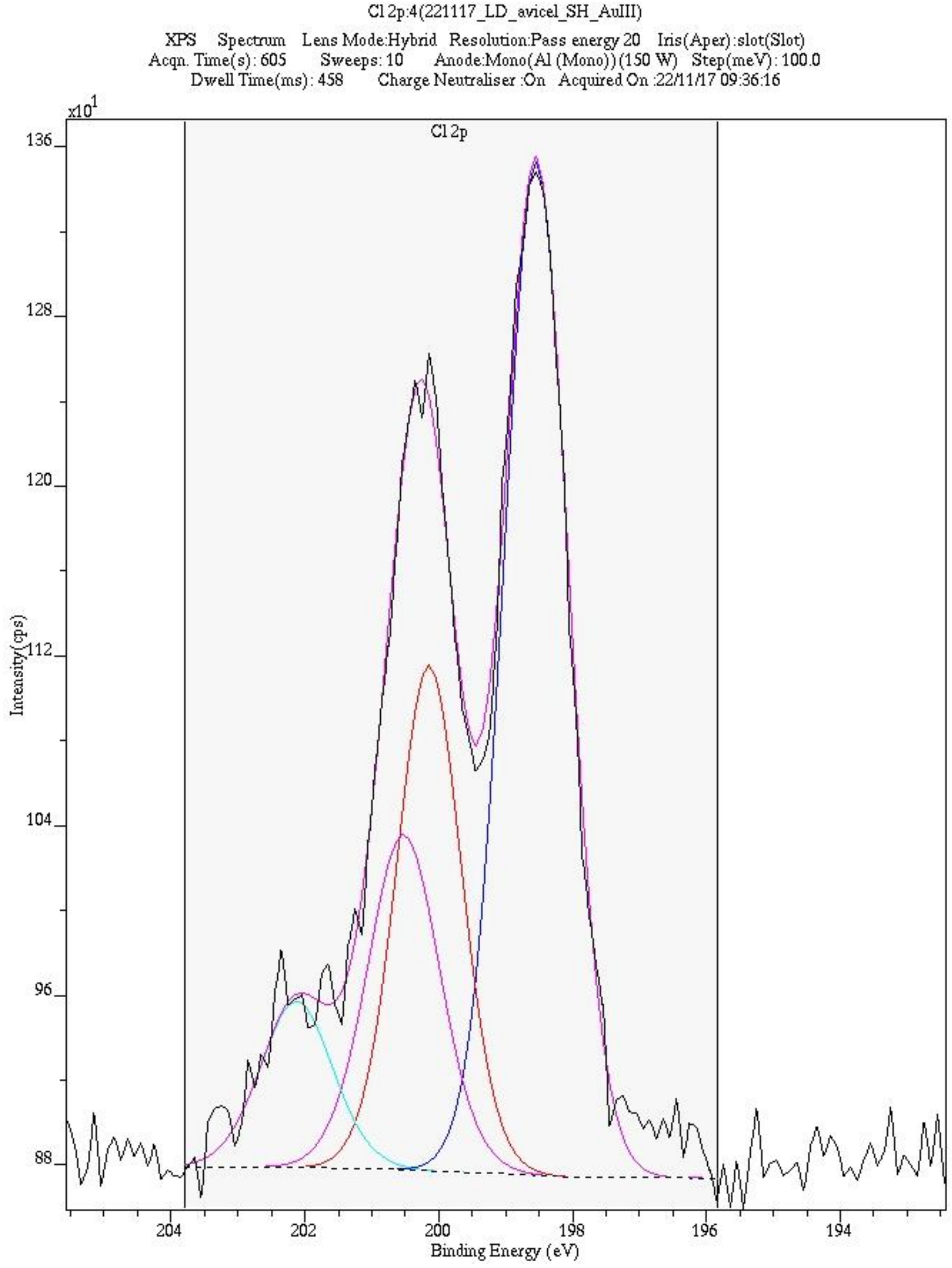

Figure S23. XPS spectrum of Cl2p for MCC-McP-Au^0/Au^I

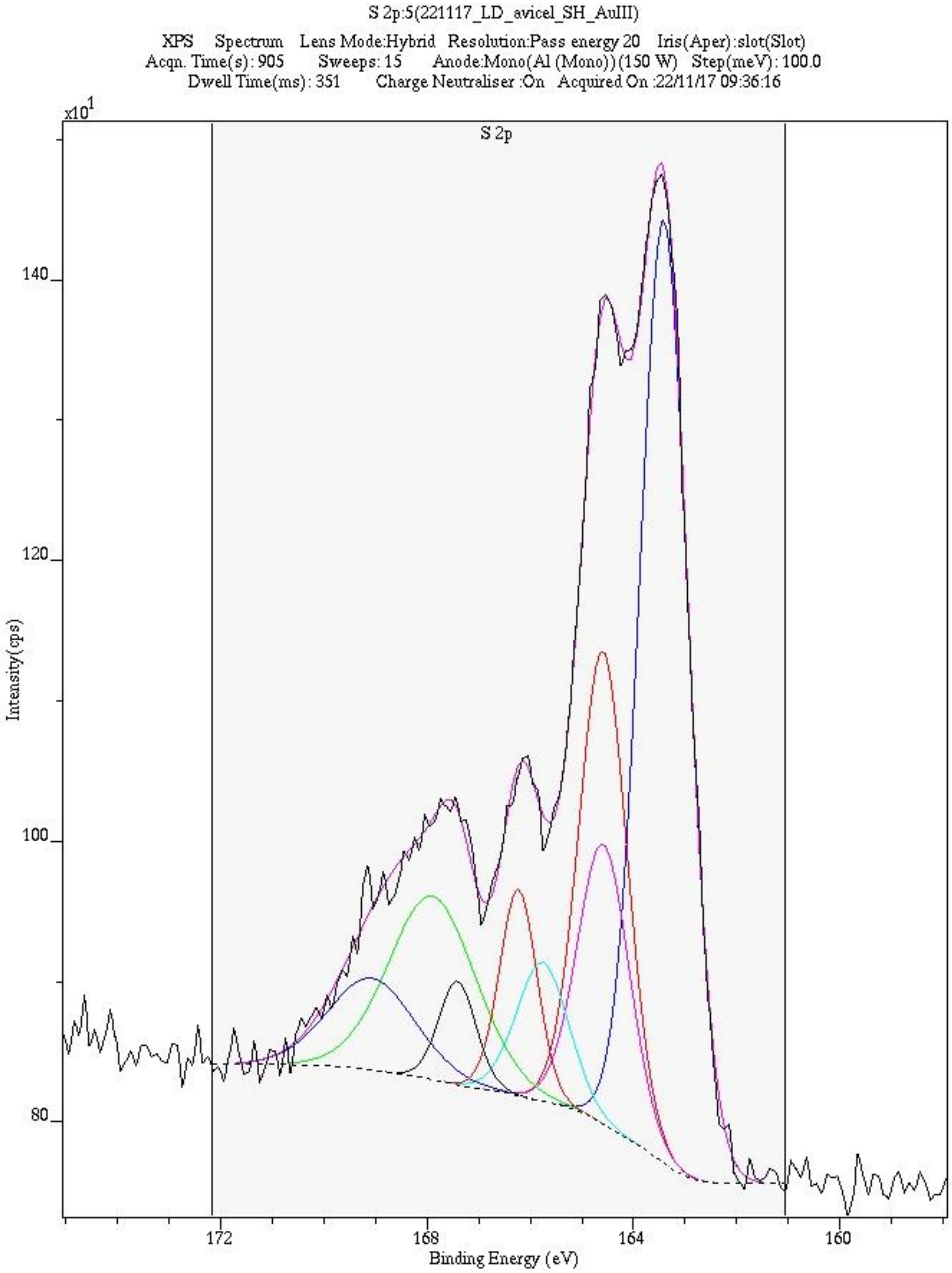

Figure S24. XPS spectrum of S2p for MCC-McP-Au^0/Au^I

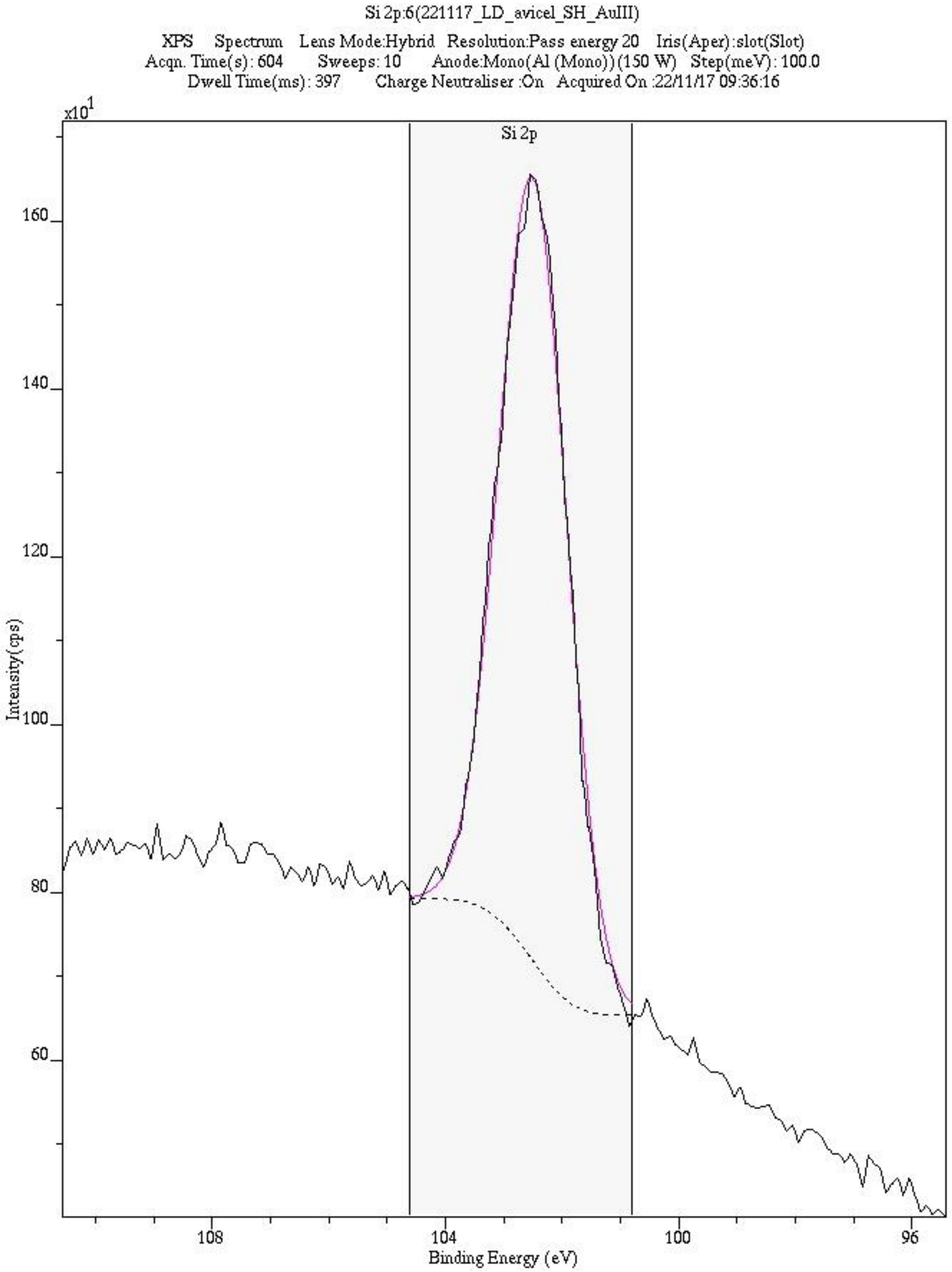

Figure S25. XPS spectrum of Si2p for MCC-McP- Au^0/Au^I

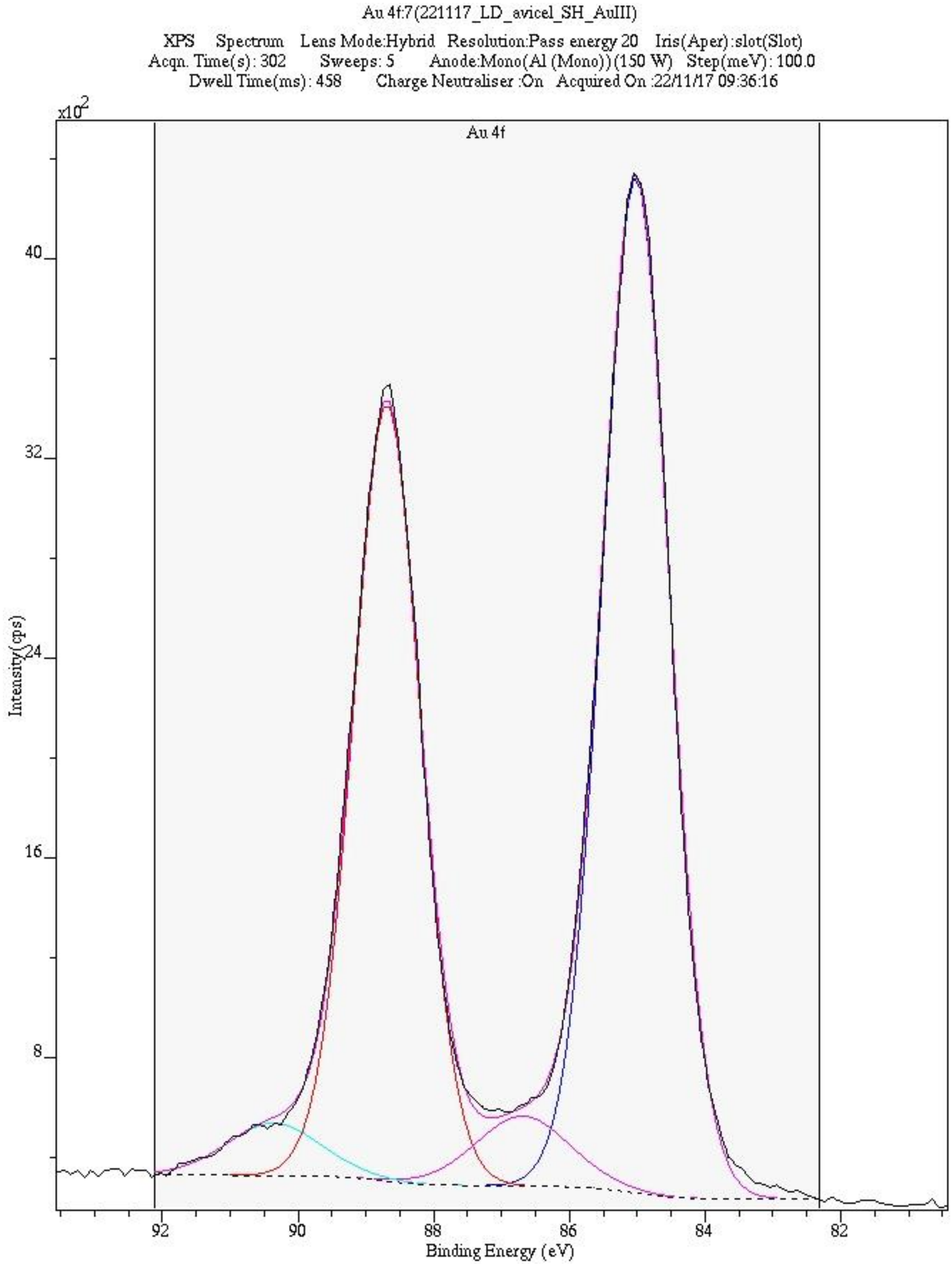

Figure S26. XPS spectrum of Au4f for MCC-McP- Au^0/Au^I

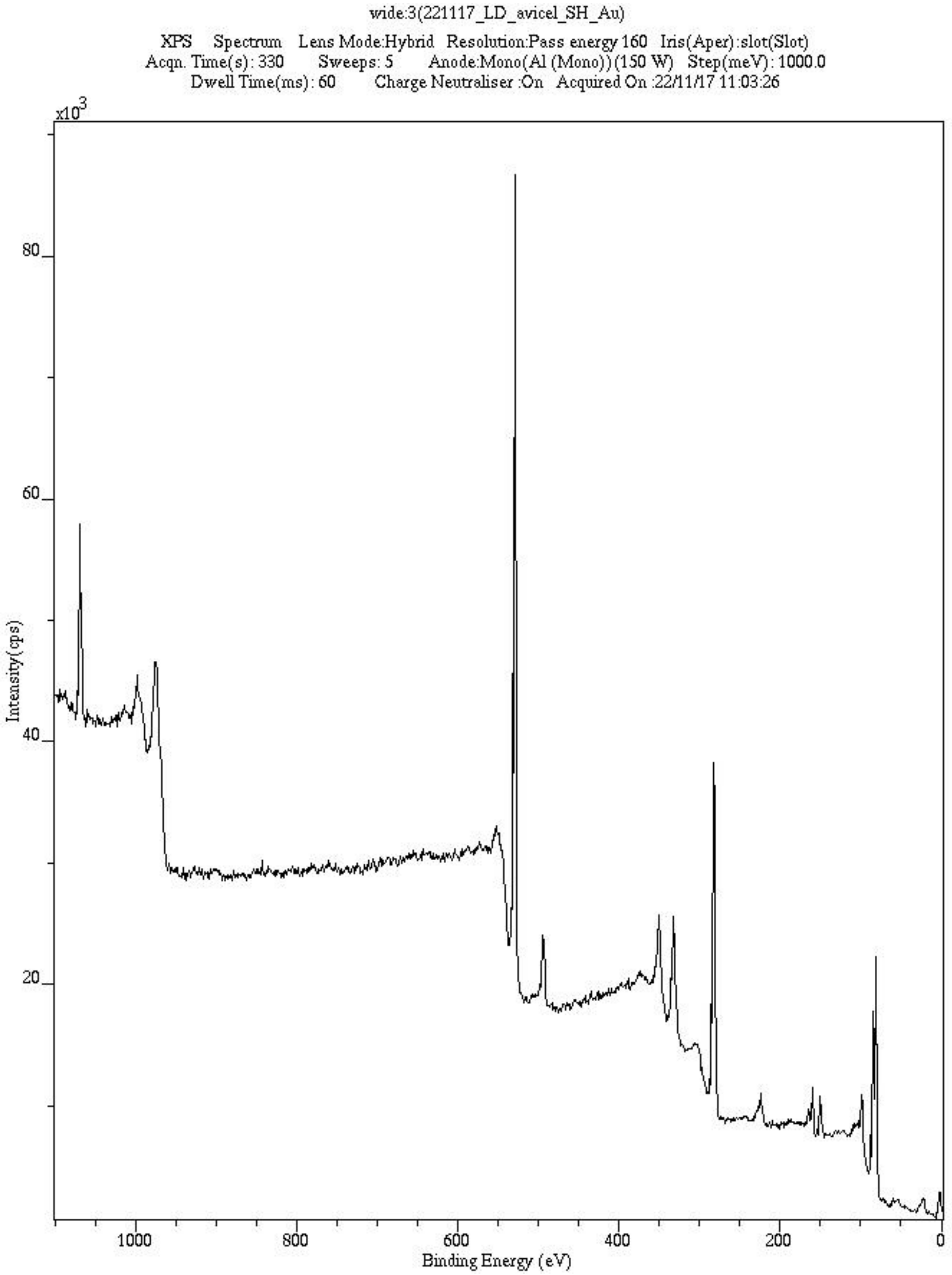

Figure S27. XPS spectrum for MCC-McP-Au^0.

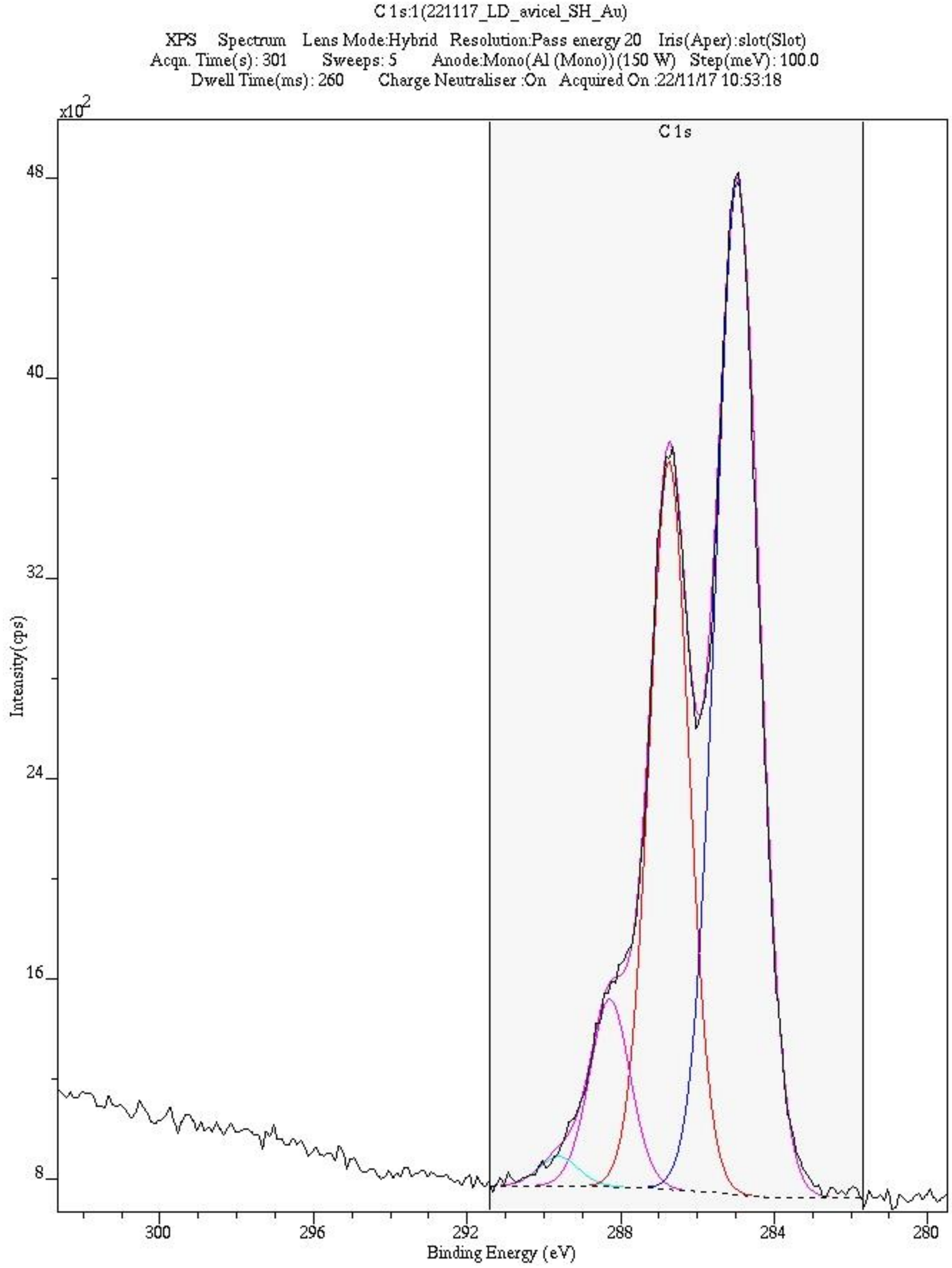

Figure S28. XPS spectrum of C1S for MCC-McP-Au^0.

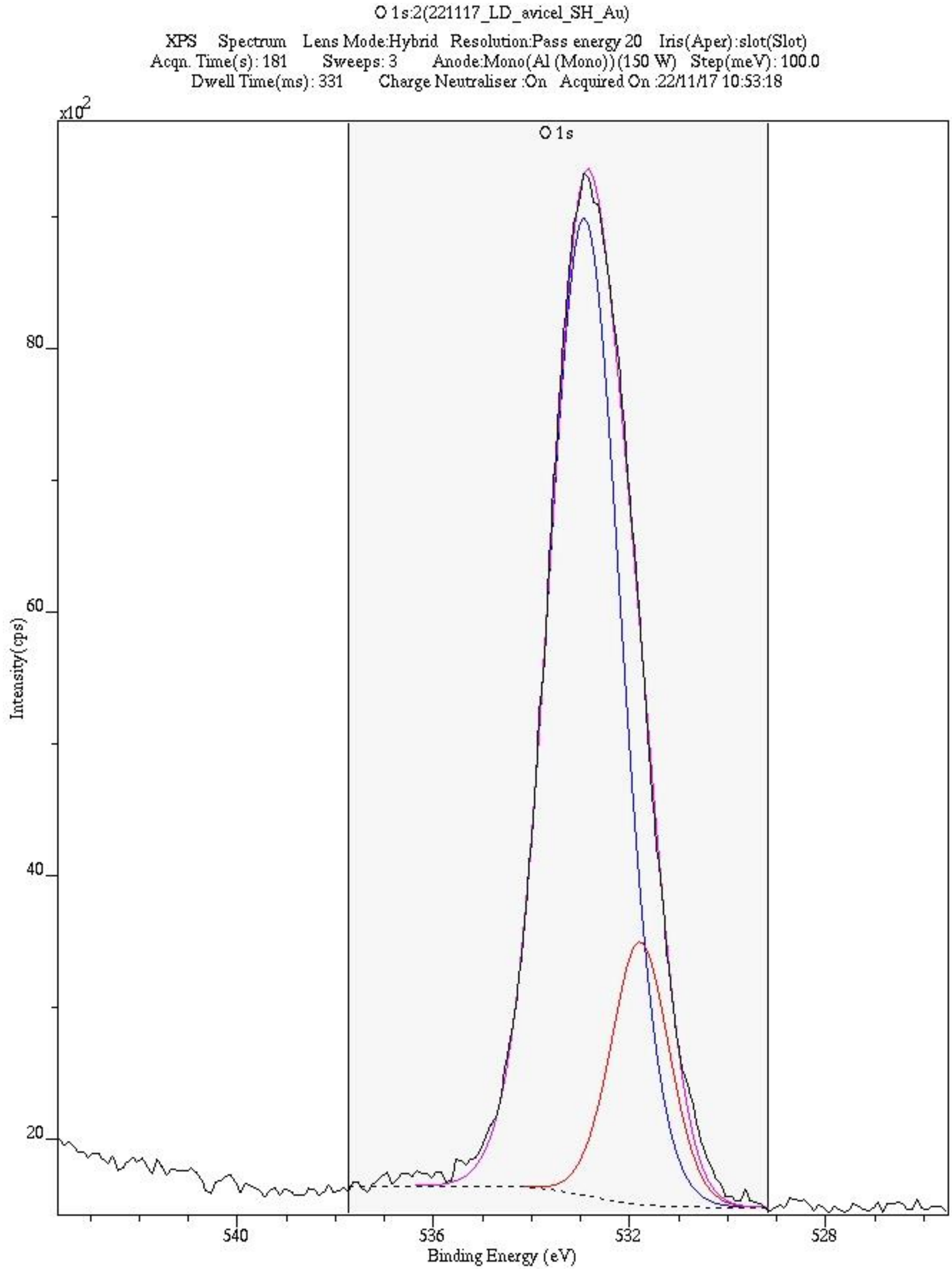

Figure S29. XPS spectrum of O1S for MCC-McP-Au^0.

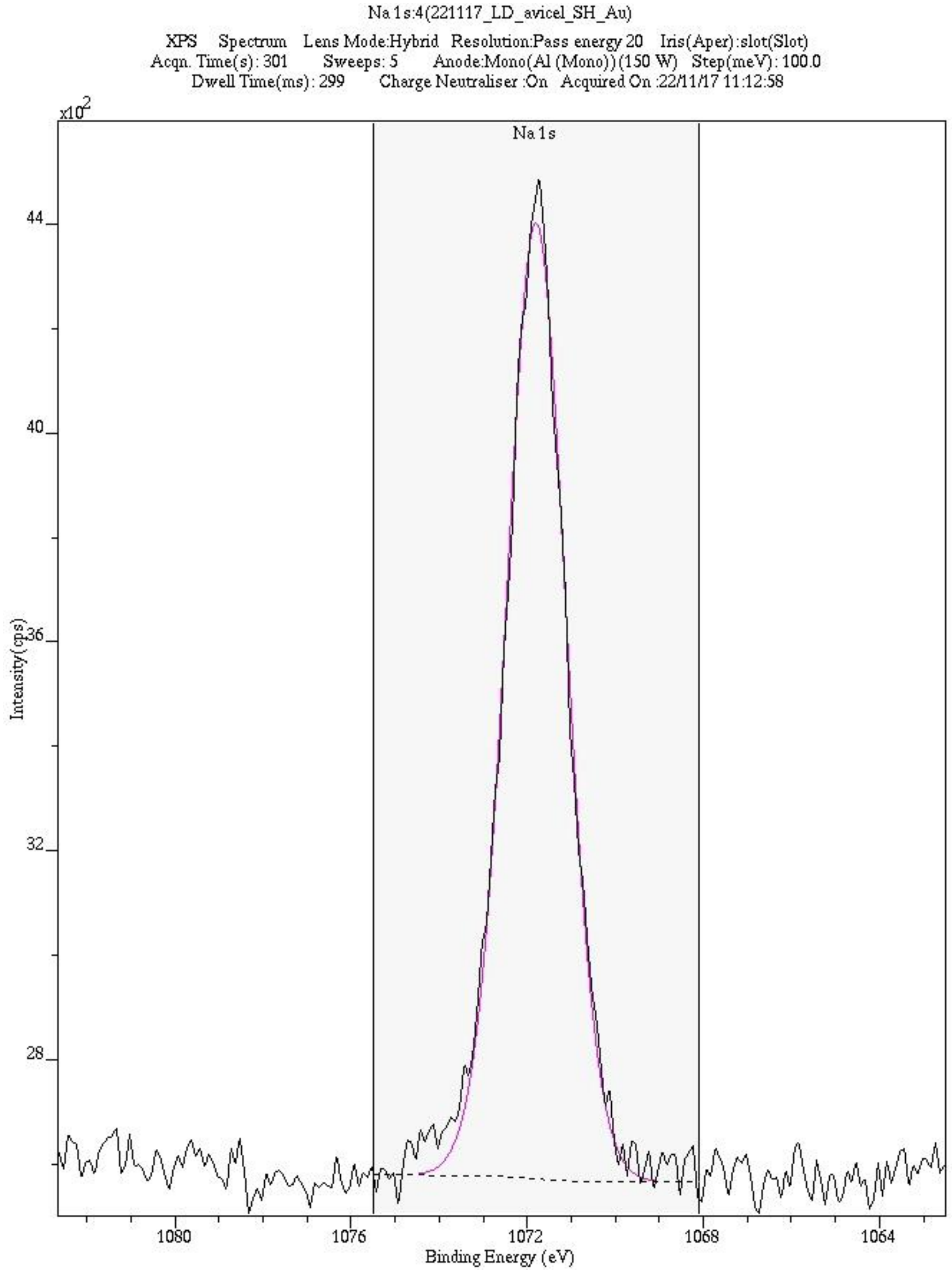

Figure S30. XPS spectrum of Na1S for MCC-McP-Au^0.

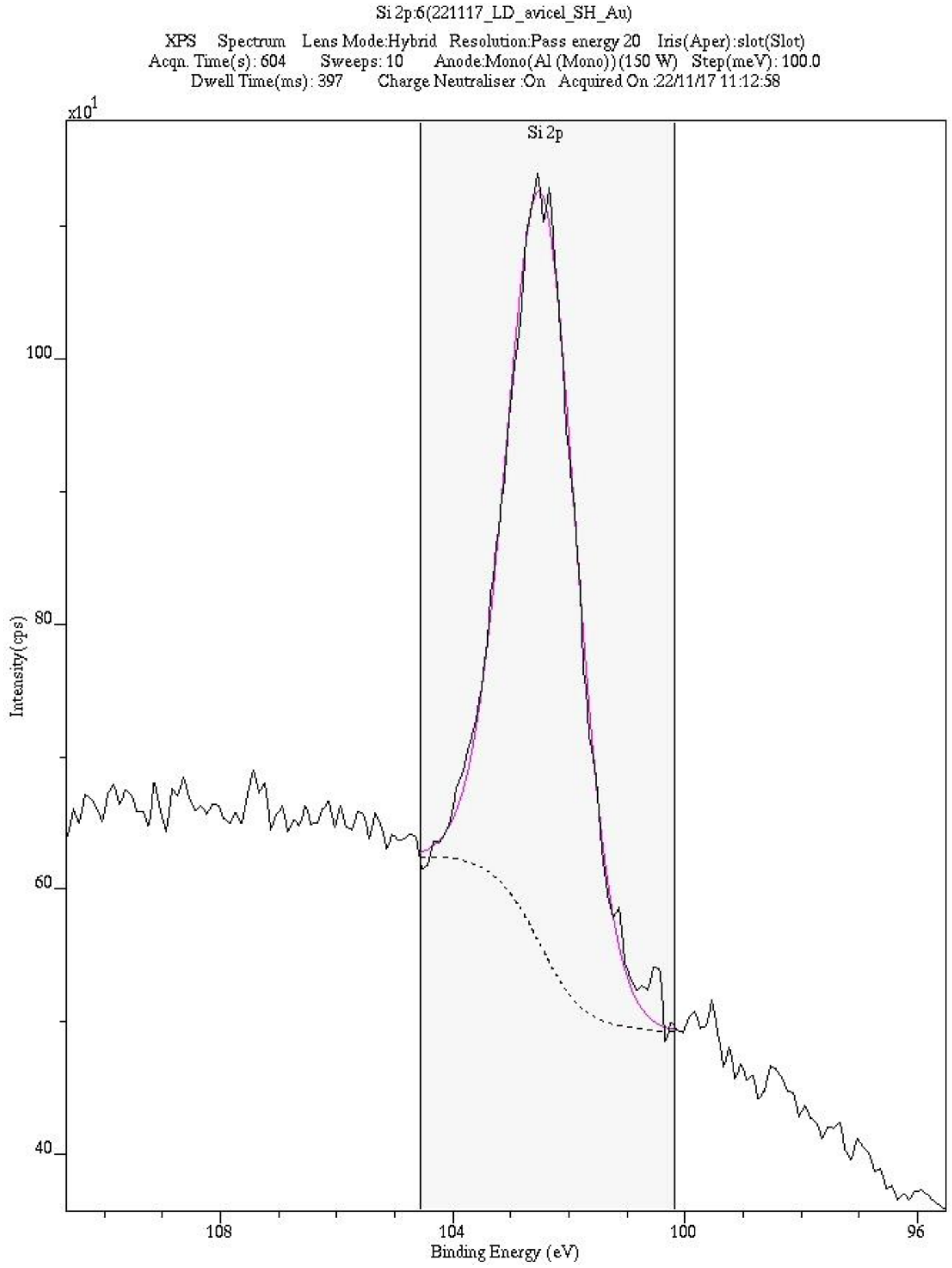

Figure S31. XPS spectrum of Si2p for MCC-McP-Au^0.

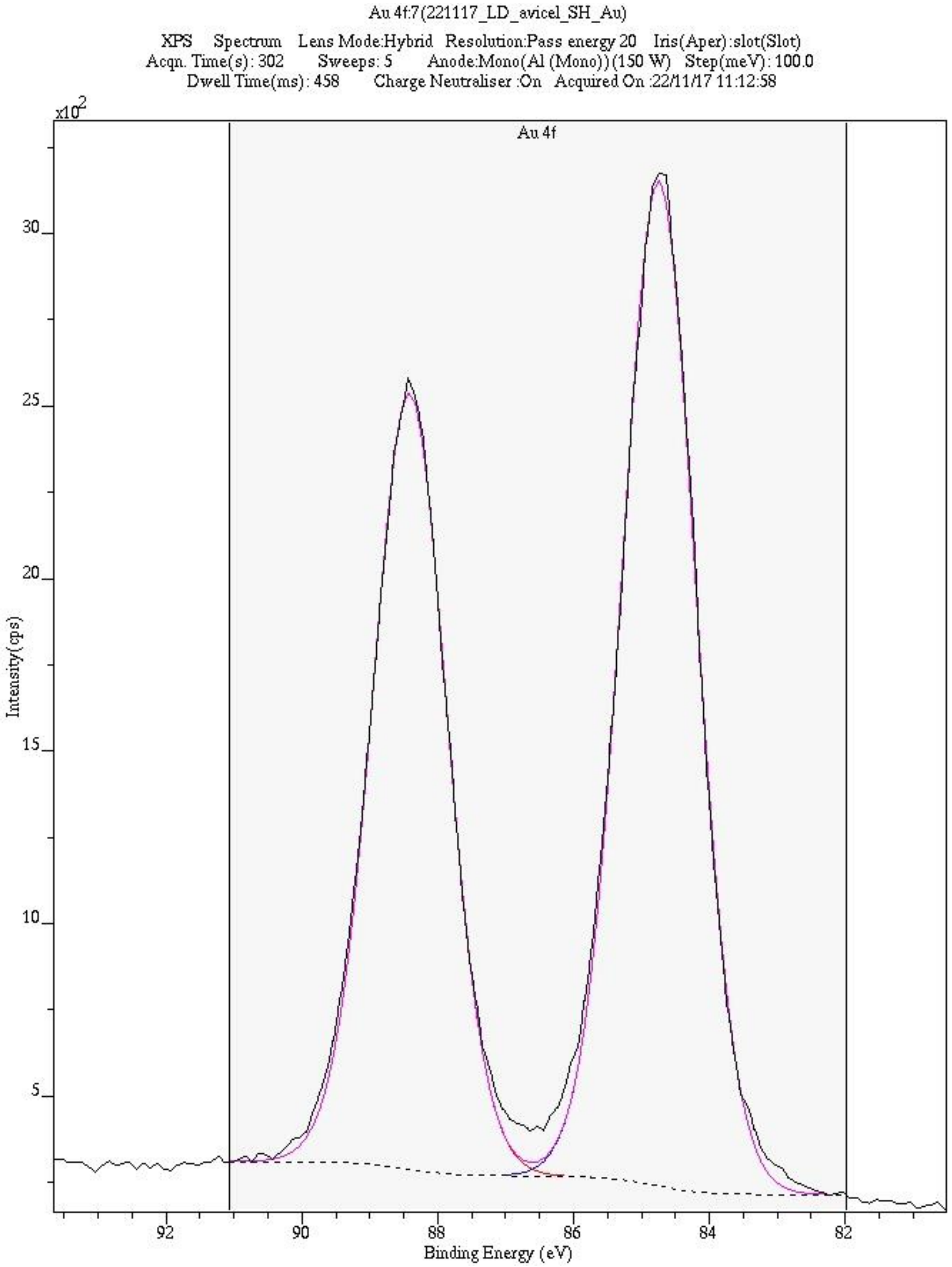

Figure S32. XPS spectrum of Au4f for MCC-McP-Au^0.

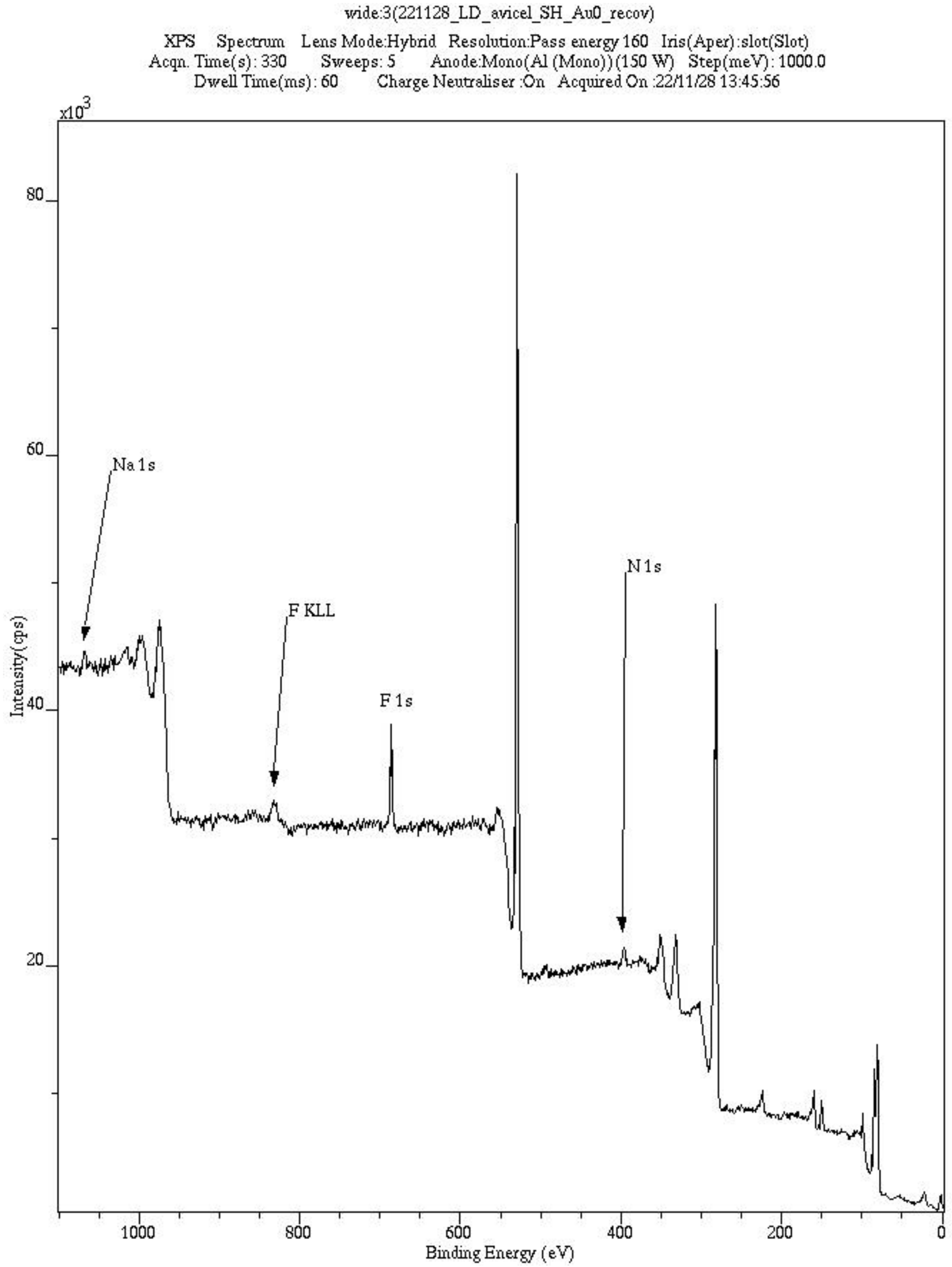

Figure S33. XPS spectrum for MCC-McP-Au^0 recycled after reaction.

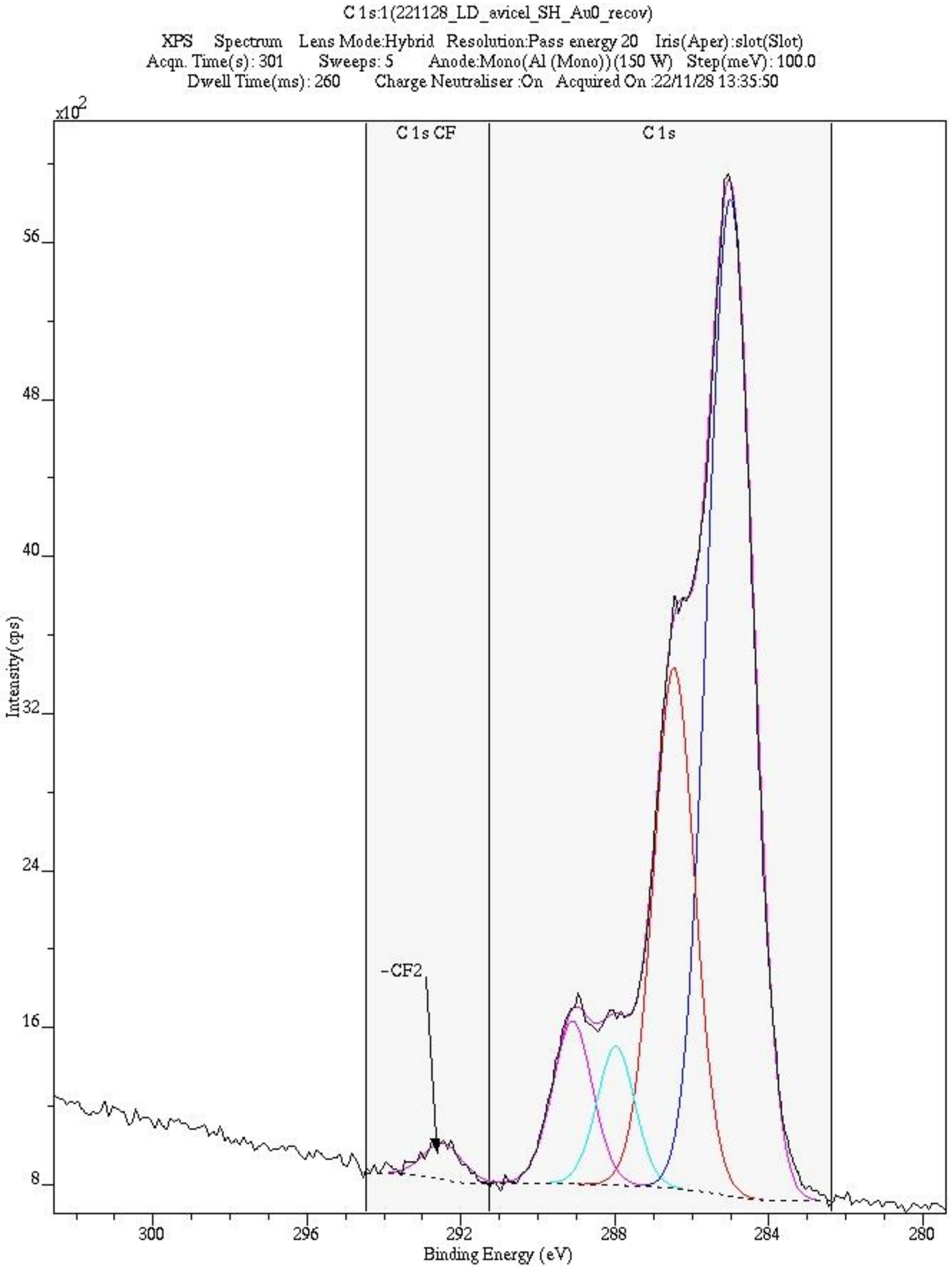

Figure S34. XPS spectrum of C1s for MCC-McP-Au^0 recycled after reaction.

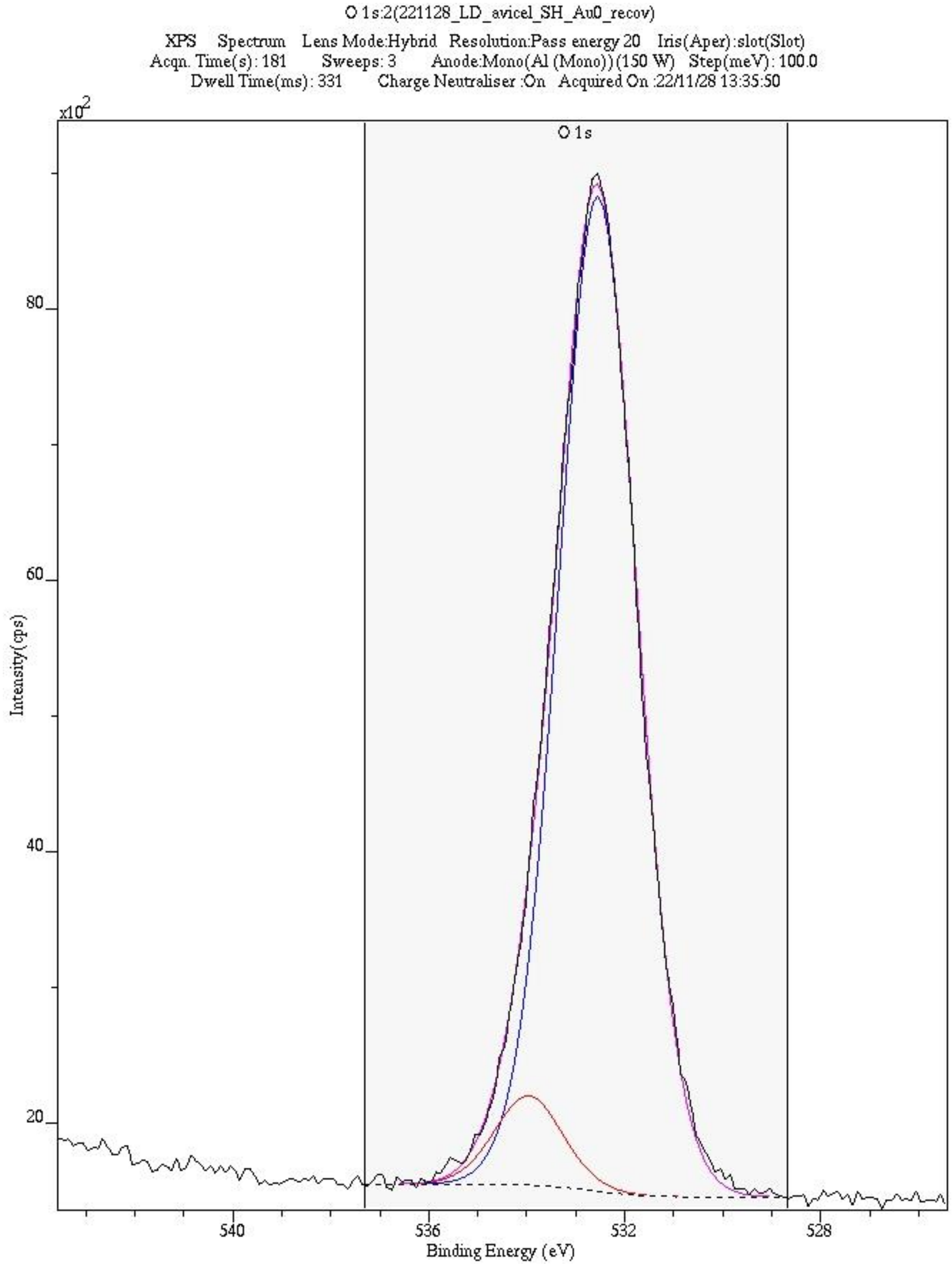

Figure S35. XPS spectrum of O1s for MCC-McP-Au^0 recycled after reaction.

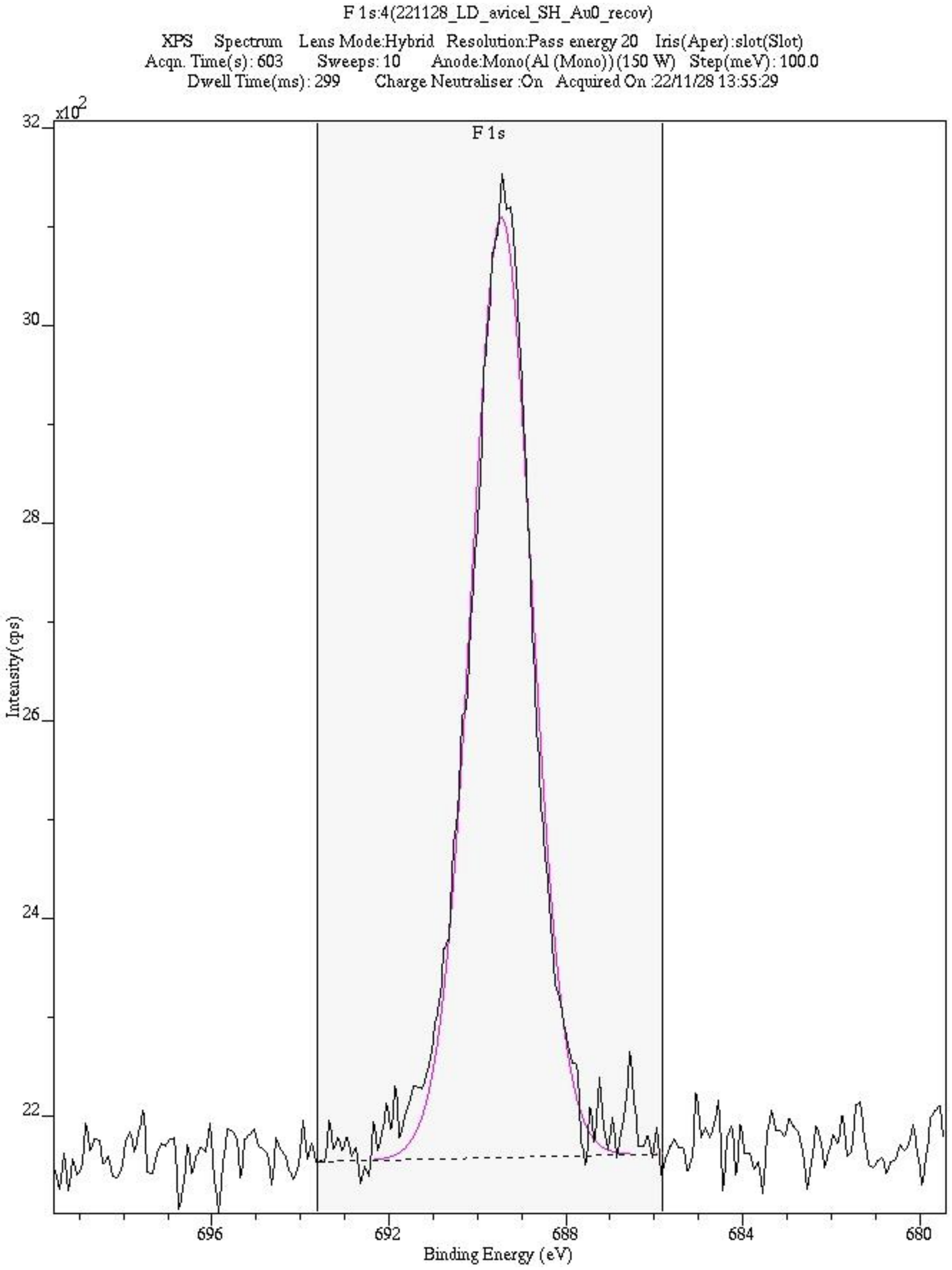

Figure S36. XPS spectrum of F1s for MCC-McP-Au^0 recycled after reaction.

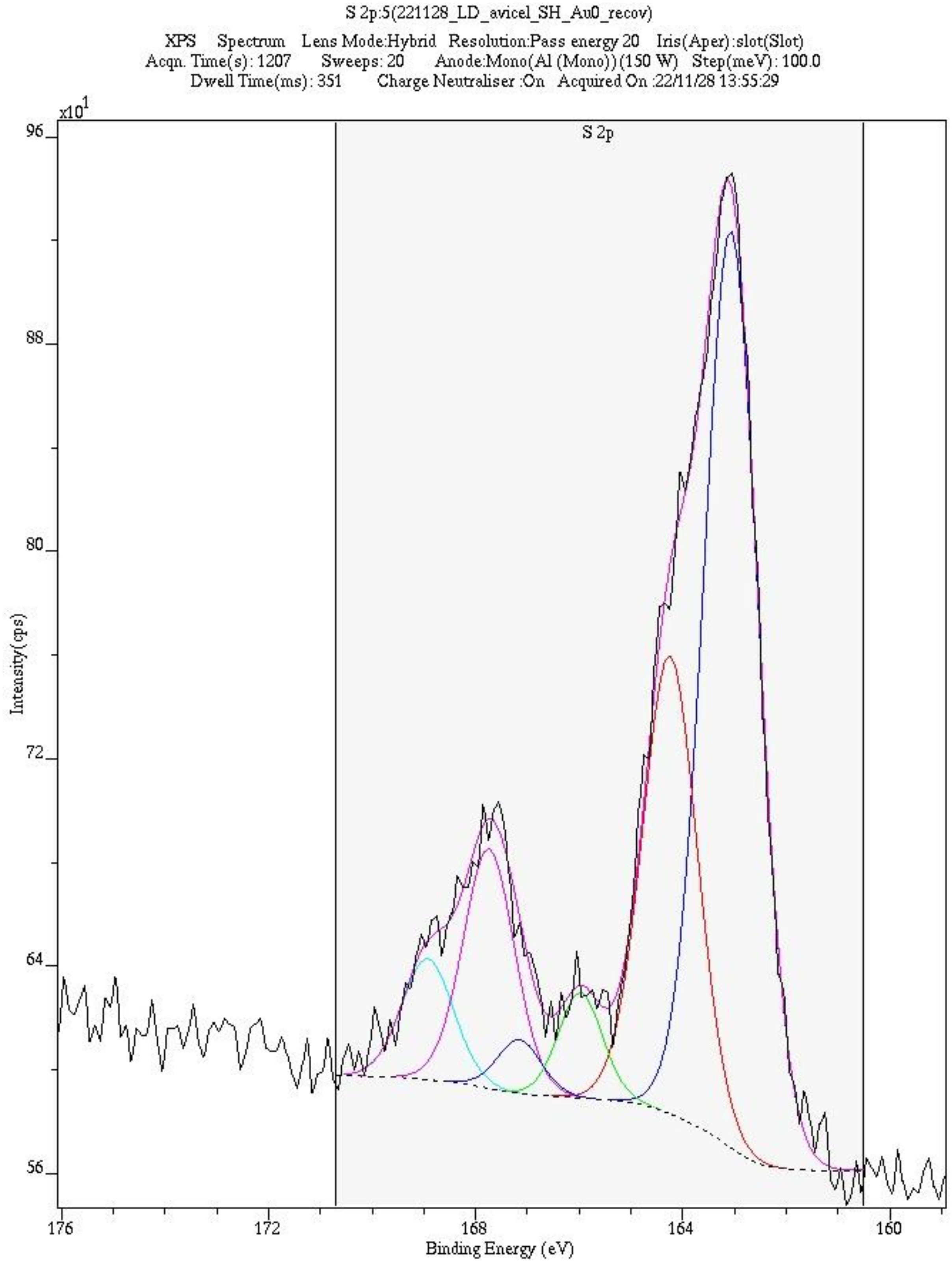

Figure S37. XPS spectrum of S2p for MCC-McP-Au^0 recycled after reaction.

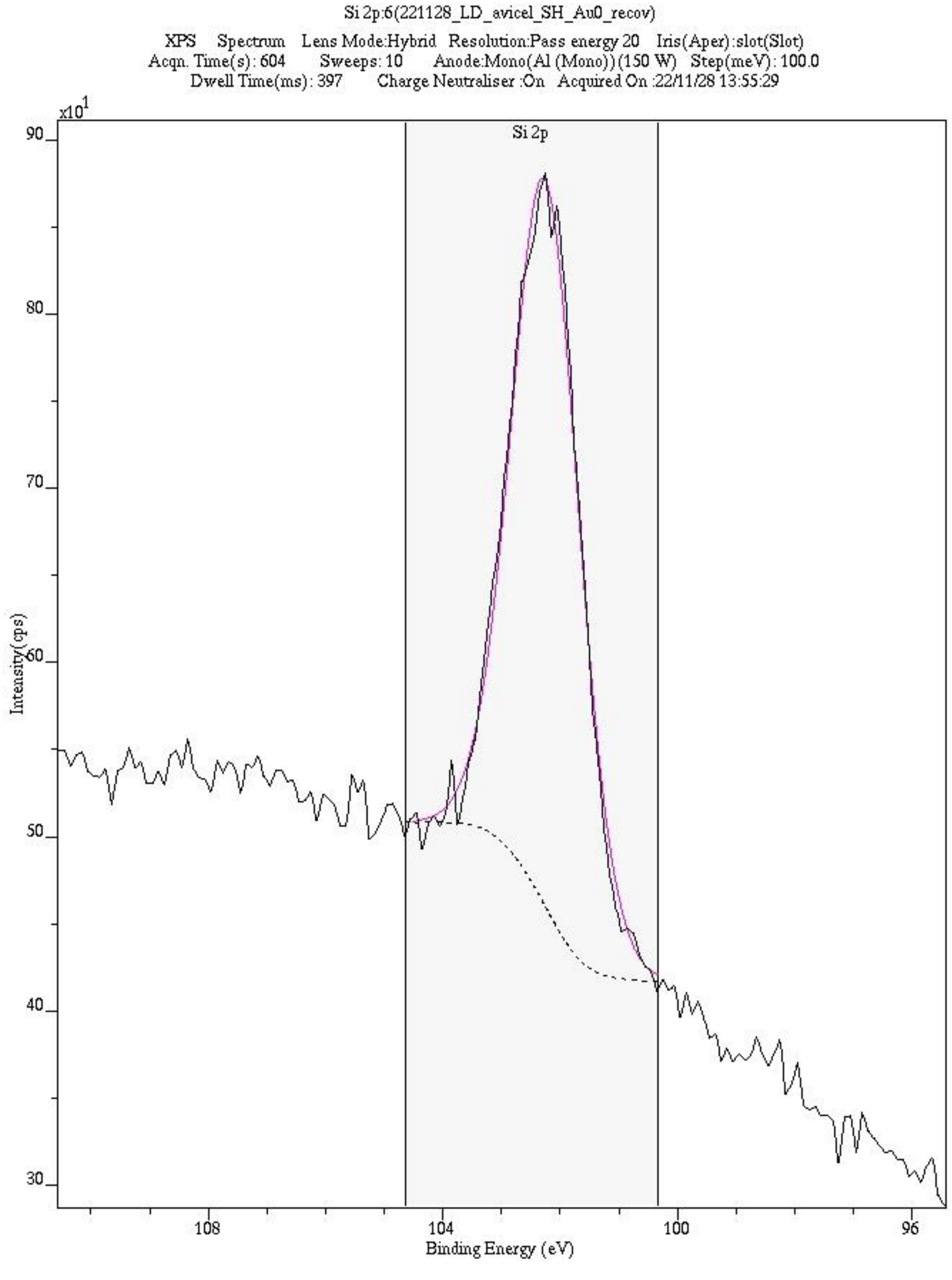

Figure S38. XPS spectrum of Si2p for MCC-McP-Au^0 recycled after reaction.

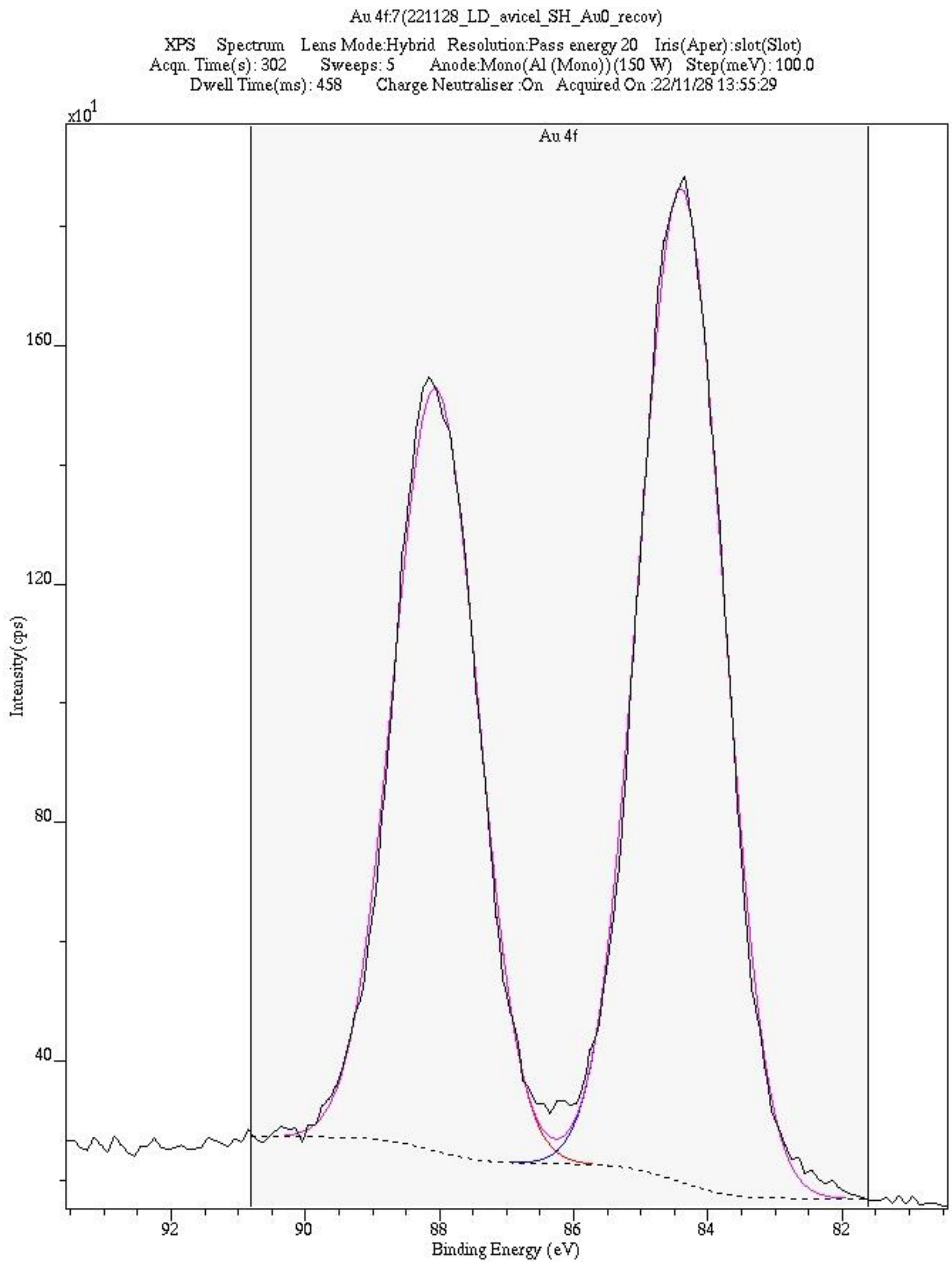

Figure S39. XPS spectrum of Auf4 for MCC-McP-Au^0 recycled after reaction.

References

(1) Conde, N.; SanMartin, R.; Herrero M. T.; Domínguez E. Palladium NNC Pincer Complex as an Efficient Catalyst for the Cycloisomerization of Alkynoic Acids. *Adv. Synth Catal.* **2016**, *358*, 3283-3292.
(2) Mindt T. L.; Schibli R. Cu(I)-Catalyzed Intramolecular Cyclization of Alkynoic Acids in Aqueous Media: A "Click Side Reaction". *J. Org. Chem.* **2007**, *72*, 10247-10250.
(3) Yuan, N.; Gudmundsson, A.; Gustafson, K. P. J.; Oschmann, M.; Tai, C.-W.; Persson, I.; Zou, X.; Verho, O.; Bajnóczi, E. J.; Bäckvall, J.-E. Investigation of the Deactivation and Reactivation Mechanism of a Heterogeneous Palladium(II) Catalyst in the Cycloisomerization of Acetylenic Acids by In Situ XAS. *ACS Catal.* **2021**, *11*, 2999–3008.
(4) (a) Li, M.-B.; Svensson Grape, E.; Bäckvall, J.-E. Palladium-Catalyzed Stereospecific Oxidative Cascade Reaction of Allenes for the Construction of Pyrrole Rings: Control of Reactivity and Selectivity. *ACS Catal.* **2019**, *9*, 5184–5190. (b) Zheng, Z.; Deiana, L.; Posevins, D.; Rafi, A.; Zhang, K.; Johansson, M. G.; Tai, C.-W.; Córdova, A.; Bäckvall, J.-E. Efficient Heterogeneous Copper-Catalyzed Alder-Ene Reaction of Allenyna you can see it was not sent out for reviewmides to Pyrrolines. *ACS Catal.* **2022**, *12*, *3*, 1791–1796.
(5) Eriksson, K.; Verho, O.; Nyholm, L.; Oscarsson, S.; Bäckvall, J.E. Dispersed Gold Nanoparticles Supported in the Pores of Siliceous Mesocellular Foam: A Catalyst for Cycloisomerization of Alkynoic Acids to γ-Alkylidene Lactones. *Eur. J. Org. Chem.* **2015**, 2250–2255.
